# An efficient visualization tool for the analysis of protein mutation matrices

**DOI:** 10.1186/1471-2105-9-218

**Published:** 2008-04-28

**Authors:** Maria Pamela C David, Carlo M Lapid, Vincent Ricardo M Daria

**Affiliations:** 1 Computational Science Research Center, University of the Philippines, Diliman 1101, Philippines; 2 Department of Computer Science, University of the Philippines, Diliman 1101, Philippines; 3 Advanced Science and Technology Institute, University of the Philippines, Diliman 1101, Philippines; 4 National Institute of Physics, University of the Philippines, Diliman 1101, Philippines

## Abstract

**Background:**

It is useful to develop a tool that would effectively describe protein mutation matrices specifically geared towards the identification of mutations that produce either wanted or unwanted effects, such as an increase or decrease in affinity, or a predisposition towards misfolding. Here, we describe a tool where such mutations are efficiently identified, categorized and visualized. To categorize the mutations, amino acids in a mutation matrix are arrang according to one of three sets of physicochemical characteristics, namely hydrophilicity, size and polarizability, and charge and polarity. The magnitude and frequences of mutations for an alignment are subsequently described using color information and scaling factors.

**Results:**

To illustrate the capabilities of our approach, the technique is used to visualize and to compare mutation patterns in evolving sequences with diametrically opposite characteristics. Results show the emergence of distinct patterns not immediately discernible from the raw matrices.

**Conclusion:**

Our technique enables effective categorization and visualization of mutations by using specifically-arranged mutation matrices. This tool has a number of possible applications in protein engineering, notably in simplifying the identification of mutations and/or mutation trends that are associated with specific engineered protein characteristics and behavior.

## Background

Mutation matrices have been frequently used to describe measures of physicochemical similarities among amino acids. Dayhoff *et al*. initially introduced the use of the mutation matrix, which was constructed from the phylogenetic analysis of 71 proteins with at least 85% pairwise sequence identity [1]. They observed point mutations in the matrices resulting from both the mutation of the gene itself, and the subsequent acceptance of the mutation, possibly as a predominant form. Not all possible replacements for an amino acid are acceptable, and the group of acceptable mutations vary from one protein family to another [1]. The Dayhoff matrix still ranks among the widely-used scoring schemes for generating multiple alignments, although there have been several modifications, such as the use of a larger number of more divergent protein sequences, as well as the generation of separate log-odds matrices for soluble and non-soluble proteins [2].

It remains difficult, however, to evaluate the effects of mutations in a set of related, constantly evolving proteins. It is possible to use criteria derived from phylogenetic data to analyze the implications of changes in a given environment using a combination of data [3-6]. Alternately, it would also be possible to extend the concept of mutation matrices by directing its generation towards the identification of naturally-occurring mutations that enhance the function of a protein by imbuing it with a structure that is more suited to its function and/or by increasing its potential for forming necessary chemical interactions [7-10]. We have previously designed an algorithm that identifies naturally-occurring mutations that enhance the function of a group of proteins by imbuing it with a structure that is more suited to its function and/or by increasing its potential for forming necessary chemical interactions; it would be useful to generate such matrices with reference to specific characteristics such as hydrophilicity, size and polarizability, and charge and polarity, and/or with reference to structural characteristics, such as residue exposure to solvent. Nevertheless, it is difficult to identify trends from raw mutation data, especially if the matrix was generated from a large number of sequences, and may consequently be more prone to noise.

Here, we present a visualization technique that specifically addresses the problem of gathering useful data from mutation matrices through the use of color and scaling. Visualization techniques for a very wide range of scientific disciplines have evolved in order to address the need for efficiently extracting data from datasets that are constantly growing in size and complexity. In the specific domain of protein analysis, these include Protein Data Bank (PDB) Sum, which gives an overview of all structures deposited in PDB; Protein explorer, which allows users to view 3D structure models, and Sequence to and within graphics (STING), which is actually a suite of programs useful for the comprehensive analysis of interrelationships between protein sequence, structure, function and stability. Our proposed scheme allows for effective categorization of mutations through the arrangement of amino acids in the matrix according to one of three sets of physicochemical characteristics. We also demonstrate an extension of the technique for comparing mutation patterns in evolving sequences with diametrically opposite characteristics. Our results show the emergence of distinct patterns not immediately discernible from the raw matrices. Finally, we demonstrate several applications of this scheme in protein engineering.

## Methods

### Matrix generation

**Figure 1:**
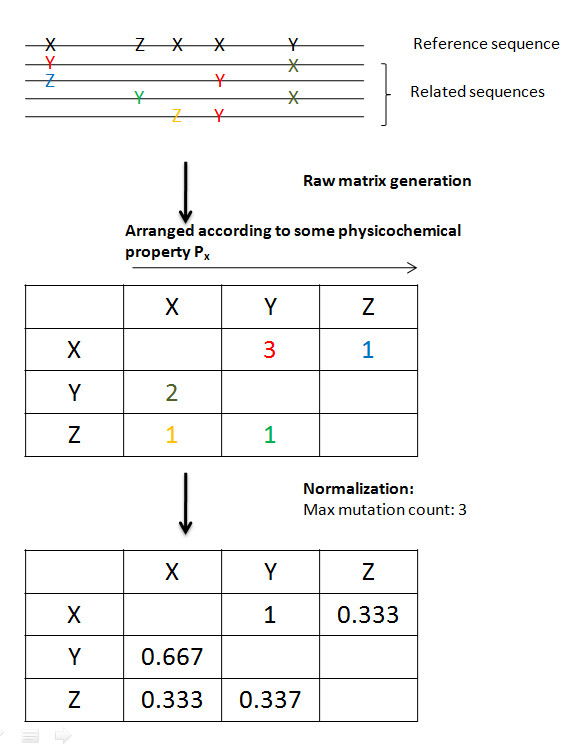
**Mutation matrix generation.** A raw mutation matrix is essentially a summary of the counts of all mutations from an amino acid in the reference sequence to every other amino acid in the sequences being compared to the reference. Its normalized equivalent is generated by dividing all the values in the matrix by the highest value found therein. Amino acids in the matrices are always arranged based on a particular physicochemical property. Normalization is done by dividing all entries in the matrix by the highest mutation frequency found in it. For a single alignment, it is possible to generate either one or multiple matrices, depending on the level of analysis that one wishes to subject it to.

Mutation matrices for four different protein datasets whose behaviors are influenced by amino acid variability, namely high affinity antibodies (anti-thyroid peroxidase antibodies, K_D _= 10^-9^), amyloidogenic light chain antibodies, hemagglutinin H5, and olfactory receptors (OR), were generated using a PERL script as described in David *et al*. [7]. Briefly, an alignment is constructed using related sequences and an appropriate reference sequence. The characteristics of the alignments used in this paper are summarized in Table 1. Currently, alignments can be constructed from sequences obtained from the NCBI using third-party software like ClustalW. Alternately, candidate sequences for an alignment are obtained directly from literature, then aligned using third-party software^1^. All mutations from the reference sequence are subsequently quantified with respect to some functional and/or structural characteristic (i.e. mutations that occur in buried residues are separated from those that occur in solvent-exposed residues). Slight modifications are made for the non-antibody sequences. In such cases, the reference sequence is either chosen based on the likelihood that it is the least divergent from the parent sequence, or if it possesses a property exclusive to it. For instance, in the case of the OR set, these include sequences that bind exclusively to long chain alcohols, paired against all sequences that exclusively bind short chain alcohols, or vice versa. All subsequences that may have been primer-derived (i.e. first five amino acids) are disregarded in the analysis. For the succeeding discussion, all matrix diagonals (which contain amino acid retention data) are disregarded. Matrices are arranged with respect to a physicochemical property (Table 2), and are normalized by dividing all values with the highest raw value in the matrix. A diagrammatic representation of the matrix generation process is shown in Fig. 1. For a single alignment, it is possible to generate either one or multiple matrices, depending on the level of analysis that one wishes to subject it to. For instance, if one simply wishes to distinguish between the mutation patterns in buried and exposed residues, one only needs to generate separate matrices for these. If there is a need to distinguish the mutation patterns found in buried residues in a total number of n_1 _regions from those found in buried residues in a total number of n_2 _regions, as well as from exposed residues in a total number of n_3 _regions and exposed residues in a total number of n_4 _regions, one would generate a total of n_1 _+ n_2 _+ n_3 _+ n_4 _matrices. A good example to illustrate this case is antibody sequence analysis, where one has to distinguish between mutation patterns in the complementarity determining regions (CDRs) and framework regions (FRs), and among its subsets (e.g. buried vs. exposed residues in the CDRs). A possible extreme case would be that mutation matrices will be generated for each position in an alignment.

**Table 1:** Characteristics of sample alignments.

Sequence Set	Reference sequence Characteristics	Derived sequences	Sample set (NCBI access code numbers)
Antibody (anti-TPO)	Low affinity antibody, usually a germline sequence	High-affinity germline derivatives directed against a single epitope	**Reference sequence:** Z22191 **Derivatives: **AAD09370, AAD29291, AAD29292, AAD29293, P06888
Antibody (amyloidogenic vs. non-amyloidogenic)	Germline sequence	**Set 1: **Amyloid-forming derivatives of the germline sequence	
		**Set 2: **Non-amyloid-forming, high-affinity derivatives of the germline sequence	
Hemagglutinin H5	Random hemagglutinin H5 sequence	All other available hemagglutinin H5 sequences	
Olfactory receptors	Sequence that recognizes some functional group X	Sequences that recognize some functional group Y whose characteristics are diametrically opposed to the characteristics of X, or which are not related to the characteristics of X	**Reference sequence:** NP_001011738.1 (short-chain alcohol-binding OR) **Derivatives **(long-chain alcohol-binding ORs): NP_064686.1 AAD27596.2 NP_038648.2 NP_667256.1 AAK00590.1

**Table 2:** Physicochemical property scales for naturally occurring amino acids.

Amino Acid	Hydrophilicity [12]	Size and Polarizability [12]	Charge and Polarity [13]
Ala (A)	0.24	-2.32	8.10
Arg (R)	3.52	2.5	10.5
Asn (N)	3.05	1.62	11.6
Asp (D)	3.98	0.93	13.0
Cys (C)	0.84	-1.67	5.5
Gln (Q)	1.75	0.5	10.5
Glu (E)	3.11	0.26	12.3
Gly (G)	2.05	-4.06	9.0
His (H)	2.47	1.95	10.4
Ile (I)	-3.89	-1.73	5.2
Leu (L)	-4.28	-1.3	4.9
Lys (K)	2.29	0.89	11.3
Met (M)	-2.85	-0.22	5.7
Phe (F)	-4.22	1.94	5.2
Pro (P)	-1.66	0.27	8.0
Ser (S)	2.39	-1.07	9.2
Thr (T)	0.75	-2.18	8.6
Trp (W)	-4.36	3.94	5.4
Tyr (Y)	-2.54	2.44	6.2
Val (V)	-2.59	-2.64	5.9

In the text, amino acids are identified using both three-letter and single-letter codes. A total of 319 sequences were analyzed (82 hemagglutinin H5, 7 olfactory receptor sequences, 150 amyloidogenic light chains and 80 non-amyloidogenic antibody sequences from a total of 15 germlines); these sequences were obtained either from the NCBI or from the papers in which these were published [11]. Sequences are identified by its Genbank accession number, when applicable [see Additional file 1].

### Matrix scaling and visualization

**Figure 2:**
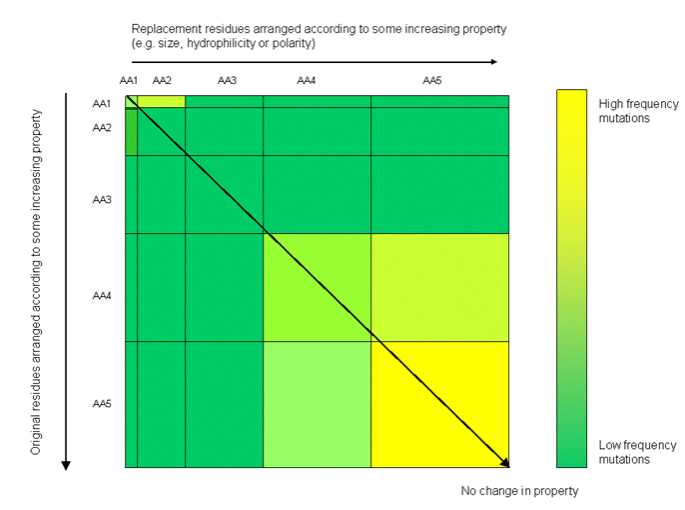
**Matrix scaling.** The size of the cells in the mutation matrix is proportional to the numeric quantity of some property (e.g. size, hydrophilicity, or polarity) associated with each amino acid. The color of each cell corresponds to the frequency at which each mutation (or conservation) occurs with respect to the reference sequence.

The proposed visualization scheme is demonstrated by developing a fully-automated visualization tool that allows users to determine the most predominant mutations and the approximate physico-chemical property changes, based on the characteristics indicated in De Genst et al. and Grantham [12,13], that result from these, based on color information and the size of a matrix square. The tool is developed using the Visualization Toolkit (vTk), Tool Command Language and the Tk graphical user interface toolkit (Tcl/Tk) [14,15]. A conceptual representation of a mutation matrix is shown in Fig. 2. Both the original and replacement amino acid residues are arranged in increasing order of a given property (i.e. for size and polarizability, amino acids are arranged from smallest to largest). The matrix component size reflects the property of the amino acid being described, as well as the degree of change involved on the occurrence of some mutation. The diagonal signifies no change; values to the left of the diagonal indicate a decrease in some property, while those on the right indicate an increase in the same property. The amount of change that results from a mutation corresponds to the difference in areas between some mutation cell, (AA_m_, AA_n_, m ≠ n) and the cell of its non-mutated counterpart, (AA_m_, AA_m_). For instance, assuming that Fig. 2 represents a size matrix, AA1, the smallest amino acid, mutates to AA2. Since this mutation is above the diagonal, it indicates an increase in size, with the increase proportional to the difference of the areas of the squares occupied by the cell defined by (AA1, AA2) and that defined by (AA1, AA1).

### Matrix comparison

A second tool that facilitates the automatic detection of mutations exclusive to a single matrix is constructed for applications that require the comparison of mutation trends in sequence sets with diametrically opposed characteristics (e.g. amyloidogenic vs. non-amyloidogenic matrices). A non-Boolean adaptation of the exclusive-OR (XOR) function is used in order to generate a visual superimposition of mutations that are exclusive to either matrix (Table 3). Mutations exclusive to either matrix can be identified based on their intensities, but the frequency of these mutations are not reflected in the resulting image. A complementary tool that identifies overlapping regions can also be generated.

**Table 3:** Matrix comparison visualization function

	Matrix 2 (+)	Matrix 2 (-)
Matrix 1 (+)	0	1
Matrix 1 (-)	0.5	0

## Results and Discussion

### Quadrant-based trend mapping

**Figure 3:**
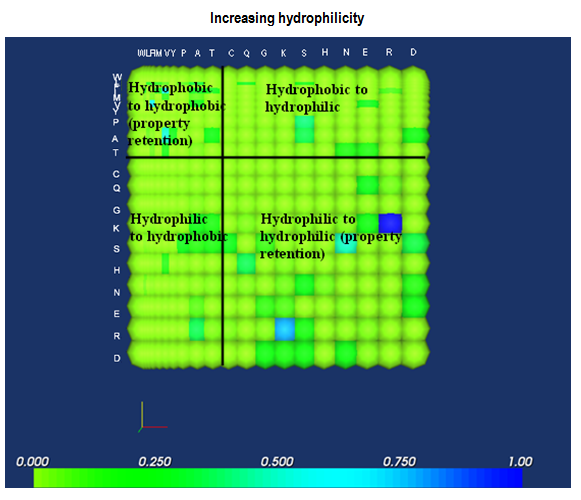
**Representative hydrophilicity mutation matrix.** Elementary analysis may be performed by subdividing the matrix into four quadrants, where mutations in the second and fourth quadrants may be generally associated with more conservative mutations than those found in the first and third quadrants.

**Figure 4:**
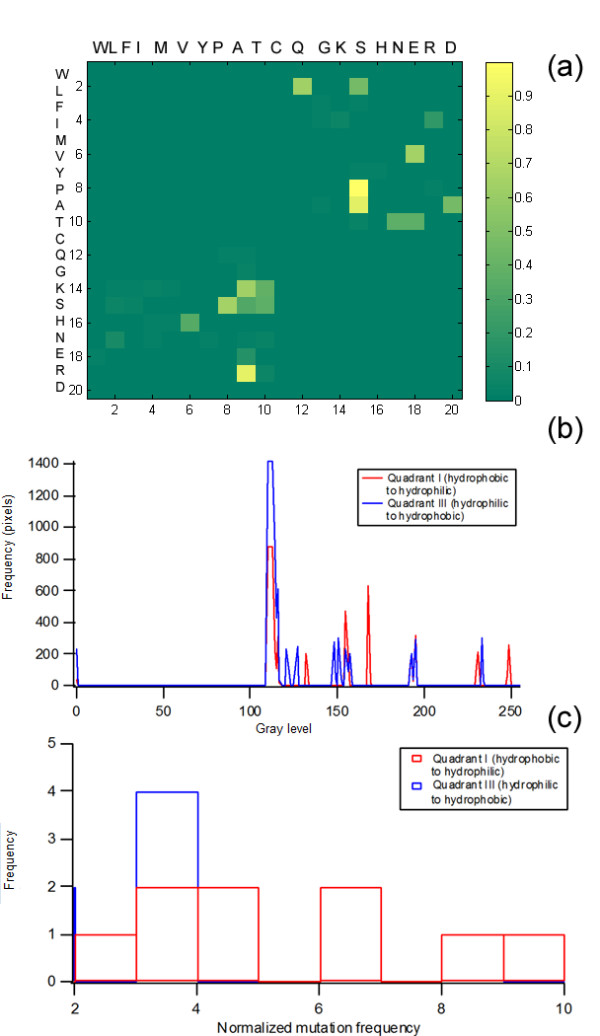
**Mutation matrix subsets. **Quadrants I and III of the mutation matrix shown in figure 3 were reproduced in order to demonstrate trends associated with these (A). Here, it is more evident that the most prominent mutations are located in Quadrant I (hydrophobic to hydrophilic mutations). The generation of a 256-bin histogram for the grayscale equivalent of the image (B), as well as a 10-bin histogram for the raw data (C) indicates that this is, in fact, the case.

The direct translation of a 20 × 20 amino acid mutation matrix into its equivalent intensity map would allow immediate extraction of general trends. For instance, a matrix of amino acids arranged in increasing order of hydrophilicity can be subdivided into the four Cartesian planes, each of which representing a particular behavior (Fig. 3). The diagonal, as well as the second and fourth quadrants, can be generally associated with property retention, while mutations associated with more drastic changes are in the first and third quadrants. In the example shown, most of the mutations are concentrated in the second and fourth quadrants, indicating that property conservation is an integral feature in the evolution of the set of proteins analyzed. Visually, however, the mutations in the first and third quadrants could not be easily compared. A possible strategy for handling such cases would be to limit visualization to selected quadrants, and to eliminate scaling. Figure 4A shows the partial matrix that corresponds to that in Fig. 3; here, it becomes more evident that hydrophobic to hydrophilic mutations (Quadrant I) are more favored than hydrophilic to hydrophobic mutations (Quadrant III). A histogram of the the 8-bit grayscale image equivalent indicates that lighter-colored pixels are more predominant in Quadrant I (Fig. 4B) than in Quadrant III. An analysis of the raw data (Fig. 4C) similarly indicates the prevalance of mutations with higher frequencies in Quadrant I.

### Applications in protein engineering

#### Application 1: Generation of synthetic antibodies

Antibodies rank among the most important therapeutic protein engineering targets. Synthetic antibodies, notably humanized antibodies or antibodies with improved affinities for its cognate ligands are in demand for cancer therapy and immunosuppression (IL-2, anti-CD33, etc.). Design issues in both cases include incorporating the necessary changes in order to alter molecule behavior/interactivity potential while maintaining or enhancing molecule stability, as well as maintaining the general structure [16]. It is also important that the designed molecule will not have a high potential for misfolding (i.e. would have minimal amyloidogenic potential), since this may result in pathogenicity.

In a related study [7], the design principles may be derived from the analysis of naturally-occurring mutation patterns in data sets associated with certain characteristics like those that may be derived from high affinity antibodies. This may be extended further through the comparison of data sets having diametrically opposed characteristics. For instance, comparison of mutations occurring in high affinity antibodies versus those with lower affinity suggests mutation patterns responsible for favorable binding. Similarly, comparing mutations that occur in amyloidogenic and non-amyloidogenic antibodies allows for the identification of some mutations that are probably responsible for misfolding. These mutations should subsequently be avoided in protein design. In order to derive these data, however, it is important to have a more convenient mode of representation than a raw matrix.

**Figure 5:**
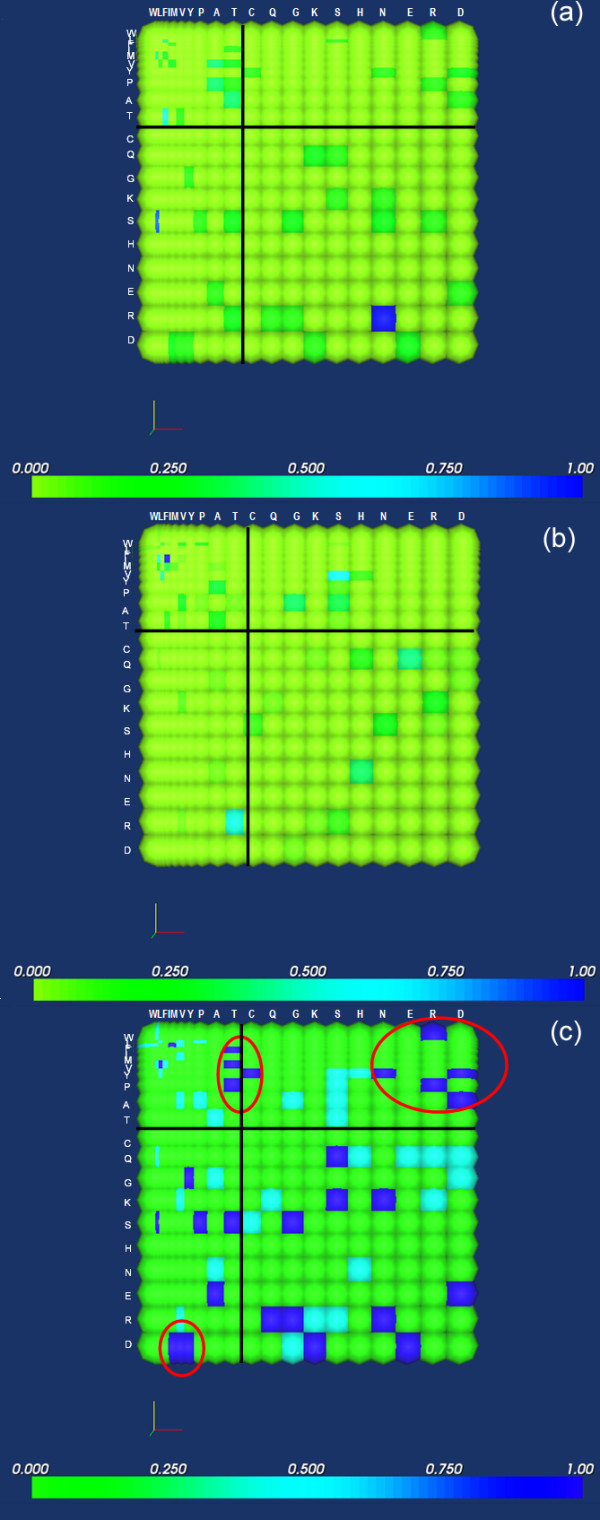
**Mutation patterns in amyloidogenic (A) and non-amyloidogenic (B) buried framework residues. **Amino acids were arranged by increasing hydrophilicity. These matrices were compared to identify the characteristics of mutations exclusively associated with either matrix (Fig. 4C). Mutations that occur exclusively in amyloidogenic sequences are in dark blue, while those associated with non-amyloidogenic sequences are in aqua. Encircled regions correspond to mutation clusters that appear to be predominantly associated with amyloidosis.

Figure 5 summarizes mutation patterns in high-affinity non-amyloidogenic buried framework residues (A), corresponding mutations in amyloidogenic antibodies derived from the same germlines (B), and a visual comparison of mutations, where these are identified based on their exclusive appearance in one group of proteins (C). Buried framework residues were chosen for this analysis since these are particularly important for antibody structure, and have a greater tendency to be implicated in amyloidosis. Hydrophilicity matrices were generated in order to derive trends that may cause drastic changes in amino acid exposure patterns, and subsequently, structure destabilization.

It is immediately evident that mutations are more varied and distributed in amyloidogenic (Fig. 5A) than in its non-amyloidogenic counterparts (Fig. 5B). Furthermore, most of the mutations that occur naturally in non-amyloidogenic sequences are concentrated near the diagonal or in the second and fourth quadrants, indicating a greater propensity for property conservation rather than property change, which is expected for FRs. This is not the case with mutations in amyloidogenic sequences, where a number of these are distributed across the first and third quadrants (Fig. 5B); a comparison of the matrices using the method described previously indicates that a number of the mutations exclusive to amyloid-formers involve maximal changes (encircled clusters, Fig. 5C) such as a Trp to Arg mutation or an Asp to Met mutation, which may cause a change in residue exposure at the position in which these mutations occur. Clearly, since these mutations are associated with amyloid formation, a direct application for these results is the general avoidance of such substitutions in buried framework residues of engineered antibodies. It would be important to reiterate at this point, however, that the identification of mutations associated with amyloid formation would be dependent on the alignments used; presumably, the best way to perform an analysis for this purpose would be to obtain a set of amyloidogenic sequences, as well as a set of high-affinity antibody sequences, that are derived from the same germline as the sequence being targeted for engineering.

This idea could be extended by evaluating the potential use of a matrix generated from different sets of high-affinity derivatives as a guide for pinpointing inadvisable substitutions in antibody engineering. The premise is that all mutations retained in the affinity maturation of high-affinity antibodies, regardless of affinity, are representative of those that promote affinity increases by increasing the resulting interaction potential, structural stability, and/or plasticity of the resulting antibody [7].

**Figure 6:**
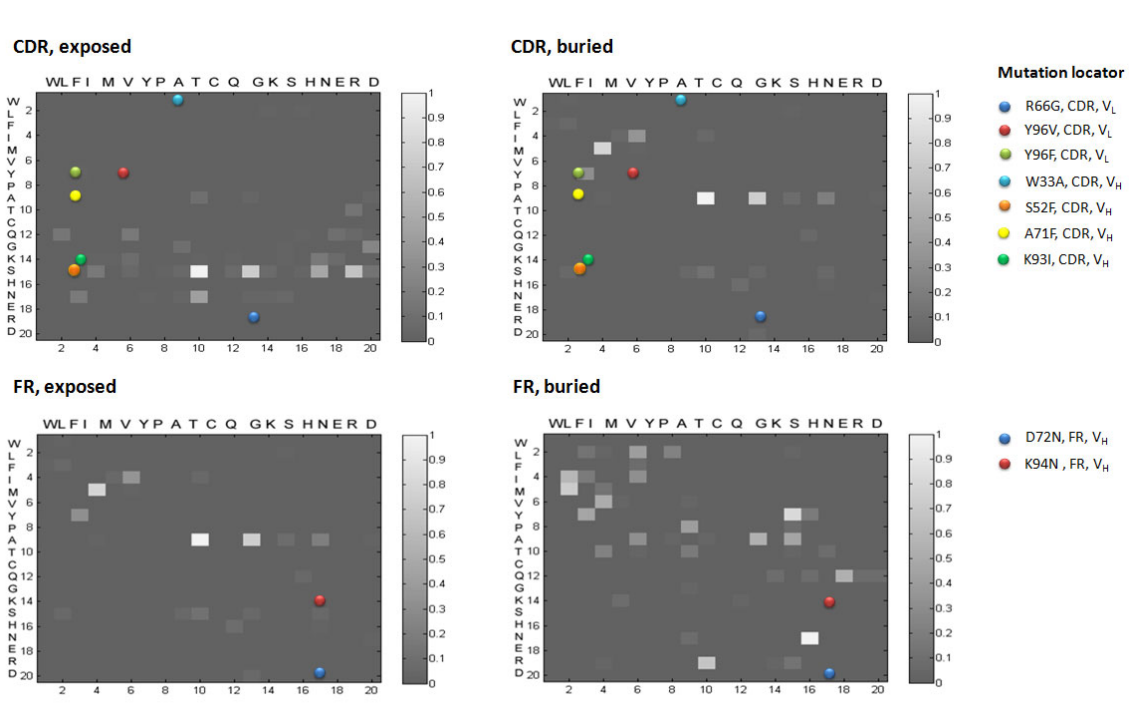
**Mutation patterns in CDRs and FRs of high-affinity antibody sequences. **The matrices shown were generated from the analysis of affinity-matured anti-thyroid peroxidase antibodies (anti-TPO, K_D _= 10^-9^) derived from six different germlines. No distinction between mutations in light and heavy chains were made. The colored spots indicate the positions of artificially-introduced mutations in engineered antibodies that were associated with decreased affinity (Table 4); each mutation is indicated in two different matrices, since the exposure patterns of these residues were not indicated in the original references. These mutations were never observed for high-affinity antibodies. Unscaled, grayscale matrices were used to improve contrast.

Table 4 lists some of substitutions in engineered antibodies that have been implicated in either affinity decreases or improvement, while figure 6 shows mutation matrices for buried (a) and exposed (b) CDRs and buried (c) and exposed (d) FRs of high affinity antibodies. These matrices were derived from the analysis of affinity-matured anti-TPO sequences (K_D _= 10^-9^) previously reported in David et al. [7]. The locations of the mutations in table 4 are indicated in the matrices. From a visual analysis of these examples, it is clear that most of the mutations implicated in affinity decreases are those that rarely occur in the affinity maturation of high affinity antibodies, while the mutations that are associated with affinity increases occur with comparatively higher frequencies.

**Table 4:** Artificially-introduced mutations implicated in antibody affinity reduction

Antibody chain, Region	Mutation^2^	Antigen	Affinity reduction	Reference
Light Chain, CDR	R66G	Carcinoembryonic antigen (CEA)	Four-fold (R)	17
Light Chain, CDR	Y96V	Finrozole	> Ten-fold (R)	18
Light Chain, CDR	Y96F	Atrazine	Two-fold (R)	19
Heavy Chain, CDR	W33A	Finrozole, d-enantiomer	Five-fold (R)	18
Heavy Chain, CDR	S52F	Digoxin	Two-fold (R)	20
Heavy Chain, CDR	A71F	Tumor-associated glycoprotein 72 (TAG72)	12-fold (R)	21
Heavy Chain, CDR	K93I	TAG72	20-fold (R)	21
Heavy Chain, FR	D72N	CD30	Significantly decreased binding signal	22
Heavy Chain, FR	K94N	HyHEL-10	~Ten-fold(R)	23

^2 ^Written in the form original residue, followed by the position (Kabat notation), then the replacement residue

#### Application 2: Vaccine design

While some therapeutic strategies involve the rational design of antibodies, others involve the opposite: the deliberate modification or synthesis of the antigenic proteins and peptides to which they bind, for use as vaccines. Vaccine design can have a variety of purposes: to decrease toxicity of an antigen or remove otherwise unwanted effects; to increase binding affinity to antibodies and thus improve their effectiveness in eliciting an immune response; to heighten similarity with the most recently circulating strains of a particular pathogen; or to render more compatible with some method of delivery [24-27]. In each case, modifications of specific structural or physico-chemical features may be required in an antigen while others, such as binding affinity, are preserved.

**Figure 7:**
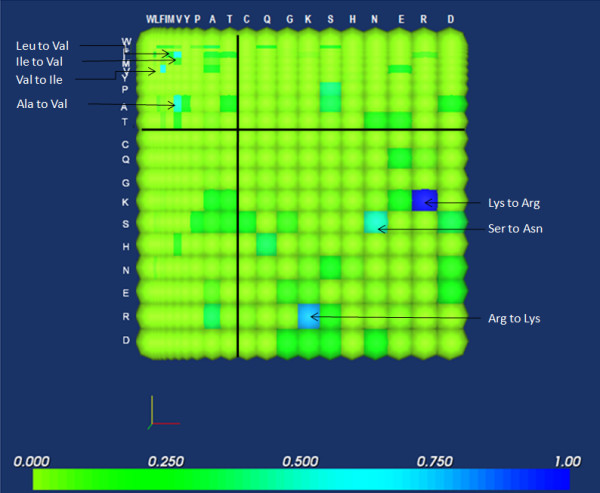
**Hemagglutinin H5 hydrophilicity mutation matrix. **Note the localization of the most prominent mutations in the second and fourth quadrants, indicating the predominance of mutations that tend to preserve hydrophilicity. These prominent mutations correspond to well-known conservative mutation pairs like Ile and Val and Lys and Arg; other prominent mutations are indicated in the figure.

Analysis of mutations that have occurred in antigens of rapidly mutating pathogens can provide clues as to how corresponding vaccines should be designed. An example of such a pathogen is the influenza virus: many studies have detailed how its dominant surface glycoproteins, neuraminidase and hemagglutinin, have evolved in response to immunological pressure [28]. Presented in Fig. 7 is the hydrophilicity mutation matrix for H5 hemagglutinin, belonging to the H5N1 subtype of avian influenza that has caused multiple outbreaks of poultry, as well as human infections and deaths, in recent years. It is evident that hydrophilicity is largely maintained, with most substitutions represented in the second and fourth quadrants, as well as near the diagonal. This suggests the importance of preserving this feature in targeted mutations. The matrix also reveals that certain pairs of amino acids, such as isoleucine and valine, or lysine and arginine, are to an extent interchangeable, as evidenced by the frequency of these mutations on both sides of the diagonal (Fig. 7). The other prominent mutations in the matrix are generally conservative.

#### Application 3: Olfactory receptor sequence analysis and biosensor design

Olfactory receptors (ORs) are transmembrane G protein coupled receptors that respond to a wide range of small, hydrophobic odorants. Unlike antibodies, which also exhibit highly varied ligand repertoires, OR do not undergo somatic hypermutation to achieve sequence diversity. Instead, diversity is conferred by the existence of roughly 1000 individual OR genes [29,30]. The transmembrane regions of the ORs are generally thought to be responsible for odorant binding.

An intriguing aspect of ORs is their involvement in combinatorial response, as opposed to the highly specific responses in the immune system. No single OR is exclusively associated with an odorant, and no odorant is associated with a single OR [29]. Applications of OR analysis include biosensor design. As an example, we generate a matrix from the transmembrane regions of two alignment sets where the reference sequences exclusively recognize short chain alcohols (i.e. 4 – 6 carbon alcohols), while the other sequences in the alignment exclusively bind long chain alcohols (i.e. 8 – 10 carbon alcohols). Presumably, all deviations from the reference sequence in the transmembrane domains contribute to the observed difference in specificity. We use size and polarizability as the basis for arranging the matrices, since the size of residues in the binding pockets would probably contribute significantly to the ability of a specific OR to accommodate the necessary number of odorant molecules to cause it to activate its associated neuron.

**Figure 8:**
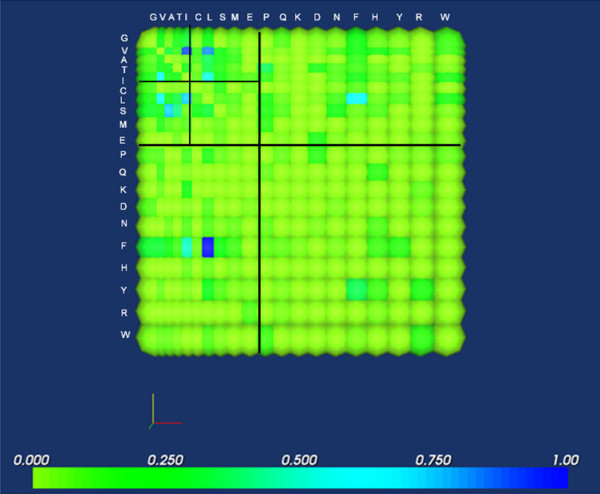
**Amino acid differences between short and long chain alcohol-binding olfactory receptors.** Most prominent differences are concentrated in the the first and third quadrants, indicating the preferential occurrence of small residues in the binding regions of ORs that exclusively recognize long chain alcohols. It is inferred that the smaller side chains allow the binding of bigger molecules.

Fig. 8 shows the composite mutation matrix representing deviations of long chain alcohol-binding OR from its short chain-binding counterparts. It is interesting to note that most of the prominent differences are concentrated in the first and third quadrants of the matrix. This indicates that most of the small residues can only be substituted with similarly small residues, and that larger residues in short chain alcohol binding-OR tend to have smaller counterparts in the long chain binders. The most prominent differences include the substitution of Phe with either Leu or Ile, which are both considerably smaller than Phe (Fig. 8). Quadrant I is further subdivided into quadrants as well; note that the most prominent substitutions here are those in Quadrant I-I and Quadrant I-III (i.e. those that preserve size, or those that tend to decrease residue size). These results are consistent with the probable requirement for smaller residues to accommodate the side chains of longer alcohols.

#### Applications of image processing techniques

**Figure 9:**
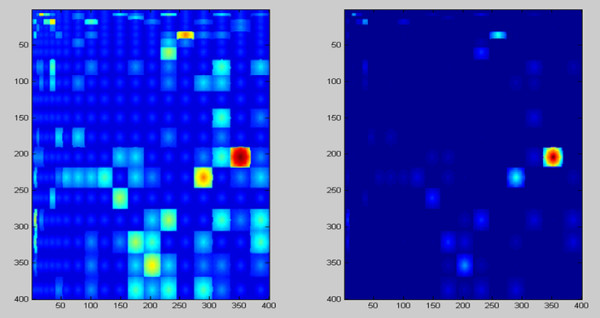
**Denoising using a fixed-size wavelet transformation.** The original image (A) was subjected to a fixed-size Mexican hat wavelet transformation (B) that effectively removed low frequency details.

Another distinct advantage of representing mutation matrices as images is the applicability of image processing techniques to improve the quality of the results, especially if these contain numerous artifacts [31]. In this particular application, noise is frequently caused by alignment errors or the use of distantly-related data (non-ideal alignments). In the hypothetical hydrophilicity matrix presented as an intensity map in Fig. 9A, it is comparatively difficult to identify trends, since mutations have roughly similar frequencies across the matrix.

Assuming that some of the data are, in fact, results of noise, it is possible to use techniques such as median filtering and wavelet transformations to lessen the effect of unwanted signal and to improve the visibility of a particular mutation. Median filtering is ideal for removing salt-and-pepper type noise [32-34]. This type of noise does not occur frequently in mutation matrices, where the frequencies may exhibit high variability within a quadrant. Median filtering will result in unwanted data averaging, and subsequently, data loss. It is important to note, however, that other methods as Gaussian blurring, discrete Fourier transformation or wavelet transformation are available for effectively denoising a matrix (Fig. 9B).

Apart from denoising, automated matrix analysis with a visual result is another possibility. Since our mapping translates the size of a matrix cell with respect to frequency, wavelet analysis, which is scalable, is an appropriate approach for characterizing a particular mutation. Unlike Fourier transformation, wavelet analysis is more controllable because one can specify both the frequencies to which the changes will be made and the extent of such changes. Here, we perform image processing by applying a convolution of the matrix image with an acircularly symmetric wavelet-like basis function given by:

(1) $$W\left(x,y\right)=\left(1-\frac{x^{2}+y^{2}}{\sigma^{2}}\right)\exp\left(-\frac{x^{2}+y^{2}}{\sigma^{2}}\right)$$

where *x *and *y *are image coordinates, while *σ *is the scaling parameter of the basis function. The convolution of the basis function and the matrix as represented in a two dimensional image, *I*(*x*,*y*) yields an output, *I*'(*x'*,*y'*) and can be described by

**Figure 10:**
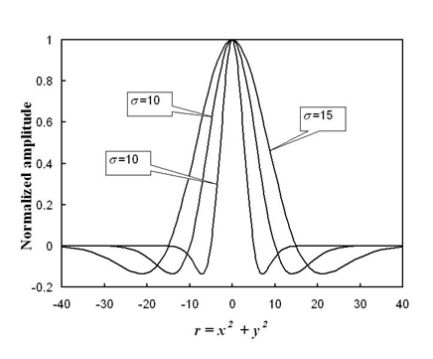
Profile of the circularly symmetric two-dimensional wavelet-like basis functions at various values of scaling parameter, *σ *= 5, 10 and 15.

(2) I'(x',y')=∑i=−N2N/2∑i=−N2N/2I(x−i,y−j)W(i,j)

where *N *is the size of the two-dimensional array of the circularly symmetric basis functions. Figure 10 plots the cross-sectional profile of the basis function.

**Figure 11:**
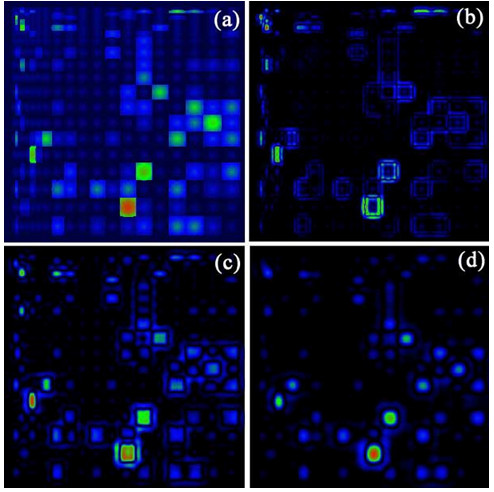
**(a) Image representing a two-dimensional mapping of mutation matrix.** Applying image processing routines to distinguish certain mutations is achieved by convolving the image with a wavelet-like basis function with scaling parameter (b) *σ *= 5, (c), *σ *= 10 and (d) *σ *= 15.

Figure 11 shows the results of the image processing routine applied on the input matrix (Fig. 11a). Use of a fixed-size basis function with *σ *= 5 results in enhancement of the peaks on the upper left quadarant. If the scale of the wavelet is customized to fit the general shape of cells in a particular matrix quadrant, trends are more prominent and no distortion occurs. In Fig. 11c, it is evident that mutations for this matrix are concentrated in the bottom-left and bottom-right quadrants. This indicates that for this given set of sequences, the hydrophobic residues in the reference sequence tend to mutate to hydrophilic ones, while those that are already hydrophilic frequently mutate to similarly hydrophilic amino acids. Fig. 11d shows the result when the image is convolved with a basis function with *σ *= 15. It highlights the mutation at the bottom-right quadrant with the other mutation peaks are discriminated. As shown in the manipulation of the image, different wavelet sizes result in different amounts of filtering. This specialized filtering will eventually enable users to perform highly selective manipulations on the matrix.

## Conclusion

We have proposed a visualization technique that facilitates faster analysis of mutation matrices through the use of color and scaling. Our technique enables effective categorization of mutations by arranging amino acids in the matrix according to one of three sets of physicochemical characteristics. The matrix visualization tool has a number of possible applications in protein engineering, notably in simplifying the identification of mutations and/or mutation trends that are associated with protein characteristics and behavior. Currently, the implementation is in the form of command-line operable standalone binaries in Perl (matrix generator) and vTk and Tcl/Tk (image generator). In the future, we plan to to make the programs available in a web application, and to link it to webservices of external systems (i.e. NCBI) to enable the user to create the alignments and generate the visualization through a single interface. The incorporation of an interface that would guide a user in generating the alignment is of particular importance, since the significance and validity of the results are dependent on the quality of the alignment used to generate the matrix in the first place.

## List of Abbreviations

OR: olfactory receptor; XOR: exclusive OR; AA: amino acid; vTk: Visualization Toolkit; Tcl/Tk: Tool Command Language and the Tk graphical user interface toolkit; CDR: complementarity-determining region; FR:  framework region.

## Availability and requirements

Additional material on the program source code for the visualization toolkit [see Additional file 2] may be requested from the authors; the matrix generator is written in PERL, and may be accessed at . A standalone program may also be requested from the authors. The visualization module was written in Tcl/Tk and vTk. To run the programs offline, the user must install PERL , Tcl/Tk  and vTk . User's manuals will be provided by the authors together with the software. *The program has been tested in Windows XP and selected Linux distributions*.

## Authors' contributions

MPCD conceptualized (with VRD) and generated the visualization program, obtained sequences and performed the analysis for the synthetic antibody design and olfactory receptor sequence analysis sections, and drafted bulk of the manuscript. CML obtained sequences and performed the analysis for the vaccine design section, wrote the corresponding section in the manuscript. VRD conceptualized the visualization application, generated the specifically-adapted wavelet function, and drafted this section of the manuscript.

## Note

^1^It is possible for a single alignment to contain sequences obtained from different references

## Supplementary Material

Additional file 1 Alignments used for the generation of the illustrated matrices. Click here for file

Additional file 2 Matrix visualization toolkit in vTk. Click here for file
