# Supplementary material for: An efficient visualization tool for the analysis of protein mutation matrices
Source: BMC Bioinformatics. 2008 Apr 28;9:218. doi: 10.1186/1471-2105-9-218 (PMC2390542; doi:10.1186/1471-2105-9-218)
Supplement: Additional file 1 — Alignments used for the generation of the illustrated matrices. [file 1471-2105-9-218-S1.doc]

**Hemagglutinin H5 sequences**

**AAT65209.1 MERIVLLLAIVSLVKSDQICIGYHANNSTEQVDTIMEKNVTVTHAQDILEKTHNGKLCDLDGVKPLILRDCSVAGWLLGNPMCDEFINVPEWSYIVEKASPANDLCYPGDFNDYQELK**

**AAA43083.1** MEEIVLLFAIVSLARSDQICIGYHANNSTKQVDTIMEKNVTVTHAQDILEKTHNGKLCSLNGVKPLILRDCSVAGWLLGNPMCDEFLNVPEWSYIVEKDNPVNGLCYPGDFNDYEELK

**AAR16155.1** MEEIVLLFAIVSLARSDQICIGYHANNSTKQVDTIMEKNVTVTHAQDILEKTHNGKLCSLNGVKPLILRDCSVAGWLLGNPMCDEFLNVPEWSYIVEKDNPVNGLCYPGDFNDYEELK

**CAF21870.1** -----LLFAIVSLVKSDQICIGYHANNSTEQVDTIMEKNVTVTHAQDILEKTHNGKLCSLNGVKPLILRDCSVAGWLLGNPMCDEFLNVPEWSYIVEKDSPINGLCYPGDFNDYEELK

**ABF58847.1** MEKIVLLFAIVSLVKSDQICIGYHANNSTEQVDTIMEKNVTVTHAQDILEKTHNGKLCDLDGVKPLILRDCSVAGWLLGNPMCDEFINVPEWSYIVEKANPVNDLCYPGDFNDYEELK

**AAC58998.1** ----------------DQICIGYHANNSTKQVDTIMEKNVTVTHAQDILEKEHNGKLCSLKGVRPLILKDCSVAGWLLGNPMCDEFLNVPEWSYIVEKDNPTNGLCYPGDFNDYEELK

**AAC58997.1** ----------------DQICIGYHANNSTEQVDTIMEKNVTVTHAQDILEKEHNGKLCSLKGVRPLILKDCSVAGWLLGNPMCDEFLNVPEWSYIVEKDNPVNGLCYPGDFNDYEELK

**AAC58996.1**----------------DQICIGYHANNSTEQVDTIMEKNVTVTHAQDILEKEHNGKLCSLKGVRPLILKDCSVAGWLLGNPMCDEFLNVPEWSYIVEKDNPVNGLCYPGDFNDYEELK

**AAC58994.1**----------------DQICIGYHANNSTEQVDTIMEKNVTVTHAQDILEKEHNGKLCSLKGVRPLILKDCSVAGWLLGNPMCDEFLNVPEWSYIVEKDNPINGLCYPGDFNDYEELK

**AAC58991.1**----------------DQICIGYHANNSTEQVDTIMEKNVTVTHAQDILEKEHNGKLCSLKGVRPLILKDCSVAGWLLGNPMCDEFLNVPEWSYIVEKDNPVSGLCYPGDLNDYEELK

**AAC34263.1** ----------------DQICIGYHANNSTKQVDTIMEKNVTVTHAQDILEKEHNGRLCSLKGVKPLILKDCSVAGWLLGNPMCDEFLNVPEWSYIVEKDNPANGLCYPGNFNDYEELK

**BAE48684.1** MEKIVLLFAIVSLVKSDQICIGYHANNSTEQVDTIMEKNVTVTHAQDILEKTHNGKLCSLNGVKPLILRDCSVAGWLLGNPMCDEFLNVPEWSYIVEKDSPINGLCYPGDFNDYEELK

**AAV91149.1** MEKIVLLLAIASLVKSDQICIGYHANNSTEQVDTIMEKNVTATHAQDILEKTHNGKLCDLDGVKPLILRDCSVAGWLLGNPMCDEFINVPEWSYIVEKASPANDLCYPGDFNDYEELK

**AAC58999.1** MERIVIALAIINIVKGDQICIGYHANNSTEQVDTIMEKNVTVTHAQDILEKEHNGKLCSLKGVRPLILKDCSVAGWLLGNPMCDEFLNVPEWSYIVEKDNPVNGLCYPGDFNDYEELK

**AAP72011.1**----------------DQICIGYHANNSTEQVDTIMEKNVTVTHAQDILEKEHNGKLCSLKGVRPLILKDCSVAGWLLGNPMCDEFLNVPEWSYIVEKDNPVNGLCYPGDFNDYEELK

**AAP72010.1**----------------DRICIGYHANNSTEQVDTIMEKNVTVTHAQDILEKEHNGKLCSLKGVRPLILKDCSVAGWLLGNPMCDAFLDVPEWSYIVEKDNPVNGLCYPGGFNDYEELK

**AAP72009.1**----------------DQICIGYHANNSTEQVDTIMEKNVTVTHAQDILEKEHNGKLCSLKGVRPLILKDCSVAGWLLGNPMCDEFLNVPEWSYIVEKDNPVNGLCYPGDFNDYEELK

**AAP72008.1**----------------DQICIGYHANNSTEQVDTIMEKNVTVTHAQDILEKEHNGKLCSLKGVRPLILKDCSVAGWLLGNPMCDEFLNVPEWSYIVEKDNPINGLCYPGDFNDYEELK

**AAP72007.1**----------------DQICIGYHANNSTEQVDTIMEKNVTVTHAQDILEKEHNGKLCSLKGVRPLILKDCSVAGWLLGNPMCDEFLNVPEWSYIVEKDNPVNGLCYPGDFNDYEELK

**AAP72011.1** ----------------DQICIGYHANNSTEQVDTIMEKNVTVTHAQDILEKEHNGKLCSLKGVRPLILKDCSVAGWLLGNPMCDEFLNVPEWSYIVEKDNPVNGLCYPGDFNDYEELK

**AAP72005.1**----------------DQICIGYHANNSTEQVDTIMEKNVTVTHAQDILEKEHNGKLCSLKGVRPLILKDCSVAGWLLGNPMCDEFLNVPEWSYIVEKDNPVNGLCYPGVFNDYEELK

**AAP72004.1**----------------DQICIGYHANNSTEQVDTIMEKNVTVTHAQDILEKEHNGKLCSLKGVRPLILKDCSIAGWLLGNPMCDEFLSVPEWSYIVEKDNPVNGLCYPGDFNDYEELK

**AAP72003.1** ----------------DQICIGYHANNSTEQVDTIMEKNVTVTHAQDILEKEHNGKLCSLKGVRPLILKDCSIAGWLLGNPMCDEFLNVPEWSYIVEKDNPVNGLCYPGDFNDYEELK

**AAP72002.1**----------------DQICIGYHANNSTEQVDTIMEKNVTVTHAQDILEKEHNGKLCSLKGVRPLILKDCSIAGWLLGNPMCDEFLNVPEWSYIVEKDNPVNGLCYPGDFNDYEELK

**AAP72001.1** MERIVIALAIISIVKGDQICIGYHANNSTEQVDTIMEKNVTVTHAQDILGKEHNGKLCSLKGVRPLTLKDCSVAGWLLGNPMCDEFLNVPEWSYIVEKDNPVNGLCYPGDFNDYEELK

**AAP72000.1** MERIVIVLAIISIIKGDQICIGYHANNSTEQVDTIMEKNVTVTHAQDILEKEHNGKLCSLKGVRPLILKDCSIAGWLLGNPMCDEFLNVPEWSYIVEKDNPVNGLCYPGDFNDYEELK

**AAP71998.1** MERIVIALAIISIVKGDQICIGYHANNSTEQVDTIMEKNVTVTHAQDILEKEHNGKLCSLKGVRPLILKDCSVAGWLLGNPMCDEFLNVPEWSYIVEKDNPANGLCYPGDFSDYEELK

**AAP71997.1** MERIVIALAIISIVKGDQICIGYHANNSTEQVDTIMEKNVTVTHAQDILEKEHNGKLCSLKGVRPLILKDCSVAGWLLGNPMCDEFLNVPEWSYIVEKDNPANGLCYPGDFSDYEELK

**AAP71996.1** MERIVIALAIISIVKGDQICIGYHANNSTEQVDTIMEKNVTVTHAQDILEKEHKGKLCSLKGVRPLILKDCSIAGWLLGNPMCDEFLNVPEWSYIVEKDNPANGLCYPGDFSDYEELK

**AAP71995.1** MERIVIALAIISIVKGDQICIGYHANNSTEQVDTIMEKNVTVTHAQDILEKEHNGKLCSLKGVRPLILKDCSIAGWLLGNPMCDEFLNVPEWSYIVEKDNPANGLCYPGDFSDYEELK

**AAN17270.1** MERIVIALAIISVVKGDQICIGYHANNSTEQVDTIMEKNVTVTHAQDILEKEHNGKLCSLKGVRPLILKDCSVAGWLLGNPMCDEFLNVPEWSYIVEKDNPVNGLCYPGDFNDYEELK

**AAN17269.1** MERIVIALAIISVVKGDQICIGYHANNSTEQVDTIMEKNVTVTHAQDILEKEHNGKLCSLKGVSPLILKDCSVAGWLLGNPMCDEFLNVPEWSYIVEKDNPVNGLCYPGDFNDYEELK

**AAN17256.1** MERIVIALAIISVVKGDQICIGYHANNSTEQVDTIMEKNVTVTHAQDILEKEHNGKLCSLKGVRPLILKDCSVAGWLLGNPMCDEFLNVPEWSYIVEKDNPVNGLCYPGDFNDYEELK

**AAN17267.1** MERIVIALAIISVVKGDQICIGYHANNSTEQVDTIMEKNVTVTHAQDILEKEHNGKLCSLKGVRPLILKDCSVAGWLLGNPMCDEFLNVPEWSYIVEKDNPVNGLCYPGDFNDYEELK

**AAN17266.1** MERIVIALAIISVVKGDQICIGYHANNSTEQVDTIMEKNVTVTHAQDILEKEHNGKLCSLKGVRPLILKDCSVAGWLLGNPMCDEFLNVPEWSYIVEKDNPVNGLCYPGDFNDYEELK

**AAN17265.1** MERIVIALAIISVVKGDQICIGYHANNSTEQVDTIMEKNVTVTHAQDILEKEHNGKLCSLKGVRPLILKDCSVAGWLLGNPMCDEFLNVPEWSYIVEKDNPVNGLCYPGDFNDYEELK

**AAN17264.1** MERIVIALAIISVVKGDQICIGYHANNSTEQVDTIMEKNVTVTHAQDILEKEHNGKLCSLKGVRPLILKDCSVAGWLLGNPMCDEFLNVPEWSYIVEKDNPVNGLCYPGDFNDYEELK

**AAN17263.1** MERIVIALAIISVVKGDQICIGYHANNSTEQVDTIMEKNVTVTHAQDILEKEHNGKLCSLKGVRPLILKDCSVAGWLLGNPMCDEFLNVPEWSYIVEKDNPVNGLCYPGDFNDYEELK

**AAN17262.1** MERIVIALAIISVVKGDQICIGYHANNSTEQVDTIMEKNVTVTHAQDILEKEHNGKLCSLKGVRPLILKDCSVAGWLLGNPMCDEFLNVPEWSYIVEKDNPVNGLCYPGDFNDYEELK

**AAN17261.1** MERIVIALAIISVVKGDQICIGYHANNSTEQVDTIMEKNVTVTHAQDILEKEHNGKLCSLKGVRPLILKDCSVAGWLLGNPMCDEFLNVPEWSYIVEKDNPVNGLCYPGDFNDYEELK

**AAN17260.1** MERIVIALAIISVVKGDQICIGYHANNSTEQVDTIMEKNVTVTHAQDILEKEHNGKLCSLKGVRPLILKDCSVAGWLLGNPMCDEFLNVPEWSYIVEKDNPVNGLCYPGDFNDYEELK

**AAN17259.1** MERIVIALAIISVVKGDQICIGYHANNSTEQVDTIMEKNVTVTHAQDILEKEHNGKLCSLKGVRPLILKDCSVAGWLLGNPMCDEFLNVPEWSYIVEKDNPVNGLCYPGDFNDYEELK

**AAN17258.1** MERIVIALAIISVVKGDQICIGYHANNSTEQVDTIMEKNVTVTHAQDILEKEHNGKLCSLKGVRPLILKDCSVAGWLLGNPMCDEFLNVPEWSYIVEKDNPVNGLCYPGDFNDYEELK

**AAN17257.1** MERIVIALAIISVVKGDQICIGYHANNSTEQVDTIMEKNVTVTHAQDI-EKEHNGKLCSLKGVRPLILKDCSVAGWLLGNPMCDEFLNVPEWSYIVEKDNPVNGLCYPGDFNDYEELK

**AAN17256.1** MERIVIALAIISVVKGDQICIGYHANNSTEQVDTIMEKNVTVTHAQDILEKEHNGKLCSLKGVRPLILKDCSVAGWLLGNPMCDEFLNVPEWSYIVEKDNPVNGLCYPGDFNDYEELK

**AAN17255.1** MERIVIALAIISVVKGDQICIGYHANNSTEQVDTIMEKNVTVTHAQDILEKEHNGKLCSLKGVRPLILKDCSVAGWLLGNPMCDEFLNVPEWSYIVEKDNPVNGLCYPGDFNDYEELK

**AAN17254.1** MERIVIALAIISVVKGDQICIGYHANNSTEQVDTIMEKNVTVTHAQDILEKEHNGKLCSLKGVRPLILKDCSVAGWLLGNPMCDEFLNVPEWSYIVEKDNPVNGLCYPGDFNDYEELK

**AAG38534.1** MEKIVLLLAIVSLVKSDQICIGYHANNSTEQVDTIMEKNVTVTHAQDILEKTHNGKLCSLNGVKPLILRDCSVAGWLLGNPMCDEFLNVPEWSYIVEKDNPVNGLCYPGDFNDYEELK

**AAF02306.1** MEKIVLLLATVSLVKSDQICIGYHANNSTEQVDTIMEKNVTVTHAQDILERTHNGKLCDLNGVKPLILRDCSVAGWLLGNPMCDEFINVPEWSYIVEKASPANDLCYPGNFNDYEELK

**AAL75847.1** MEKIVLLLAIVSLVKSDQICIGYHANNSTELVDTIMEKNVTVTHAQDILEKTHNGKLCDLDGVKPLILRDCSVAGWLLGNPMCDEFINVPEWSYIVEKANPANDLCYPGDFNDYEELK

**AAC32101.1** MEKIVLLLATVSLVKSDQICIGYHANNSTEQVDTIMEKNVTVTHAQDILERTHNGKLCDLNGVKPLILRDCSVAGWLLGNPMCDEFINVPEWSYIVEKASPANDLCYPGNFNDYEELK

**AAC32099.1** MEKIVLLLATVSLVKSDQICIGYHANNSTEQVDTIMEKNVTVTHAQDILERTHNGKLCDLNGVKPLILRDCSVAGWLLGNPMCDEFINVPEWSYIVEKASPANDLCYPGNFNDYEELK

**AAC32098.1** MEKIVLLLATVSLVKSDQICIGYHANNSTEQVDTIMEKNVTVTHAQDILERTHNGKLCDLNGVKPLILRDCSVAGWXLGNPMCDEFLNVPEWSYIVEKTSPANDLCYPGHFNDYEELK

**AAC32088.1** MEKTVLLLATVSLVKSDQICIGYHANNSTEQVDTIMEKNVTVTHAQDILERTHNGKLCDLNGVKPLILRDCSVAGWLLGNPMCDEFINVPEWSYIVEKASPANDLCYPGNFNDYEELK

**AAC32078.1** MEKIVLLLATVSLVKSDQICIGYHANNSTEQVDTIMEKNVTVTHAQDILERTHNGKLCDLNGVKPLILRDCSVAGWLLGNPMCDEFINVPEWSYIVEKASPANDLCYPGNFNDYEELK

**AAR99628.1** MEKTVLLLAIVSLVKSDQICIGYHANNSTEQVDTIMEKNVTVTHAQDILEKTHNGKLCDLDGVKPLILRDCSVAGWLLGNPMCDEFINVPEWSYIVEKANPANDLCYPGNFNDYEELK

**AAD13568.1** MEKIVLLLATVSLVKSDQICIGYHANNSTEQVDTIMEKNVTVTHAQDILERTHNGKLCDLNGVKPLILRDCSVAGWLLGNPMCDEFINVPEWSYIVEKASPANDLCYPGNFNDYEELK

**AAM49555.1** MEKIVLLLAIVSLVKSDQICIGYHANNSTEQVDTIMEKNVTVTHAQDILEKTHNGKLCDLDGVKPLILRDCSVAGWLLGNPMCDEFINVPEWSYIVEKANPANDLCYPGDFNDYEELK

**AAL31387.1** MEKIVLLLAVVSLVKSDQICIGYHANNSTEQVDTIMEKNVTVTHAQDILEKTHNGKLCDLDGVKPLILRDCSVAGWLLGNPMCDEFINVPEWSYIVEKASPANDLCYPGDFNDYEELK

**AAL75839.1** MEKIVLLLAIVSLVKSDQICIGYHANNSTEQVDTIMEKNVTVTHAQDILEKTHNGKLCDLDGVKPLILRDCSVAGWLLGNPMCDEFINAPEWSYIVEKASPANDLCYPGDFNNYEELK

**CAC28131.1** MEKIVLLLAIVSLVKSDQICIGYHANNSTEQVDTIMEKNVTVTHAQDILEKTHNGKLCSLNGVKPLILRDCSVAGWLLGNPMCDEFLNVPEWSYIVEKDNPVNGLCYPGDFNDYEELK

**AAA43082.1** MEEIVLLFAIVSLARSDQICIGYHANNSTKQVDTIMEKNVTVTHAQDILEKTHNGKLCSLNGVKPLILRDCSVAGWLLGNPMCDEFLNVPEWSYIVEKDNPVNGLCYPGDFNDYEELK

**AAA43083.1** MEEIVLLFAIVSLARSDQICIGYHANNSTKQVDTIMEKNVTVTHAQDILEKTHNGKLCSLNGVKPLILRDCSVAGWLLGNPMCDEFLNVPEWSYIVEKDNPVNGLCYPGDFNDYEELK

**AAC58999.1** MERIVIALAIINIVKGDQICIGYHANNSTEQVDTIMEKNVTVTHAQDILEKEHNGKLCSLKGVRPLILKDCSVAGWLLGNPMCDEFLNVPEWSYIVEKDNPVNGLCYPGDFNDYEELK

**AAR16155.1** MERMVIALAIISIVKGDQICIGYHANNSTEQVDTIMEKNVTVTHAQDILEKEHNGKLCSLKGVRPFILKDCSVAGWLLGNPMCDEFLNVPEWSYIVEKDNPVNGLCYPGDFNDYEELK

**AAD13574.1** MEKIVLLLAIVSLAESDQICIGHHANNSTEQVDTIMERNVTVTHAQDILEKTHNGKLCSLNGVKPLILRDCSVAGWLLGNPMCDEFLNVPEWSYIVEKDNPINGLCYPGDFNDYEELK

**AAY57197.1** MEKIVLLLAVVSLVRSDQICIGYHANNSTEQVDTIMEKNVTVTHAQDILEKTHNGKLCSLNGVKPLILRDCSVAGWLLGNPMCDEFLNVPEWSYIVEKDNPVNGLCYPGDFNDYEELK

**AAD13568.1** MEKIVLLLATVSLVKSDQICIGYHANNSTEQVDTIMEKNVTVTHAQDILERTHNGKLCDLNGVKPLILRDCSVAGWLLGNPMCDEFINVPEWSYIVEKASPANDLCYPGNFNDYEELK

**AAD13575.1** MERIVIALAIISVVKGDQICIGYHANNSTEQVDTIMEKNVTVTHAQDILEKEHNGKLCSLKGVRPLILKDCSVAGWLLGNPMCDEFLNVPEWSYIVEKDNPVNGLCYPGDFNDYEELK

**AAD13572.1** MERIVIALAIISVVKGDQICIGYHANNSTEQVDTIMEKNVTVTHAQDILEKEHNGKLCSLKGVRPLILKDCSVAGWLLGNPMCDEFLNVPEWSYIVEKDNPVNGLCYPGDFNDYEELK

**AAD13567.1** MEKIVLLLATVSLVKSDQICIGYHANNSTEQVDTIMEKNVTVTHAQDILERTHNGKLCDLNGVKPLILRDCSVAGWLLGNPMCDEFINVPEWSYIVEKASPANDLCYPGNFNDYEELK

**AAD13566.1** MEKIVLLLATVSLVKSDQICIGYHANNSTEQVDTIMEKNVTVTHAQDILERTHNGKLCDLNGVKPLILRDCSVAGWLLGNPMCDEFINVPEWSYIVEKASPANDLCYPGNFNDYEELK

**AAD13568.1** ----------------DQICIGYHANNSTEQVDTIMEKNVTVTHAQDILERTHNGKLCDLNGVKPLILRDCSVAGWLLGNPMCDEFINVPEWSYIVEKASPANDLCYPGNFNDYEELK

**AAA43082.1** MEEIVLLFAIVSLARSDQICIGYHANNSTKQVDTIMEKNVTVTHAQDILEKTHNGKLCSLNGVKPLILRDCSVAGWLLGNPMCDEFLNVPEWSYIVEKDNPVNGLCYPGDFNDYEELK

**AAA43083.1** MEEIVLLFAIVSLARSDQICIGYHANNSTKQVDTIMEKNVTVTHAQDILEKTHNGKLCSLNGVKPLILRDCSVAGWLLGNPMCDEFLNVPEWSYIVEKDNPVNGLCYPGDFNDYEELK

**AAG01225.1** MEKIVLLLAIVSLVKSDQICIGYHANNSTEQVDTIMEKNVTVTHAQDILEKTHNGKLCDLDGVKPLILRDCSVAGWLLGNPMCDEFINVPEWSYIVEKASPANDLCYPGDFNNYEELK

**AAG01215.1** MEKIVLLLAIVSLVKSDQICIGYHANNSTEQVDTIMEKNVTVTHAQDILEKTHNGKLCDLDGVKPLILRDCSVAGWLLGNPMCDEFINVPEWSYIVEKASPANDLCYPGDFNNYEELK

**AAG01205.1** MEKIVLLLAIVSLVKSDQICIGYHANNSTEQVDTIMEKNVTVTHAQDILEKTHNGKLCDLDGVKPLILRDCSVAGWLLGNPMCDEFINVPEWSYIVEKASPANDLCYPGDFNNYEELK

**AAG01195.1** MEKIVLLLAIVSLVKSDQICIGYHANNSTEQVDTIMEKNVTVTHAQDILEKTHNGKLCDLDGVKPLILRDCSVAGWLLGNPMCDEFINVPEWSYIVEKASPANDLCYPGDFNNYEELK

**AAD51927.1** MEKIVLLLAIVSLVKSDQICIGYHANNSTEQVDTIMEKNVTVTHAQDILEKTHNGKLCDLNGVKPLILRDCSVAGWLLGNPMCDEFINVPEWSYIVEKASPANDLCYPGDFNDYEELK

**AAF04720.1** MERIVIALAIISVVKGDQICIGYHANNSTEQVDTIMEKNVTVTHAQDILEKEHNGKLCSLKGVRPLILKDCSVAGWLLGNPMCDEFLNVPEWSYIVEKDNPVNGLCYPGDFNDYEELK

**AAF04719.1** MERIVIALAIISVVKGDQICIGYHANNSTEQVDTIMEKNVTVTHAQDILEKEHNGKLCSLKGVRPLILKDCSVAGWLLGNPMCDEFLNVPEWSYIVEKDNPVNGLCYPGDFNDYEELK

**AAT65209.1 HLLSRINHFEKIQIIPKSSWSNHEASSGVSSACPYNGKPSFFRNVVWLIKKNSAYPTIKRSXNNTNHEDLLVLWGIHHPNDAAEQTKLYQNPTTYIXXGTSTLNLRLVPKIATRSKVN**

**AAA43083.1** HLLSCTKHFEKIRIIPRDSWPNHEASLGVSSACPYNGRSSFFRNVVWLIKKNNAYPTIKRSYSNTNKEDLLILWGIHHPNDAAEQTKLYQNPTTYVSVGTSTLNQRSIPKIATRPKLN

**AAR16155.1** HLLSCTKHFEKIRIIPRDSWPNHEASSGVSSACPYNGRSSFFRNVVWLIKKDNAYPTIKRSYNNTNKEDLLILWGIHHPNDAAEQTKLYQNPTTYVSVGTSTLNQRSIPKIATRPKLN

**CAF21870.1** HLLSSTNHFEKIQIIPRSSWSDHDASSGVSSACPYHGRSSFFRNVVWLIKKNNAYPTIKRSYNNTNQEDLLVLWGIHHPNDAAEQTKLYQNPTTYVSVGTSTLNQRSVPEIATRPKVN

**ABF58847.1** HLLSRINHFEKIQIIPKSSWSSHEASLGVSSACPYQGKSSFFRNVVWLIKKNSTYPTIKRSYNNTNQEDLLVLWGIHHPNDAAEQTKLYQNPTTYISVGTSTLNQRLVPRIATRSKVN

**AAC58998.1** YLMSNTNHFEKIQIIPRNSWSNHDASSGVSSACPYNGRSSFFRSVVWLIKKSNVYPTIKRTYNNTNVEDLLILWGIHHPNDAAEQTELYQNSNTYVSVGTSTLNQRSIPEIATRPKVN

**AAC58997.1** HLMSSTNHFEKIQIIPRSSWSNHDASSGVSSACPYNGRSSFFRNVVWLIKKNNAYPTIKRTYNNTNVEDLLIIWGIHHPNDAAEQTNLYQNSNTYVSVGTSTLNQRSIPEIATRPKVN

**AAC58996.1** HLMSSTNHFEKIQIIPRSSWSNHDASSGVSSACPYNGRSSFFRNVVWLIKKNNAYPTIKRTYNNTNMEDLLILWGIHHPNDAAEQTKLYQNSNTYVSVGTSTLNQRSIPEIATRPKVN

**AAC58994.1** YLMSSTNHFEKIQIIPRSSWSNHDASSGVSSACPYNGRSSFFRNVVWLIKKNNAYPTIKRTYNNTNIEDLLILWGIHHPNDAAEQTKLYQNSNTYVSVGTSTLNQRSIPEIATRPKVN

**AAC58991.1** HLMSSTNHFEKIQIIPRSSWSNHDASSGVSSACPYNGRSSFFRNVVWLIKKNDAYPTIKRTYNNTNIEDLLILWGIHHPNDAAEQTKLYQNSNTYVSVGTSTLNQRSIPEIATRPKVN

**AAC34263.1** HLMSSTNHFEKIQIFPRSSWSNHDASSGVSSACPYNGRSSFFRNVVWLIKKNNVYRTIKRTYHNTNVEDLLILWGIHHPNDAAEQIKLYQNPNTYVSVGTSTLNQRSIPEIATRPKVN

**BAE48684.1** HLLSSTNHFEKIQIIPRSSWSNHDASSGVSSACPYNGRSSFFRNVVWLIKKNNAYPTIKRNYNNTNQEDLLVLWGIHHPNDATEQTKLYQNPTTYVSVGTSTLNQRSSPEIATRPKVN

**AAV91149.1** HLLSRINHFEKIQIIPKNSWSNHEASSGVSSACPYLGKPSFFRNVVWLIKKNSTYPTIKRSYNNTNQEDLLVLWGIHHPNDAAEQTKLYQNPTTYISVGTSTLNQRLVPKIATRSKVN

**AAC58999.1** HLMSSTNHFEKIQIIPRNSWSTHDASSGVSSACPYNGRSSFFRNVVWLIKKNNAYPTIKRTYNNTNVEDLLILWGIHHPNDAAEQTKLYQNSNTYVSVGTSTLNQRSIPEIATRPKVN

**AAP72011.1** HLMSSTNHFEKIQIIPRSSWSNHDASSGVSSACPYNGRSSFFRNVVWLIKKNNAYPTIKRTYNNTNVEDLLILWGIHHPNDAAEQTKLYQNSNTYVSVGTSTLNQRSIPEIATRPKVN

**AAP72010.1** HLMSNTNHVERIQIIPRSSWSNHDATSGVSSACPHNGRSSFFRNVVWLTKKNNVYRTVKRTYNNTNVEDLLILWGIHHPNDAAEQIKLYQNSNTYVSVGTSTLNKRSIPVIATRPKVN

**AAP72009.1** HLMSSTNHFEKIQIIPRSSWSNHDASSGVSSACPYHGRSSFFRNVVWLIKKNNAYPTIKRTYNNTNVEDLLIIWGIHHPNDAAEQTKLYQNSNTYVSVGTSTLNQRSIPEIATRPKVN

**AAP72008.1** HLMSSTNHFKKIQIIPRSSWSNHVASSGVSSACPYNGRSSFFRNVVWLIKKNNAYPTIKRTYNNTNVEDLLIIWGIHHPNDAAEQTKLYQNSNTYVSVGTSTLNQRSIPEIATRPKVN

**AAP72007.1** HLMSSTNHFEKIQIIPRSSWSNHDASSGVSSACPYNGRSSFFRNVVWLIKKNNAYPTIKRTYNNTNVEDLLILWGIHQPNDAAEQTKLYQNSNTYVSVGTSTLNQRSIPEIATRPKVN

**AAP72011.1** HLMSSTNHFEKIQIIPRSSWSNHDASSGVSSACPYNGRSSFFRNVVWLIKKNNAYPTIKRTYNNTNVEDLLILWGIHHPNDAAEQTKLYQNSNTYVSVGTSTLNQRSIPEIATRPKVN

**AAP72005.1** HLMSSTNHFEKIQIIPRSSWSNHDASSGVSSACPYNGRSSFFRNVVWLIKKNNAYPTIKRTYNNTNVEDLLILWGIHHPNDAAEQTKLYQNSNTYVSVGTSTLNQRSIPEIATRPKVN

**AAP72004.1** HLMSSTNHFEKIQIIPRSSWSNHDASSGVSSACPYNGRSSFFRNVVWLIKKNNAYPTIKRTYNNTNVEDLLIIWGIHHPNDAAEQTKLYQNSNTYVSVGTSTLNQRSIPEIAIRPKVN

**AAP72003.1** HLMSSTNHFEKIQMIPRSSWSNHDASSGVSSACPYNGRSSFFRNVVWLIKKNNAYPTIKRTYNNTNVEDLLIIWGIHHPNDAAEQTKLYQNSNTYVSVGTSTLNQRSIPEIATRPKVN

**AAP72002.1** HLMSSTNHFEKIQIIPRSSWSNHDASSGVSSACPYNGRSSFFRNVVWLIKKNNAYPTIKRTYNNTNVEDLLIIWGIHHPNDAAEQTKLYQNSNTSVSVGTSTLNQRSIPEIATRPKVN

**AAP72001.1** HLMSSTNHFEKIQIIPRSSWSNHDASSGVSSACPYDGRSSFFRNVVWLIKKNNAYPTIKRTYNNTNVEDLLILWGIHHPNDAAEQTKLYQNSNTYVSVGTSTLNQRSIPEIATRPKVN

**AAP72000.1** HLISSTNHFEKIQIIPRSSWSNHDASSGVSSACPYIGRSSFFRNVVWLIKKNNAYPTIKRTYNNTNVEDLLIIWGIHHPNDAAEQTKLYQNSNTYVSVGTSTLNQRSIPEIAIRPKVN

**AAP71998.1** HLMSSTNHFEKIQIIPRSSWSNHDASSGVSSACPYNGRSSFFRNVVWLIKKNNAYPTIKRTHSNTNVEDLLILWGIHHPNDAAEQTKLYQNSNAYVSVGTSTLNQRSIPEIATRPRVN

**AAP71997.1** HLMSSTNHFEKIQIIPRSSWSNHDASSGVSSACPYNGRSSFFRNVVWLIKKNNAYPTIKRTYNNTNVEDLLILWGIHHPNDAAEQTKLYQNSNAYVSVGTSTLNQRSIPEIATRPRVN

**AAP71996.1** HLMSSTNHFEKIQIIPRSSWSNHDASSGVSSACPYNGRSSFFRNVVWLIKKNNAYPTIKRTYNNTNVEDLLILWGIHHPNDAAEQTKLYQNSNAYVSVGTSTLNQRSIPEIATRPRVN

**AAP71995.1** HLMSSTNHFEKIQIIPRSSWSNHDASSGVSSACPYNGRSSFFRNVVWLIKKNNAYPTIKRTYNNTNVEDLLILWGIHHPNDAAEQTKLYQNSNAYVSVGTSTLNQRSIPEIATRPRVN

**AAN17270.1** HLMSSTNHFEKIQIIPRSSWSDHDASSGVSSACPYNGRSSFFRNVVWLITKNNAYPTIKRTYNNTNVEDLLILWGIHHPNDATEQTKLYQNSNTYVFVGTSTLNQRSIPEIATRPKVN

**AAN17269.1** HLMSSTNHFEKIQIIPRSSWSNHNASSGVSSACPYNGRSSFFRNVVWLIKKNNAYPTIKRTYNNTNVEDLLILWGIHHPNDATEQTKLYQNSNTYVFVGTSTLNQRSIPEIATRPKVN

**AAN17256.1** HLMSSTNHFEKIQIIPRSSWSNHDASSGVSSACPYNGRSSFFRNVVWLINKNNAYPTIKRTYNNTNVEDLLILWGIHHPNDATEQTKLYQNSNTYVFVGTSTLNQRSIPEIATRPKVN

**AAN17267.1** HLMSSTNHFEKIQIIPRSSWSNHDASSGVSSACPYNGRSSFFRNVVWLIKMNNAYPTIKRTYNNTNVEDLLILWGIHHPNDATEQTKLYQNSNTYVFVGTSTLNQRSIPEIATRPKVN

**AAN17266.1** HLMSSTNHFEKIQIIPRSSWSDHDASSGVSSACLYNGRSSFFRNVVWLITKNNAYPTIKRTYNNTNVEDLLILWGIHHPNDATEQTKLYQNSNTYVFVGTSTLNQRSIPEIATRPKVN

**AAN17265.1** HLMSSTNHFEKIQIIPRSSWSNHNASSGVSSACPYNGRPSFFRNVVWLIKKNNAYPTIKRTYNNTNVEDLLILWGIHHPNDATEQTKLYQNSNTYVFVGTSTLNQRSIPEIATRPKVN

**AAN17264.1** HLMSSTNHFEKIQIIPRSSWSDHDASSGVSSACPYNGKSSFFRNVVWLIKKNNAYPTIKRTYNNTNVEDLLILWGIHHPNDATEQTKLYQNSNTYVFVGTSTLNQRSIPEIATRPKVN

**AAN17263.1** HLMSSTNHFEKIQIIPRSSWSDHDASLGVSSACPYNGRSSFFRNVVWLITKNNAYPTIKRTYNNTNVEDLLILWGIHHPNDATEQTKLYQNSNTYVFVGTSTLNQRSIPEIATRPKVN

**AAN17262.1** HLMSSTNHFEKIQIIPRSSWSNHDASSGVSSACPYKGRSSFFRNVVWLINKNNAYPTIKRTYNNTNVEDLLILWGIHHPNDATEQTKLYQNSNTYVFVGTSTLNQRSIPEIATRPKVN

**AAN17261.1** HLMSSTNHFEKIQIIPRSSWSNHDASSGVSSACPYNGGSSFFRNVVWLIKKNNAYPTIKRTYNNTNVEDLLILWGIHHPNDATEQTKLYQNSNTYVFVGTSTLNQRSIPEIATRPKVN

**AAN17260.1** HLMSSTNHFEKIQIIPRSSWSNHNASSGVSSACPYNGGSSFFRNVVWLIKKNNAYPTIKRTYNNTNVEDLLILWGIHHPNDATEQTKLYQNSNTYVFVGTSTLNQRSIPEIATRPKVN

**AAN17259.1** HLMSSTNHFEKIQIIPRSSWSDHDASSGVSSACPYNGRSSFFRNVVWLITKNNAYPTIKRTYNNTNVEDLLILWGIHHPNDAKEQTKLYQNSNTYVFVGTSTLNQRSIPEIATRPKVN

**AAN17258.1** HLMSSTNHFEKIQIIPRSSWSNHNASSGVSSACPYNGRSSFFRNVVWLIKKNNAYPTIKRTYNNTNVEDLLILWGIHHPNDATEQTKLYQNSNTYVFVGTSTLNQRSIPEIATRPKVN

**AAN17257.1** HLMSSTNHFEKIQIIPRSSWSNHNASSGVSSACPYNGRSSFFRNVVWLIKKNNAYPTIKRTYNNTNVEDLLILWGIHHPNDATEQTKLYQNSNTYVFVGTSTLNQRSIPEIATRPKVN

**AAN17256.1** HLMSSTNHFEKIQIIPRSSWSNHDASSGVSSACPYNGRSSFFRNVVWLINKNNAYPTIKRTYNNTNVEDLLILWGIHHPNDATEQTKLYQNSNTYVFVGTSTLNQRSIPEIATRPKVN

**AAN17255.1** HLMSSTNHFEKIQIIPRSSWSDHDASSGVSSACPYNGRSSFFRNVVWLIKKNNAYPTIKRTYNNTNVEDLLILWGIHHPNDATEQTKLYQNSNTYVFVGTSTLNQRSIPEIATRPKVN

**AAN17254.1** HLMSSTNHFEKIQIIPRSSWSNHDASSGVSSACPYNGRSSFFRNVVWLIKKNNAYPTIKRTYNNTNVEDLLILWGIHHPNDATEQTKLYQNSNTYVFVGTSTLNQRSIPEIATRPKVN

**AAG38534.1** HLLSSTNHFEKIRIIPRSSWSNHDASSGVSSACPYNGRSSFFRNVVWLIKKNNAYPTIKRSYNNTNQEDLLILWGIHHPNDAAEQTKLYQNPTTYVSVGTSTLNQRSVPEIATRPKVN

**AAF02306.1** HLLSRINHFEKIQIIPKSSWSNHDASSGVSSACPYLGRSSFFRNVVWLIKKNSAYPTIKRSYNNTNQEDLLVLWGIHHPNDAAEQTKLYQNPTTYISVGTSTLNQRLVPEIATRPKVN

**AAL75847.1** HLLSRINHFEKIQIIPKSSWSNHEASSGVSSACPYQGKSSFFRNVVWLIKKNSAYPTIKRSYNNTNQEDLLVLWGIHHPNDAAEQTKLYQNPTTYISVGTSTLNQRLVPKIATRSKVN

**AAC32101.1** HLLSRINHFEKIQIIPKSSWSNHDASSGVSSACPYLGRSSFFRNVVWLIKKNSAYPTIKRSYNNTNQEDLLVLWGIHHPNDAAEQTKLYQNPTTYISVGTSTLNQRLVPEIATRPEVN

**AAC32099.1** HLLSRISHFEKIQIIPKSSWSNHDASSGVSSACPYLGKSSFFRNVVWLIKKNSTYPTIKRSYNNTNQEDLLVLWGIHHPNDAAEQTKLYQNPTTYISVGTSTLNQRLVPEIATRPKVN

**AAC32098.1** HLLSRINHFEKIQIIPKSSWSNHDASSGVSSACPYLGRSSFFRNVVWLIKKNSAYPTIKRSYNNTNQEDLLVLWGIHHPNDAAEQIKLYQNPTSYISVGTSTLNQRLVPEIATRPKVN

**AAC32088.1** HLLSRINHFEKIQIIPKSSWSNHDASSGVSSACPYLGRSSFFRNVVWLIKKNSAYPTIKRSYNNTNQEDLLVLWGIHHPNDAAEQTKLYQNPTTYISVGTSTLNQRLVPEIATRPKVN

**AAC32078.1** HLLSRINHFEKIQIIPKSSWSNHDASSGVSSACPYLGRSSFFRNVVWLIKKNSTYPTIKRSYNNTNQEDLLVLWGIHHPNDAAEQTKLYQNPTTYISVGTSTLNQRLVPEIATRPKVN

**AAR99628.1** HLLSRINHFEKIQIIPKSSWSDHEASSGVSSACPYQGSSSFFRNVVWLIKKNDAYPTIKRSYNNTNQEDLLVLWGIHHPNDAAEQTRLYQNPTTYISVGTSTLNQRLVPKIATRSKVN

**AAD13568.1** HLLSRINHFEKIQIIPKSSWSNHDASSGVSSACPYLGRSSFFRNVVWLIKKNSAYPTIKRSYNNTNQEDLLVLWGIHHPNDAAEQTKLYQNPTTYISVGTSTLNQRLVPEIATRPKVN

**AAM49555.1** HLLSRINHFEKIQIIPKSSWSNHEASSGVSSACPYNGKSSFFRNVIWLIKKNSAYPTIKRSYNNTNQEDLLILWGIHHPNDAAEQTKLYQNPTTYISVGTSTLNQRLVPKIATRSKVN

**AAL31387.1** HLLSRINHFEKIQIIPKSSWSNHEASSGVSSACPYQGKPSFFRNVVWLIKKNSAYPTIKRSYNNTNQEDLLVLWGIHHPNDAAEQIKLYQNPTTYISVGTSTLNQRLVPKIATRSKVN

**AAL75839.1** HLLSRTNHFEKIQIIPKSSWSNHDASSGVSSACPYHGKSSFFRNVVWLIKKNSAYPTIKRSYNNTNQEDLLVLWGIHHPNDAAEQTKLYQNPTTYISVGTSTLNQRLVPEIATRPKVN

**CAC28131.1** HLLSSTNHLEKIQIIPRSSWSNHDASSGVSSACPYIGRSSFFRNVVWLIKKNNAYPTIKRSYNNTNQEDLLVLWGIHHPNDAAEQTKLYQNPTTYVSVGTSTLNQRSVPEIATRPKVN

**AAA43082.1** HLLSCTKHFEKIRIIPRDSWPNHEASLGVSSACPYNGRSSFFRNVVWLIKKDNAYPTIKRSYNNTNKEDLLILWGIHHPNDAAEQTKLYQNPTTYVSVGTSTLNQRSIPKIATRPKLN

**AAA43083.1** HLLSCTKHFEKIRIIPRDSWPNHEASLGVSSACPYNGRSSFFRNVVWLIKKNNAYPTIKRSYSNTNKEDLLILWGIHHPNDAAEQTKLYQNPTTYVSVGTSTLNQRSIPKIATRPKLN

**AAC58999.1** HLMSSTNHFEKIQIIPRNSWSTHDASSGVSSACPYNGRSSFFRNVVWLIKKNNAYPTIKRTYNNTNVEDLLILWGIHHPNDAAEQTKLYQNSNTYVSVGTSTLNQRSIPEIATRPKVN

**AAR16155.1** HLMSSTNQFEKIQIIPRSSWSNHDASSGVSSACPYNGRSSFFRNVVWLIKKNNAYPTIQRTYXNTXVEDLLILWGIHHPNDAAEQTKLYQNSNTYVSVGTSTLNQRSTPKIATRPKVN

**AAD13574.1** HLLSSTNHFEKIQIIPRSSWSNHDASSGVSSACPYNGRSSFFRNVVWLIKKNNAYPTIKRSYNNTNQEDLLILWGIHHPNDAAEQTKLYQNPTTYVSVGTSTLNQRSVPEIATRPKVN

**AAY57197.1** HLLSSTNHFEKIQIIPRSSWSNHDASSGVSSACSYNGRSSFFRNVVWLIKKNNAYPTIKRSYNNTNQEDLLVLWGIHHPNDAAEQTKLYQNPTTYVSVGTSTLNQRSVPEIATRPKVN

**AAD13568.1** HLLSRINHFEKIQIIPKSSWSNHDASSGVSSACPYLGRSSFFRNVVWLIKKNSAYPTIKRSYNNTNQEDLLVLWGIHHPNDAAEQTKLYQNPTTYISVGTSTLNQRLVPEIATRPKVN

**AAD13575.1** HLMSSTNHFEKIQIIPRSSWSNHDASSGVSSACPYNGRSSFFRNVVWLIKKGNAYPTIKRTYNNTNVEDLLILWGIHHPNDAAEQTNFYQNSNTYVSVGTSTLNQRSIPEIATRPKVN

**AAD13572.1** HLMSSTNHFEKIQIIPRSSWSNHDASSGVSSACPYNGRSSFFRNVVWLIKKNNAYPTIKRTYNNTNMEDLLILWGIHHPNDAAEQTKLYQNSNTYVSVGTSTLNQRSIPEIATRPKVN

**AAD13567.1** HLLSRINHFEKIQIIPKSSWSNHDASSGVSSACPYLGRSSFFRNVVWLIKKNSTYPTIKRSYNNTNQEDLLVLWGIHHPNDAAEQTKLYQNPTTYISVGTSTLNQRLVPEIATRPKVN

**AAD13566.1** HLLSRINHFEKIQIIPKSSWSNHDASSGVSSACPYLGRSSFFRNVVWLIKKNSAYPTIKRSYNNTNQEDLLVLWGIHHPNDAAEQTKLYQNPTTYISVGTSTLNQRLVPEIATRPKVN

**AAD13568.1** HLLSRINHFEKIQIIPKSSWSNHDASSGVSSACPYLGRSSFFRNVVWLIKKNSAYPTIKRSYNNTNQEDLLVLWGIHHPNDAAEQTKLYQNPTTYISVGTSTLNQRLVPEIATRPKVN

**AAA43082.1** HLLSCTKHFEKIRIIPRDSWPNHEASLGVSSACPYNGRSSFFRNVVWLIKKDNAYPTIKRSYNNTNKEDLLILWGIHHPNDAAEQTKLYQNPTTYVSVGTSTLNQRSIPKIATRPKLN

**AAA43083.1** HLLSCTKHFEKIRIIPRDSWPNHEASLGVSSACPYNGRSSFFRNVVWLIKKNNAYPTIKRSYSNTNKEDLLILWGIHHPNDAAEQTKLYQNPTTYVSVGTSTLNQRSIPKIATRPKLN

**AAG01225.1** HLLSRTNHFEKIQIIPKSSWSNHDASSGVSSACPYHGKSSFFRNVVWLTKKNSAYPTIKRSYNNTNQEDLLVLWGIHHPNDAAEQTKLYQNPTTYISVGTSTLNQRLVPEIATRPKVN

**AAG01215.1** HLLSRTNHFEKIQIIPKSSWSNHDASSGVSSACPYHGKSSFFRNVVWLIKKNSAYPTIKRSYNNTNQEDLLVLWGIHHPNDAAEQTKLYQNPTTYISVGTSTLNQRLVPEIATRPKVN

**AAG01205.1** HLLSRTNHFEKIQIIPKSSWSNHDASSGVSSACPYHGKSSFFRNVVWLIKKNSAYPTIKRSYNNTNQEDLLVLWGIHHPNDAAEQTKLYQNPTTYISVGTSTLNQRLVPEIATRPKVN

**AAG01195.1** HLLSRTNHFEKIQIIPKSSWSNHDASSGVSSACPYHGKSSFFRNVVWLIKKNSAYPTIKRSYNNTNQEDLLVLWGIHHPNDAAEQTKLYQNPTTYISVGTSTLNQRLVPEIATRPKVN

**AAD51927.1** HLLSRTNHFEKIQIIPKSSWSNHDASSGVSSACPYHGRSSFFRNVVWLIKKNSAYPTIKRSYNNTNQEDLLVLWGIHHPNDAAEQTKLYQNPTTYISVGTSTLNQRLVPEIATRPKVN

**AAF04720.1** HLMSSTNHFEKIQIIPRSSWSNHDASSGVSSACPYNGRSSFFRNVVWLIKKNNAYPTIKRTYNNTNVEDLLILWGIHHPNDATEQTKLYQNSNTYVSVGTSTLNQRSIPEIATRPKVN

**AAF04719.1** HLMSSTNHFEKIQIIPRSSWSNHDASSGVSSACPYNGRSSFFRNVVWLIKKNNAYPTIKRTYNNTNVEDLLILWGIHHPNDATEQTKLYQNSNTYVFVGTSTLNQRSIPEIATRPKVN

HA1 <--||--> HA2

**AAT65209.1 GQSGRIEFFWTILKPNDAINFESNGNFIAPEYAYKIVKKGDSAIMKSELEYGNCNTKCQTPMGAINSSMPFHNIHPLTIGESPKYVKSSRLVLATGLRNTPHRERIRKKRGLFGAIAG**

**AAA43083.1** GQSGRMEFFWTILKPSDTINFESNGNFIAPEYAYKIVKKGDSAIMKSGLEYGNCNTKCQTPIGAINSSMPFHNIHPLTIGECPKYVKSDRLVLATGLRNTPQRK--RKKRGLFGAMAG

**AAR16155.1** GQSGRMEFFWTILKPSDTINFESNGNFIAPEYAYKIVKKGDSAIMKSGLEYGNCNTKCQTPIGAINSSMPFHNIHPLTIGECPKYVKSDRLVLATGLRNTPQRK--RKKRGLFGAIAG

**CAF21870.1** GQSGRMDFFWTILKPNDAINFESNGNFIAPEYAYKIVKKGDSAIMRSGLEYGNCNTKCQTPMGAINSSMPFHNIHPLTIGECPKYVKSDRLVLATGLRNVPQRE----TRGLFGAIAG

**ABF58847.1** GQSGRMEFFWTILKPNDAINFESNGNFIAPEYAYKIVKKGDSTIMKSELEYGNCNTKCQTPMGAINSSMPFHNIHPLTIGECPKYVKSNRLVLATGLRNSPQRERRRKKRGLFGAIAG

**AAC58998.1** GQSGRIEFFWTILRPNDAISFESNGNFIAPEYAYKIVKKGDSAIMRSELEYGNCDTKCQTPVGAINSSMPFHNVHPLTIGECPKYVKSDKLVLATGLRNVPQRE----TRGLFGAIAG

**AAC58997.1** GQSGRVEFFWTILRPNDPISFESNGNFIAPEYAYKIVKKGDSAIMKSELEYGNCDTKCQTPVGAINSSMPFHNVHPLTIGECPKYVKSDKLVLATGPRNVPQRE----TRGLFGAIAG

**AAC58996.1** GQSGRMEFFWTILRPNDAISFESNGNFIAPEYAYKIVKKGDSAIMKSELEYGNCNTKCQTPVGAINSSMPFHNVHPFTIGECPKYVKSDKLVLATGPRNVPQRE----TRGLFGAIAG

**AAC58994.1** GQSGRMEFFWTILKPNDAISFESNGNFIAPEYAYKIVKKGDSAIMRSELEYGNCDTKCQTPVGAINSSMPFHNVHPLTIGECPKYVKSDKLVLATGLRNVPQRE----TRGLFGAIAG

**AAC58991.1** GQSGRMEFFWTILSPNDAISFESNGNFIAPEYAYKIVKKGDSAIMSSELEYGNCNAKCQTPVGTINSSMPFHNVHPLTIGECPKYVKSDKLVLATGLRNVPQRE----TRGLFGAIAG

**AAC34263.1** GQSGRMEFFWTILRPNDSINFESTGNFIAPEYAYKLIKKGDSAIMKSELNYGNCDTKCQTPAGAINSRMPFHNVHPFTIGECPKYVKSKKLVLATGLRNVPQRKRKRKTRGLFGAIAG

**BAE48684.1** GQSGRIEFSWTILKPNDAIISESNGNFIAPEYAYKIAKKGDSAIMKSGLEYGNCNTKCQTPMGAINSSMPFHNIHPLTIGECPKYVKSDRLVLATGLRNVPQRE----TRGLFGAIAG

**AAV91149.1** GQSGRMEFFWTILKPNDAINFESNGNFIAPEYAYKIVKKGDSAIMKSELEYGNCNTKCQTPMGAINSSMPFHNIHPLTIGECPKYVKSNRLVLATGLRNSPQGERRRKKRGLFGAIAG

**AAC58999.1** GQSGRMEFFWTILRPNDAISFESNGNFIAPEYAYKIVKKGDSAIMKSELEYGNCDTKCQTPVGAINSSMPFHNVHPLTIGECPKYVKSDKLVLATGLRNVPQRE----TRGLFGAIAG

**AAP72011.1** GQSGRMEFFWTILRPNDAISFESNGNFIAPEYAYKIVKRGDSAIMKSELEYGNCDTKCQTPMGAINSSMPFHNVHPLTIGECPKYVKSDKLVLATGLRNVPQRE----TRGLFGAIAG

**AAP72010.1** GQSGRMEFFWTILRPNDTISFESNGNFIAPEYAYKIAKKGKSAIMKSELEYGNCDTKCQTPVGAINSSMPFHNVHPLTIGECPKYVKSNKLVLATGPRNVPQRE----KRGLFGAIAG

**AAP72009.1** GQSGRMEFFWTILRSNDTISFESNGNFIAPEYAYKIVKKGDSAIMRSELEYGNCDTKCQTPVGAINSSMPFHNIHPLTIGECPKYVKSDKLVLATGLRNVPQRE----TRGLFGAIAG

**AAP72008.1** GQSGRMEFFWTILRPNDAISFESNGNFIAPEYAYKIVKKGDSAIMRSELEYGNCDTKCQTPVGAINSSMPFHNVHPLTIGECPKYVKSDKLVLATGPRNVPQRE----TRGLFGAIAG

**AAP72007.1** GQSGRMEFFWTILRPNDAISFESNGNFIAPEYAYKIVKRGDSAIMKSELEYGNCDTKCQTPMGAINSSMPFHNVHPLTIGECPKYVKSDKLVLATGLRNVPQRE----TRGLFGAIAG

**AAP72011.1** GQSGRMEFFWTILRPNDAISFESNGNFIAPEYAYKIVKRGDSAIMKSELEYGNCDTKCQTPMGAINSSMPFHNVHPLTIGECPKYVKSNKLVLATGLRNVPQRE----TRGLFGAIAG

**AAP72005.1** GQSGRMEFFWTILRPNDAISFESNGNFIAPEYAYKIVKRGDSAIMKSELEYGNCDTKCQTPMGAINSSMPFHNVHPLTIGECPKYVKSDKLVLATGLRNVPQRE----TRGLFGAIAG

**AAP72004.1** GQSGRMEFFWTILRPNDAISFESNGNFIAPEYAYKIVKKGDSAIMRSELEYGNCDTKCQTPVGAINSSMPFHNVHPLTIGECPKYVKSNKLVLATGLRNVPQRE----TRGLFGAIAG

**AAP72003.1** GQSGRMEFFWTILRPNDAISFESNGNFIAPEYAYKIVKKGDSAIMRSELEYGNCDTKCQTPVGAINSSMPFHNVHPLTIGECPKYVKSDKLVLATGLRNVPQRE----TRGLFGAIAG

**AAP72002.1** GQSGRMEFFWTVLRPNDAISFESNGNFIAPEYAYKIVKKGDSAIMRSELEYGNCDTKCQTPVGAINSSMPFHNVHPLTIGECPKYVKSDKLVLATGLRNVPQRE----TRGLFGAIAG

**AAP72001.1** GQSGRMEFFWTILRPNDAISFESNGNFIAPEYAYKIVKRGDSAIMKSELEYGNCDTKCQTPMGAINSSMPFHNVHPLTIGECPKYVKSDKLVLATGLRNVPQRE----TRGLFGAIAG

**AAP72000.1** GQSGRMEFFWTILRPNDAISFESNGNFIAPEYAYKIVKKGDSAIMRSELEYGNCDTKCQTPVGAINSSMPFHNVHPLTIGECPKYVKSDKLVLATGLRNVPQGE----TRGLFGAIAG

**AAP71998.1** GQSGRMEFFWTILRPNDTISFESNGNFIAPEYAYKIVKKGDSAIMKSELDYGNCDTKCQTPVGAINSSMPFHNVHPLTIGKCPKYVKSDKLVLATGLRNVPQRE----TRGLFGAIAG

**AAP71997.1** GQSGRMEFFWTILRPNDTISFESNGNFIAPEYAYKIVKKGDSAIMKNELDYGNCDTKCQTPVGAISSSMPFHNVHPLTIGECPKYVKSDKLVLATGLRNVPQRE----TRGLFGAIAG

**AAP71996.1** GQSGRMEFFWTILRPNDTISFESNGNFIAPEYAYKIVKKGDSAIMKSELDYGNCDTKCQTPVGAINSSMPFHNVHPLTIGECPKYVKSDKLVLATGLRNVPQRE----TRGLFGAIAG

**AAP71995.1** GQSGRMEFFWTILRPNDTISFESNGNFIAPEYAYKIVKKGDSAIMKSELDYGNCDTKCQTPVGAINSSMPFHNVHPLTIGECPKYVKSDKLVLATGLRNVPQRE----TRGLFGAIAG

**AAN17270.1** GQSGRMEFFWTILRPNDAISFESNGNFIAPEYAYKIVKKGDSTIMKSELGYGNCNTKCQTPVGAINSSMPFHNVHPLTIGECPKYVKSDKLVLATGPRNVPQRE----TRGLFGAIAG

**AAN17269.1** GQSGRMEFFWTILRPNDAISFESNGNFIAPEYAYKIVKKGDSTIMKSELGYGNCNTKCQTPVGAINSSMPFHNVHPLTIGECPKYVKSDKLVLATGPRNVPQRE----TRGLFGAIAG

**AAN17256.1** GQSGRMEFFWTILRPNDAISFESNGNFIAPEYAYKIVKKGDSTIMKSELGYGNCNTKCQTPVGAINSSMPFHNVHPLTIGECPKYVKSDKLVLATGPRNVPQRE----TRGLFGAIAG

**AAN17267.1** GQSGRMEFFWTILRPNDAISFESNGNFIAPEYAYKIVKKGDSTIMKSELGYGNCNTKCQTPVGAINSSMPFHNVHPLTIGECPKYVKSDKLVLATGPRNVPQRE----TRGLFGAIAG

**AAN17266.1** GQSGRMEFFWTILRPNDAISFESNGNFIAPEYAYKIVKKGDSTIMKSELGYGNCNTKCQTPVGAINSSMPFHNVHPLTIGECPKYVKSDKLVLATGPRNVPQRE----TRGLFGAIAG

**AAN17265.1** GQSGRMEFFWTILRPNDAISFESNGNFIAPEYAYKIVKKGDSTIMKSELGYGNCNTKCQTPVGAINSSMPFHNVHPLTIGECPKYVKSDKLVLATGPRNVPQRE----TRGLFGAIAG

**AAN17264.1** GQSGRMEFFWTILRPNDAISFESNGNFIAPEYAYKIVKKGDSTIMKSELGYGNCNTKCQTPVGAINSSMPFHNVHPLTIGECPKYVKSDKLVLATGPRNVPQRE----TRGLFGAIAG

**AAN17263.1** GQSGRMEFFWTILRPNDAISFESNGNFIAPEYAYKIVKKGDSTIMKSELGYGNCNTKCQTPVGAINSSMPFHNVHPLTIGECPKYVKSDKLVLATGPRNVPQRE----TRGLFGAIAG

**AAN17262.1** GQSGRMEFFWTILRPNDAISFESNGNFIAPEYAYKIVKKGDSTIMKSELGYGNCNTKCQTPVGAINSSMPFHNVHPLTIGECPKYVKSDKLVLATGPRNVPQRE----TRGLFGAIAG

**AAN17261.1** GQSGRMEFFWTILRPNDAISFESNGNFIAPEYAYKIVKKGDSTIMKSELGYGNCNTKCQTPVGAINSSMPFHNVHPLTIGECPKYVKSDKLVLATGPRNVPQRE----TRGLFGAIAG

**AAN17260.1** GQSGRMEFFWTILRPNDAISFESNGNFIAPEYAYKIVKKGDSTIMKSELGYGNCNTKCQTPVGAINSSMPFHNVHPLTIGECPKYVKSDKLVLATGPRNVPQRE----TRGLFGAIAG

**AAN17259.1** GQSGRMEFFWTILRPNDAISFESNGNFIAPEYAYKIVKKGDSTIMKSELGYGNCNTKCQTPVGAINSSMPFHNVHPLTIGECPKYVKSDKLVLATGPRNVPQRE----TRGLFGAIAG

**AAN17258.1** GQSGRMEFFWTILRPNDAISFESNGNFIAPEYAYKIVKKGDSTIMKSELGYGNCNTKCQTPVGAINSSMPFHNVHPLTIGECPKYVKSDKLVLATGPRNVPQRE----TRGLFGAIAG

**AAN17257.1** GQSGRMEFFWTILRPNDAISFESNGNFIAPEYAYKIVKKGDSTIMKSELGYGNCNTKCQTPVGAINSSMPFHNVHPLTIGECPKYVKSDKLVLATGPRNVPQRE----TRGLFGAIAG

**AAN17256.1** GQSGRMEFFWTILRPNDAISFESNGNFIAPEYAYKIVKKGDSTIMKSELGYGNCNTKCQTPVGAINSSMPFHNVHPLTIGECPKYVKSDKLVLATGPRNVPQRE----TRGLFGAIAG

**AAN17255.1** GQSGRMEFFWTILRPNDAISFESNGNFIAPEYAYKIVKKGDSTIMKSELGYGNCNTKCQTPVGAINSSMPFHNVHPLTIGECPKYVKSDKLVLATGPRNVPQRE----TRGLFGAIAG

**AAN17254.1** GQSGRMEFFWTILRPNDAISFESNGNFIAPEYAYKIVKKGDSTIMKSELGYGNCNTKCQTPVGAINSSMPFHNVHPLTIGECPKYVKSDKLVLATGPRNVPQRE----TRGLFGAIAG

**AAG38534.1** GQSGRMEFFWTILKPNDAINFESNGNFIAPEYAYKIVKKGGSAIMKSGLEYGNCNTKCQTPMGAINSSMPFHNIHPLTIGECPKYVKSGRLVLATGLRNVPQRE----TRGLFGAIAG

**AAF02306.1** GQSGRMEFFWTILKPNDAINFESNGNFIAPEYAYKIVKKGDSTIMKSELEYGNCNTKCQTPMGAINSSMPFHNIHPLTIGECPKYVKSNRLVLATGLRNTPQRERRRKKRGLFGAIAG

**AAL75847.1** GQSGRMEFFWTILKPNDAINFESNGNFIAPEYAYKIVKKGDSAIMKSELEYGNCNTKCQTPLGAINSSMPFHNIHPLTIGECPKYVKSNRLVLATGLRNTPQRERRRKKRGLFGAIAG

**AAC32101.1** GQSGRMEFFWTILKPNDAINFESNGNFIAPEYAYKIVKKGDSTIMKSELEYGNCNTKCQTPMGAINSSMPFHNIHPLTIGECPKYVKSNRLVLATGLRNTPQRERRRKKRGLFGAIAG

**AAC32099.1** GQSGRIEFFWTILKPNDAINFESNGNFIAPEYAYKIVKKGDSTIMKSELEYGNCNTKCQTPMGAINSSMPFHNIHPLTIGECPKYVKSNRLVLATGLRNAPQRERRRKKRGLFGAIAG

**AAC32098.1** GQSGRMEFFWTILKPNDAINFESNGNFIAPEYAYKIVKKGDSTIMKSELEYGNCNTKCQTPMGAINSSMPFHNIHPLTIGECPKYVKSNRLVLATGLRNTPQRERRRKKRGLFGAIAG

**AAC32088.1** GQSGRMEFFWTILKPNDAINFESNGNFIAPEYAYKIVKKGDSTIMKSELEYGNCNTKCQTPMGAINSSMPFHNIHPLTIGECPKYVKSNRLVLATGLRNTPQRERRRKKRGLFGAIAG

**AAC32078.1** GQSGRMEFFWTILKPNDAINFESNGNFIAPEYAYKIVKKGDSTIMKSELEYGNCNTKCQTPMGAINSSMPFHNIHPLTIGECPKYVKSNRLVLATGLRNTPQRERRRKKRGLFGAIAG

**AAR99628.1** GQSGRMDFFWTILKPNDAINFESNGNFIAPEYAYKIVKKGDSAIMKSEVEYGNCNTKCQTPMGAINSSMPFHNIHPLTIGECPKYVKSNKLVLATGLRNSPQRERRRK-RGLFGAIAG

**AAD13568.1** GQSGRMEFFWTILKPNDAINFESNGNFIAPEYAYKIVKKGDSTIMKSELEYGNCNTKCQTPMGAINSSMPFHNIHPLTIGECPKYVKSNRLVLATGLRNTPQRERRRKKRGLFGAIAG

**AAM49555.1** GQSGRMEFFWTILKPNDTINFESNGNFIAPEYAYKIVKKGDSAIMKSELEYGNCNTKCQTPMGAINSSMPFHNIHPLTIGECPKYVKSNRLVLATGLRNTPQRERRRKKRGLFGAIAG

**AAL31387.1** GQSGRMEFFWTILKPNDAINFESNGNFIAPEYAYKIVKKGDSAIMKSELEYGNCNTKCQTPMGAINSSMPFHNIHPLTIGECPKYVKSNRLVLATGLRNTPQRERRRKKRGLFGAIAG

**AAL75839.1** GQSGRMEFFWTILKPNDAINFESNGNFIAPEYAYKIVKKGDSAIMKSELEYGNCNTKCQTPMGAINSSMPFHNIHPLTIGECPKYVKSNRLVLATGLRNTPQRERRRKKRGLFGAIAG

**CAC28131.1** GQSGRMEFFWTILKPNDVINFESNGNFIAPEYAYKIVKKGDSAIMKSGLEYGNCNTKCQTPMGAINSSMPFHNIHPLTIGECPRYVKSDRLVLATGLRNVPQRRRKK--RGLFGAIAG

**AAA43082.1** GQSGRMEFFWTILKPSDTINFESNGNFIAPEYAYKIVKKGDSAIMKSGLEYGNCNTKCQTPIGAINSSMPFHNIHPLTIGECPKYVKSDRLVLATGLRNTPQRKRKK--RGLFGAIAG

**AAA43083.1** GQSGRMEFFWTILKPSDTINFESNGNFIAPEYAYKIVKKGDSAIMKSGLEYGNCNTKCQTPIGAINSSMPFHNIHPLTIGECPKYVKSDRLVLATGLRNTPQRKRKK--RGLFGAMAG

**AAC58999.1** GQSGRMEFFWTILRPNDAISFESNGNFIAPEYAYKIVKKGDSAIMKSELEYGNCDTKCQTPVGAINSSMPFHNVHPLTIGECPKYVKSDKLVLATGLRNVPQRE----TRGLFGAIAG

**AAR16155.1** GQSGRIEFFWTILRPNDSISFESNGNFIAPEYAYKIVKKGDSAIIKSELEYGNCDTKCQTPVGAINSSMPFHNVHPLTIGECPKYVKSDKLVLATGPRNVPQRKRK--TRGLFGAIAG

**AAD13574.1** GQSGRMEFFWTILKPNDAINFESNGNFIAPEYAYKIVKKGDSAIMKSGLEYGNCNTKCQTPMGAINSSMPFHNIHPLTIGECPKYVKSDRLVLATGLRNVPQRET----RGLFGAIAG

**AAY57197.1** GQSGRMEFFWTILKPNDAINFESNGNFIAPEYAYKIVKKGDSAIMKSGLEYGNCNTKCQTPMGAINSSMPFHNIHPLTIGECPKYVKSDRLVLATGLRNVPQRET----RGLFGAIAG

**AAD13568.1** GQSGRMEFFWTILKPNDAINFESNGNFIAPEYAYKIVKKGDSTIMKSELEYGNCNTKCQTPMGAINSSMPFHNIHPLTIGECPKYVKSNRLVLATGLRNTPQRERRRKKRGLFGAIAG

**AAD13575.1** GQSGRMEFFWTILKPNDAISFESNGNFIAPEYAYKIVKKGDSAIMKSELKYGNCDTKCQTPVGAINSSMPFHNVHPLTIGECPKYVKSEKLVLATGLRNVPQRET----KGLFGAIAG

**AAD13572.1** GQSGRMEFFWTILRPNDAISFESNGNFIAPEYAYKIVKKGDSAMMKSELEYGNCNTKCQTPVGAINSSMPFHNVHPLTIGECPKYVKSDKLVLATGLRNVPQRET----RGLFGAIAG

**AAD13567.1** GQSGRMEFFWTILKPNDAINFESNGNFIAPEYAYKIVKKGDSTIMKSELEYGNCNTKCQTPMGAINSSMPFHNIHPLTIGECPKYVKSNRLVLATGLRNTPQRERRRKKRGLFGAIAG

**AAD13566.1** GQSGRMEFFWTILKPNDAINFESNGNFIAPEYAYKIVKKGDSTIMKSELEYGNCNTKCQTPMGAINSSMPFHNIHPLTIGECPKYVKSNRLVLATGLRNTPQRERRRKKRGLFGAIAG

**AAD13568.1** GQSGRMEFFWTILKPNDAINFESNGNFIAPEYAYKIVKKGDSTIMKSELEYGNCNTKCQTPMGAINSSMPFHNIHPLTIGECPKYVKSNRLVLATGLRNTPQRERRRKKRGLFGAIAG

**AAA43082.1** GQSGRMEFFWTILKPSDTINFESNGNFIAPEYAYKIVKKGDSAIMKSGLEYGNCNTKCQTPIGAINSSMPFHNIHPLTIGECPKYVKSDRLVLATGLRNTPQRKRKK--RGLFGAIAG

**AAA43083.1** GQSGRMEFFWTILKPSDTINFESNGNFIAPEYAYKIVKKGDSAIMKSGLEYGNCNTKCQTPIGAINSSMPFHNIHPLTIGECPKYVKSDRLVLATGLRNTPQRKRKK--RGLFGAMAG

**AAG01225.1** GQSGRMEFFWTILKPNDAINFESNGNFIAPEYAYKIVKKGDSAIMKSELEYGNCNTKCQTPMGAINSSMPFHNIHPLTIGECPKYVKSNRLVLATGLRNTPQRERRRKKRGLFGAIAG

**AAG01215.1** GQSGRMEFFWTILKPNDAINFESNGNFIAPEYAYKIVKKGDSAIMKSELEYGNCNTKCQTPMGAINSSMPFHNIHPLTIGECPKYVESNRLVLATGLRNTPQRERRRKKRGLFGAIAG

**AAG01205.1** GQSGRMEFFWTILKPNDAINFESNGNFIAPEYAYKIVKKGDSAIMKSELEYGNCNTKCQTPMGAINSSMPFHNIHPLTIGECPKYVKSNRLVLATGLRNTPQRERRRKKRGLFGAIAG

**AAG01195.1** GQSGRMEFFWTILKPNDAINFESNGNFIAPEYAYKIVKKGDSAIMKSELEYGNCNTKCQTPMGAINSSMPFHNIHPLTIGECPKYVKSNRLVLATGLRNTPQRERRRKKRGLFGAIAG

**AAD51927.1** GQSGRMEFFWTILKPNDAINFESNGNFIAPEYAYKIVKKGDSAIMKSELEYGNCNTKCQTPMGAINSSMPFHNIHPLTIGECPKYVKSNRLVLATGLRNTPQRERRRKKRGLFGAIAG

**AAF04720.1** GQSGRMEFFWTILRPNDAISFESNGNFIAPEYAYKIVKKGDSAIMKSELEYGNCNTKCQTPVGAINSSMPFHNVHPLTIGECPKYVKSDKLVLATGLRNVPQRETR----GLFGAIAG

**AAF04719.1** GQSGRMEFFWTILRPNDAISFESNGNFIAPEYAYKIVKKGDSAIMKSELGYGNCNTKCQTPVGAINSSMPFHNVHPLTIGECPKYVKSDKLVLATGPRNVPQRETR----GLFGAIAG

**AAT65209.1 FIEGGWQGMVDGWYGYHHSNEQGSGYAADKESTQKAIDGVTNKVNSIIDKMNTQFVAVGREFNNLERRIENLNKKMEDGFLDVWTYNAELLVLMENERTLDFHDSNVRNLYDKVRLQLR**

**AAA43083.1** FIEGGWQGMVDGWYGYHHSNEQGSGYAADKESTQKAIDGITNKVNSIIDKMNTQFEAVGKEFNNLERRIENLNKILEDGFLDVWTYNAELLVLMENERTLDFHDANVKSLYDKVRLQLK

**AAR16155.1** FIEGGWQGMVDGWYGYHHSNEQGSGYAADKESTQKAIDGITNKVNSIIDKMNTQFEAVGKEFNNLERRIENLNKMLEDGFLDVWTYNAELLVLMENERTLDFHEANVKSLYDKVRLQLK

**CAF21870.1** FIEGGWQGMVDGWYGYHHSNEQGSGYAADKESTQKAIDGITNKVNSIIDKMNTQFEAVGKEFNNLEGRIENLNKKMEDGFLDVWTYNAELLVLMENERTLDFHDSNVKNLYDKVRLQLR

**ABF58847.1** FIEGGWQGMVDGWYGYHHSNEQGSGYAADKESTQKAIDGVTNKVNSIIDKMNTQFEAVGREFNHLERRIENLNKKMEDGFLDVWTYNAELLVLMENERTLDFHDSNVKNLYDKVRLQLR

**AAC58998.1** FIEGGWQGMVDGWYGYHHSNEQGSGYAADKESTQKAIDGITNKVNSIIDKMNTQFEAVGKEFNNLERRIENLNKKMEDGFLDVWTYNAELLVLMENERTLDFHDSYVKNLYDKVRLQLR

**AAC58997.1** FIEGGWQGMVDGWYGYHHSYEQGSGYAADKESTQKAIDGITNEVNSIIDRMNTQFEAVGKEFNSLERRIENLNKKMEDGFLDVWTYNAELLVLMETERTLDFHDSNVKNLYDKVRLQLR

**AAC58996.1** FIEGGWQGMVDGWYGYHHSNEQGSGYAADKESTQKAIDGITNKVNSIIDKMNTQFEAVGKEFNNLERRIENLNKKMEDGFLDVWTYNAELLVLMENERTLDFHDSNVRNLYDKVRLQLR

**AAC58994.1** FIEGGWQGMVDGWYGYHHSNEQGSGYAADKESTQKAIDGITNKVNSIIDKMNTQFEAVGKEFNNLERRIENLNKKMEDGFLDVWTYNAELLVLMENERTLDFHDSNVKNLYDKIRLQLR

**AAC58991.1** FIEGGWQGMVDGWYGYHHSNEQGSGYAADKESTQKAIDGITNKVNSVINKMNTQFEAVGKEFNSLERRIENLNKKMEDGFLDVWTYNAELLVLMENERTLDFHDPNVRNLYDKVRLQLR

**AAC34263.1** FIEGGWQGMVDGWYGYHHSNEQGSGYGEDNESTQKAIDGITNKVNSIIDKMNTQFEAVGKEFNNLERRIENLNKKMEDGFIDVWTYNAELLVLMENERTLDLHDSNVKKLYDRVRLQLR

**BAE48684.1** FIEGGWQGMVDGWYGYHHSNEQGSGYAADKESTQKAIDGITNKVNSIIDKMNTQFEAVGKEFNNLERRIENLNKKMEDGFLDVWTYNAELLVLMENERTLDFHDSNVKNLYDKVRLQLR

**AAV91149.1** FIEGGWQGMVDGWYGCHHSNEQGSGYAADKESTQKAIDGVTNKVNSIIDKMNTQFEAVGREFNNLERRIENLNKKMEDGFLDVWTYNAELLVLMENERTLDFHDSNVKNLYDKVRLQLR

**AAC58999.1** FIEGGWQGMVDGWYGYHHSNEQGSGYAADKESTQKAIDGITNKVNSIIDKMNTQFEAVGKEFNNLERRIENLNKKMEDGFLDVWTYNAELLVLMENERTLDFHDSNVKNLYDKVRLQLR

**AAP72011.1** FIEGGWQGMVDGWYGYHHSNEQGSGYAADKESTQKAIDGITNKVNSIIDKMNTQFEAVGKEFSNLERRIENLNKKMEDGFLDVWTYNAELLVLMENERTLDFHDSNVKNLYDKVRLQLR

**AAP72010.1** FIEGGWQGMVDGWYGYHHSNEQGSGYAADRKSTQKAIDGITNKVNSIIDKMNTQFEAVGKEFNNLERRIEDLNKKMEDGFLDVWTYNAELLVLMENERTLDFHDSNVKNLYDKVRLQLR

**AAP72009.1** FIEGGWQGMVDGWYGYHHSNEQGSGYAADKESTQKAIDGITNKVNSIIEKMNTQFEAVGKEFNNLERRIENLNKKMEDGFLDVWTYNAELLVLMENERTLDFHDSNVKNLYDKVRLQLR

**AAP72008.1** FIEGGWQGMVDGWYGYHHSNEQGSGYAADKESTQKAIDGITNKVNSIIDKMNTQFEAVGKEFNNLERRIENLNKKMEDGFLDVWTYNAELLVLMENERTLDFHDSNVKNLYDKVRLQLR

**AAP72007.1** FIEGGWQGMVDGWYGYHHSNEQGSGYAADKESTQKAIDGITNKVNSIIDKMNTQFEAVGKEFSNLERRIENLNKKMEDGFLDVWTYNAELLVLMENERTLDFHDSNVKNLYDKVRLQLR

**AAP72011.1** FIEGGWQGMVDGWYGYHHSNEQGSGYAADKESTQKAIDGITNKVNSIIDKMNTQFEAVGKEFSNLERRIENLNKKMEDGFLNVWTYNAELLVLMENERTLDFHDSNVKNLYDKVRLQLR

**AAP72005.1** FIEGGWQGLVDGWYGYHHSNEQGSGYAADKESTQKAIDGITNKVNSIIDKMNTQFEAVGKEFSNLERRIENLNKKMEDGFLNVWTYNAELLVLMENERTLDFHDSNVKNLYDKVRLQLR

**AAP72004.1** FIEGGWQGMVDGWYGYHHSNEQGSGYAADKESTQKAIDGITNKVNSIIDKMNTQFEAVGKEFNNLERRIENLNKKMEDGFLDVWTYNAELLVLMENERTLDFHDSNVKNLYDKVRLQLR

**AAP72003.1** FIEGGWQGMVDGWYGYHHSNEQGSGYAADKESTQKATDGITNKVNSIIDKMNIQFEAVGKGFNNLERRIENLNKKMEDGFLDVWTYNAELLVLMENERTLDFHDSNVKNLYNKVRLQLR

**AAP72002.1** FIEGGWQGMVDGWYGYHHSNEQGSGYAADKESTQKAIDGITNKVNSIIDKMNTQFEAVGKEFNNLERRIENLNKKMEDGFLDVWTYNAELLVLMENERTLDFPDSNVKNLYDKVRLQLR

**AAP72001.1** FIEGGWQGMVDGWYGYHHSNEQGSGYAADKESTQKAIDGITNKVNSIIDKMNTQFEAVGKEFSNLERRIENLNKKMEDGFLDVWTYNAELLVLMENERTLDFHDSNVKNLYDKVRLQLR

**AAP72000.1** FIEGGWQGMVDGWYGYHHSNEQGSGYAADKESTQKAIDGITNKVNSIIDKMNTQFEAVGKEFNNLERRIENLNKKMEDGFLDVWTYNAELLVLMENERTLDFHDSNVKNLYDKVRLQLR

**AAP71998.1** FIEGGWQGMVDGWYGYHHSNEQGSGYAADKESTQKAIDGVTNKVNSIIDKMNTQFEAVGKEFNNLERRIENLNKKMEDGFLDVWTYNAELLVLMENERTLDFHDSNVKNLYDKVRLQLR

**AAP71997.1** FIEGGWQGMVDGWYGYHHSNEQGSGYAADKESTQKAIDGVTNKVNSIIDKMNTQFEAVGKEFNNLERRIENLNKKMEDGFLEVWTYNAELLVLMENERTLDFHDSNVKNLYDKVRLQLR

**AAP71996.1** FIEGGWQGMVDGWYGYQHSNEQGSGYAADKESTQKAIDGVTNKVNSIIDKMNTRFEAVGKEFNNLERRIENLNKKMEDGFLDVWTYNAELLVLMENERTLDFHDSNVKNLYDKVRLQLR

**AAP71995.1** FIEGGWQGMVDGWYGYHHSNEQGSGYAADKESTQKAIDGVTNKVNSIIDKMNTRFEAVGKEFNNLERRIENLNKKMEDGFLDVWTYNAELLVLMENERTLDFHDSNVKNLYDKVRLQLR

**AAN17270.1** FIEGGWQGMVDGWYGYHHSNEQGSGYAADKESTQKAIDGITNKVNSIIDKMNTQFEVVGKEFNNLERRIENLNKKMEDGFLDVWTYNAELLVLMENERTLDFHDSNVRNLYDKVRLQLR

**AAN17269.1** FIEGGWQGMVDGWYGYHHSNEQGSGYAADKESTQKAIDGITNKVNSIIDKMNTQFEVVGKEFNNLERRIENLNKKMEDGFLDVWTYNAELLVLMENERTLDFHDSNVRNLYDKVRLQLR

**AAN17256.1** FIEGGWQGMVDGWYGYHHSNEQGSGYAADKESTQKAIDGITNKVNSIIDKMNTQFEVVGKEFNNLERRIENLNKKMEDGFLDVWTYNAELLVLMENERTLDFHDSNVRNLYDKVRLQLR

**AAN17267.1** FIEGGWQGMVDGWYGYHHSNEQGSGYAADKESTQKAIDGITNKVNSIIDKMNTQFEVVGKEFNNLERRIENLNKKMEDGFLDVWTYNAELLVLMENERTLDFHDSNVRNLYDKVRLQLR

**AAN17266.1** FIEGGWQGMVDGWYGYHHSNEQGSGYAADKESTQKAIDGITNKVNSIIDKMNTQFEVVGKEFNNLERRIENLNKKMEDGFLDVWTYNAELLVLMENERTLDFHDSNVRNLYDKVRLQLR

**AAN17265.1** FIEGGWQGMVDGWYGYHHSNEQGSGYAADKESTQKAIDGITNKVNSIIDKMNTQFEVVGKEFNNLERRIENLNKKMEDGFLDVWTYNAELLVLMENERTLDFHDSNVRNLYDKVRLQLR

**AAN17264.1** FIEGGWQGMVDGWYGYHHSNEQGSGYAADKESTQKAIDGITNKVNSIIDKMNTQFEVVGKEFNNLERRIENLNKKMEDGFLDVWTYNAELLVLMENERTLDFHDSNVKNLYDKVRLQLR

**AAN17263.1** FIEGGWQGMVDGWYGYHHSNEQGSGYAADKESTQKAIDGITNKVNSIIDKMNTQFEVVGKEFNNLERRIENLNKKMEDGFLDVWTYNAELLVLMENERTLDFHDSNVRNLYDKVRLQLR

**AAN17262.1** FIEGGWQGMVDGWYGYHHSNEQGSGYAADKESTQKAIDGITNKVNSIIDKMNTQFEVVGKEFNNLERRIENLNKKMEDGFLDVWTYNAELLVLMENERTLDFHDSNVRNLYDKVRLQLR

**AAN17261.1** FIEGGWQGMVDGWYGYHHSNEQGSGYAADKESTQKAIDGITNKVNSIIDKMNTQFEVVGKEFNNLERRIENLNKKMEDGFLDVWTYNAELLVLMENERTLDFHDSNVRNLYDKVRLQLR

**AAN17260.1** FIEGGWQGMVDGWYGYHHSNEQGSGYAADKESTQKAIDGITNKVNSIIDKMNTQFEVVGKEFNNLERRIENLNKKMEDGFLDVWTYNAELLVLMENERTLDFHDSNVRNLYDKVRLQLR

**AAN17259.1** FIEGGWQGMVDGWYGYHHSNEQGSGYAADKESTQKAIDGITNKVNSIIDKMNTQFEVVGKEFNNLERRIENLNKKMEDGFLDVWTYNAELLVLMENERTLDFHDSNVRNLYDKVRLQLR

**AAN17258.1** FIEGGWQGMVDGWYGYHHSNEQGSGYAADKESTQKAIDGITNKVNSIIDKMNTQFEVVGKEFNNLERRIENLNRKMEDGFLDVWTYNAELLVLMENERTLDFHDSNVRNLYDKVRLQLR

**AAN17257.1** FIEGGWQGMVDGWYGYHHSNEQGSGYAADKESTQKAIDGITNKVNSIIDKMNTQFEVVGKEFNNLERRIENLNKKMEDGFLDVWTYNAELLVLMENERTLDFHDSNVRNLYDKVRLQLR

**AAN17256.1** FIEGGWQGMVDGWYGYHHSNEQGSGYAADKESTQKAIDGITNKVNSIIDKMNTQFEVVGKEFNNLERRIENLNKKMEDGFLDVWTYNAELLVLMENERTLDFHDSNVRNLYDKVRLQLR

**AAN17255.1** FIEGGWQGMVDGWYGYHHSNEQGSGYAADKESTQKAIDGITNKVNSIIDKMNTQFEVVGKEFNNLERRIENLNKKMEDGFLDVWTYNAELLVLMENERTLDFHDSNVRNLYDKVRLQLR

**AAN17254.1** FIEGGWQGMVDGWYGYHHSNEQGSGYAADKESTQKAIDGITNKVNSIIDKMNTQFEVVGKEFNNLERRIENLNKKMEDGFLDVWTYNAELLVLMENERTLDFHDSNVRNLYDKVRLQLR

**AAG38534.1** FIEGGWQGMVDGWYGYHHSNEQGSGYAADKESTQKAIDGITNKVNSIIDKMNTQFEAVGKEFNNLERRIENLNKKMEDGFLDVWTYNAELLVLMENERTLDFHDSNVKNLYDKVRLQLR

**AAF02306.1** FIEGGWQGMVDGWYGYHHSNEQGSGYAADKESTQKAIDGVTNKVNSIINKMNTQFEAVGREFNNLERRIENLNKKMEDGFLDVWTYNAELLVLMENERTLDFHDSNVKNLYDKVRLQLR

**AAL75847.1** FIEGGWQGMVDGWYGYHHSNEQGSGYAADKESTQKAIDGVTNKVNSIIDKMNTQFEAVGREFNNLERRIENLNKKMEDGFLDVWTYNAELLVLMENERTLDFHDSNVKNLYDKVRLQLR

**AAC32101.1** FIEGGWQGMVDGWYGYHHSNEQGSGYAADQESTQKAIDGVTNKVNSIINKMNTPFEAVGREFNNLERRIENLNKKMEDGFLDVWTYNAELLVLMENERTLDFHDSNVKNLYDRVRLQLR

**AAC32099.1** FIEGGWQGMVDGWYGYHHSNEQGSGYAADQESTQKAIDGVTNKVNSIINKMNTQFEAVGREFNNLERRIENLNKKMEDGFLDVWTYNAELLVLMENERTLDFHDSNVKNLYDKVRLQLR

**AAC32098.1** FIEGGWQGMVDGWYGYHHSNEQGSGYAADKESTQKAIDGVTNKVNSIINKMNTQFEAVGREFNNLERRIENLNKKMEDGFLDVWTYNAELLVLMENERTLDFHDSNVKNLYDKVRLQLR

**AAC32088.1** FIEGGWQGMVDGWYGYHHSNEQGSGYAADKESTQKAIDGVTNKVNSIINKMNTQFEAVGREFNNLERRIENLNKKMEDGFLDVWTYNAELLVLMENERTLDFHDSNVKNLYDKVRLQLR

**AAC32078.1** FIEGGWQGMVDGWYGYHHSNEQGSGYAADQESTQKAIDGVTNKVNSIINKMNTQFEAVGREFNNLERRIENLNKKMEDGFLDVWTYNTELLVLMENERTLDFHDSNVKNLYDKVRLQLR

**AAR99628.1** FIEGGWQGMVDGWYGYHHSNEQGSGYAADKESTQKAIDGVTNKVNSIIDKMNTQFEAVGREFNNLERRIENLNKKMEDGFLDVWTYNAELLVLMENERTLDFHDSNVKNLYDKVRLQLR

**AAD13568.1** FIEGGWQGMVDGWYGYHHSNEQGSGYAADKESTQKAIDGVTNKVNSIINKMNTQFEAVGREFNNLERRIENLNKKMEDGFLDVWTYNAELLVLMENERTLDFHDSNVKNLYDKVRLQLR

**AAM49555.1** FIEGGWQGMVDGWYGYHHSNEQGSGYAADKESTQKAIDGVTNKVNSIIDKMNTQFEAVGREFNNLERRIENLNKKMEDGFLDVWTYNAELLVLMENERTLDFHDSNVKNLYDKVRLQLR

**AAL31387.1** FIEGGWQGMVDGWYGYHHSNEQGSGYAADKESTQKAIDGVTNKVNSIIDKMNTQFEAVGREFNNLERRIENLNKKMEDGFLDVWTYNAELLVLMENERTLDFHDSNVKNLYDKVRLQLR

**AAL75839.1** FIEGGWQGMVDGWYGYHHSNEQGSGYAADKESTQKAIDGVTNKVNSIIDKMNTQFEAVGREFNNLERRIENLNKKMEDGFLDVWTYNAELLVLMENERTLDFHDSNVKNLYDKVRLQLR

**CAC28131.1** FIEGGWQGMVDGWYGYHHSNEQGSGYAADKESTQKAIDGITNKVNSIIDKMNTQFEAVGKEFNNLERRIENLNKKMEDGFLDVWTYNAELLVLMENERTLDFHDSNVKNLYDKVRLQLR

**AAA43082.1** FIEGGWQGMVDGWYGYHHSNEQGSGYAADKESTQKAIDGITNKVNSIIDKMNTQFEAVGKEFNNLERRIENLNKILEDGFLDVWTYNAELLVLMENERTLDFHEANVKSLYDKVRLQLK

**AAA43083.1** FIEGGWQGMVDGWYGYHHSNEQGSGYAADKESTQKAIDGITNKVNSIIDKMNTQFEAVGKEFNNLERRIENLNKILEDGFLDVWTYNAELLVLMENERTLDFHDANVKSLYDKVRLQLK

**AAC58999.1** FIEGGWQGMVDGWYGYHHSNEQGSGYAADKESTQKAIDGITNKVNSIIDKMNTQFEAVGKEFNNLERRIENLNKKMEDGFLDVWTYNAELLVLMENERTLDFHDSNVKNLYDKVRLQLR

**AAR16155.1** FIEGGWQGLVDGWYGYHHKQKQGSGYAADKESTQRAIDGITNKVNSIIDKMNTQFEAVGKEFNNLERRIENLNKKMEDGFLDVWTYNAELLVLMENERTLDLHDSNVKNLYDKVRHQLR

**AAD13574.1** FIEGGWQGMVDGWYGYHHSNEQGSGYAADKESTQKAIDGITNKVNSIIDKMNTQFEAVGKEFNNLERRIENLNKKMEDGFLDVWTYNAELLVLMENERTLDFHDSNVRNLYDKVRLQLR

**AAY57197.1** FIEGGWQGMVDGWYGYHHSNEQGSGYAADKESTQKAIDGVTNRVNSIIDKMNTQFEAVGKEFNNLERRIENLNKKMEDGFLDVWTYNAELLVLMENERTLDFHDSNIKNLYDKVRLQLK

**AAD13568.1** FIEGGWQGMVDGWYGYHHSNEQGSGYAADKESTQKAIDGVTNKVNSIINKMNTQFEAVGREFNNLERRIENLNKKMEDGFLDVWTYNAELLVLMENERTLDFHDSNVKNLYDKVRLQLR

**AAD13575.1** FIEGGWQGMVDGWYGYHHSNEQGIGYAADKESTQKAIDGITNKVNSIIDKMNTHFEVVGKEFNNLERRIENLNKKMEDGFLDVWTYNAELLVLMENERTLDFHDSNVKNLYDKVRLQLR

**AAD13572.1** FIEGGWQGMVDGWYGYHHSNRQGSGYAADKESTQKAIDGITNKVNSIIDKMNTQFEAVGKEFNNLERRIENLNKKMEDGFIDVWTYNAELLVLMENERTLDFHDSNVRNLYDKVRLQLR

**AAD13567.1** FIEGGWQGMVDGWYGYHHSNEQGSGYAADQESTQKAIDGVTNKVNSIINKMNTQFEAVGREFNNLERRIENLNKKMEDGFLDVWTYNAELLVLMENERTLDFHDSNVKNLYDKVRLQLR

**AAD13566.1** FIEGGWQGMVDGWYGYHHSNEQGSGYAADQESTQKAIDGVTNKVNSIINKMNTQFEAVGREFNNLERRIENLNKKMEDGFLDVWTYNAELLVLMENERTLDFHDSNVKNLYDKVRLQLR

**AAD13568.1** FIEGGWQGMVDGWYGYHHSNEQGSGYAADKESTQKAIDGVTNKVNSIINKMNTQFEAVGREFNNLERRIENLNKKMEDGFLDVWTYNAELLVLMENERTLDFHDSNVKNLYDKVRLQLR

**AAA43082.1** FIEGGWQGMVDGWYGYHHSNEQGSGYAADKESTQKAIDGITNKVNSIIDKMNTQFEAVGKEFNNLERRIENLNKILEDGFLDVWTYNAELLVLMENERTLDFHEANVKSLYDKVRLQLK

**AAA43083.1** FIEGGWQGMVDGWYGYHHSNEQGSGYAADKESTQKAIDGITNKVNSIIDKMNTQFEAVGKEFNNLERRIENLNKILEDGFLDVWTYNAELLVLMENERTLDFHDANVKSLYDKVRLQLK

**AAG01225.1** FIEGGWQGMVDGWYGYHHSNEQGSGYAADKESTQKAIDGVTNKVNSIIDKMNTQFEAVGREFNNLERRIENLNKKMEDGFLDVWTYNAELLVLMENERTLDFHDSNVKNLYDKVRLQLR

**AAG01215.1** FIEGGWQGMVDGWYGYHHSNEQGSGYAADKESTQKAIDGVTNKVNSIIDKMNTQFEAVGREFNNLERRIENLNKKMEDGFLDVWTYNAELLVLMENERTLDFHDSNVKNLYDKVRLQLR

**AAG01205.1** FIEGGWQGMVDGWYGYHHSNEQGSGYAADKESTQKAIDGVTNKVNSIIDKMNTQFEAVGREFNNLERRIENLNKKMEDGFLDVWTYNAELLVLMENERTLDFHDSNVKNLYDKVRLQLR

**AAG01195.1** FIEGGWQGMVDGWYGYHHSNEQGSGYAADKESTQKAIDGVTNKVNSIIDKMNTQFEAVGREFNNLERRIENLNKKMEDGFLDVWTYNAELLVLMENERTLDFHDSNAKNLYDKVRLQLR

**AAD51927.1** FIEGGWQGMVDGWYGYHHSNEQGSGYAADKESTQKAIDGVTNKVNSIIDKMNTQFEAVGREFNNLERRIENLNKQMEDGFLDVWTYNAELLVLMENERTLDFHDSNVKNLYDKVRLQLR

**AAF04720.1** FIEGGWQGMVDGWYGYHHSNEQGSGYAADKESTQKAIDGITNKVNSIIDKMNTQFEVVGKEFNNLERRIENLNKKMEDGFLDVWTYNAELLVLMENERTLDFHDSNVRNLYDKVRLQLR

**AAF04719.1** FIEGGWQGMVDGWYGYHHSNEQGSGYAADKESTQKAIDGITNKVNSIIDKMNTQFEVVGKEFNNLERRIENLNKKMEDGFLDVWTYNAELLVLMENERTLDFHDSNVRNLYDKVRLQLR

**AAT65209.1 DNAKELGNGCFEFYHKCDNECMESVKNGTYDYPQYSEEARLNREEISGVKLESMGTYQILSIYSTVASSLALAIMIAGLSLWMCSNGSLQCRICI**

**AAA43083.1** DNARELGNGCFEFYHKCDNECMESIRNGTYNYPQYSEEARLNREEISGIKLESMGIYQILSIYSTVASSLALAIMIAGLSFWMCSNGSLQCRICI

**AAR16155.1** DNARELGNGCFEFYHKCDNECMESIRNGTYNYPQYSEEARLNREEISGIKLESMGIYQILSIYSTVASSLALAIMIAGLSFWMCSNGSLQCRICI

**CAF21870.1** DNAKELGNGCFEFYHKCDDECMESVRNGTYDYPQYSEEARLNREEISGVKLESIGTYQILSIYSTVASSLALAIMVAGLSFWMCSNGSLQCRICI

**ABF58847.1** DNAKELGNGCFEFYHKCDDECMESVRNGTYDYPQYSEEARLKREEISGVKLESIGIYQILSIYSTVASSLALAIMVAGLSLWMCSNGSLQCRICI

**AAC58998.1** DNAKELGNGCLEFSHKCDNECMESVRNGTYDYPQYSEESRLNREEIDGVKLESMGTYQILSIYSTVASSLALAIMVAGLSFWMCSNGSLQCRICI

**AAC58997.1** DNAKELGNGCFEFYHKCDNECMESVRNGTYDYPQYSEESRLNREEIDGVKLESMGTYQILSIYSTVASSLALAIMIAGLSFWMCSNGSLQCRICI

**AAC58996.1** DNAKELGNGCFEFYHKCDNECMESVRNGTYDYPQYSEESRLNREEIDGVKLESMGTYQILSIYSTVASSLALAIMVAGLSFWMCSNGSLQCRICI

**AAC58994.1** DNAKELGNGCFEFYHKCDNECMESVRNGTYDYPQYSEESRLNREEIDGVKLESVGTYQILSIYSTVASSLALAIMVAGLSFWICSNGSLQCRICI

**AAC58991.1** DNAKELGNGWFEFYHKCDNECMESVRNGTYDYPQYSEESRLNREEIDGVKLESMGTYQILSIYSSVASSLALAIMAAGLSFWMCSNGSLQCRICI

**AAC34263.1** DNAKELGNGCFEFYHKCDNECMESVRNGTYDYPQYSEESRLNREEIDGVKLESMGTYQILSIYSTVASSLALAIMVAGLSFWMCSNGSLQCRICI

**BAE48684.1** DNAKELGNGCFEFYHKCDDECMESVRNGTYDYPQYSEEARLNREEISGVKLESIGTYQILSIYSTVASSLALAIMVAGLSFRMCSNGSLPSRICI

**AAV91149.1** DNAKELGNGCFEFYHKCDNECMESVRNGTYDYPQYSEEARLKREEISGVKLESIGTYQILSIYSTVACSLALAITVAGLSLWMCSNGSLQCRICI

**AAC58999.1** DNAKELGNGCFEFYHKCDNECMESVRNGTYDYPQYSEESRLNREEIDGVKLESMGTYQILSIYSTVASSLALAIMVAGLSFWMCSNGSLQCRICI

**AAP72011.1** DNAKELGNGCFEFYHKCDNECMESVRNGTYDYPQYSEESRLNREEIDGVKLESMGTYQILSIYSTVASSLALAIMIAGLSFWMCSNGSLQCRICI

**AAP72010.1** DNAKELGNGCFEFYHKCDNECMESVRNGTYKYPQYSEESKLNRKEIDGVKLESMGTYQILSVYSTVASSLALAIMIAGLSFWMCSNGSLQCRICI

**AAP72009.1** DNAKELGNGCFEFYHKCDNECMESVRNGTYDYLQYSEESRLNREEIDGVKLESMGTYQILSIYSTVASSLALAIMIAGLSFWMCSNGSLQCRICI

**AAP72008.1** DNAKELGNGCFEFYHKCDNECMESVRNGTYDYPQYSEESRLNREEIDGVKLESMGTYQILSIYSTVASSLALAIMIAGLSFWMCSNGSLQCRICI

**AAP72007.1** DNAKELGNGCFEFYHKCDNECMESVRNGTYDYPQYSEESRLNREEIDGVKLESMGTYQILSIYSTVASSLALAIMIAGLSFWMCSNGSLQCRICI

**AAP72011.1** DNAKELGNGCFEFYHKCDNECMESVRNGTYDYPQYSEESRLNREEIDGVKLESMGTYQILSIYSTVASSLALAIMIAGLSFWMCSNGSLQCRICI

**AAP72005.1** DNAKELGNGCFEFYHKCDNECMESVRNGTYDYPQYSEESRLNREEIDGVKLESMGTYQILSIYSTVASSLALAIMIAGLSFWMCSNGSLQCRICI

**AAP72004.1** DNAKELGNGCLEFYHKCDNECMESVRNGTYDYQQYSEESKLNREEIDGVKLESMGTYQILSIYSTVASSLALAIMIAGLSFWMCSNGSLQCRICI

**AAP72003.1** DNAKELGNGCFEFYHKCDNECMESVKNGTYDYPQYSEESRLNREEIDGVKLESMGTYQILSIYSTVASSLALAIMIAGLSFWMCSNGSLQCRICI

**AAP72002.1** DNAKELGNGCFEFYHKCDNECMESVKNGTYGYPQYSEESRLNREEIDGVKLESMGTYQILSIYSTVASSLALAIMIAGLSFWMCSNGSLQCRICI

**AAP72001.1** DNAKELGNGCFEFYHKCDNECMESVRNGTYDYPQYSEESRLNREEIDGVKLESMGTYQILSIYSTVASSLALAIMIAGLSFWMCSNGSLQCRICI

**AAP72000.1** DNAKELGNGCFEFYHKCDNECMESVKNGTYDYPQYSEESRLNREEIDGVKLESMGTYQILSIYSTVASSLALAIMIAGLSFWMCSNGSLQCRICI

**AAP71998.1** DNAKELGNGCFEFYHKCDNECMESVRNGTYDYPQYSEESRLNREEIDGVKLESMGTYQILSIHSTVASSLALAIMIAGLFFWMCSNGSLQCRICI

**AAP71997.1** DNAKELGNGCFEFYHKCDNECMESVRNGTYDYPQYSEESRLNREEIDGVKLESMGTYQILSIYSTVASSLALAIMIAGLFFWMCSNGSLQCRICI

**AAP71996.1** DNAKELGNGCFEFYHKCDNECMESVRNGTYDYPQYSEESRLNREEIDGVKLESMGTYQILSIYSTVASSLALAIMIAGLFFWMCSNGSLQCRICI

**AAP71995.1** DNAKELGNGCFEFYHKCDNECMESVRNGTYDYPQYSEESRLNREEIDGVKLGSMGTYQILSIYSTVASSLALAIMIAGLFFWMCSNGSLQCRICI

**AAN17270.1** DNAKELGNGCFEFYHKCDNECMESVRNGTYDYPQYSEESRLNREEIDGVKLESMGTYQILSIYSTVASSLALAIMVAGLSFWMCSNGSLQCRICI

**AAN17269.1** DNAKELGNGCFEFYHKCDNECMESVRNGTYDYPQYSEESRLNREEIDGVKLESMGTYQILSIYSTVASSLALAIMVAGLSFWMCSNGSLQCRICI

**AAN17256.1** DNAKELGNGCFEFYHKCDNECMESVRNGTYDYPQYSEESRLNREEIDGVKLESMGTYQILSIYSTVASSLALAIMVAGLSFWMCSNGSLQCRICI

**AAN17267.1** DNAKELGNGCFEFYHKCDNECMESVRNGTYDYPQYSEESRLNREEIDGVKLESMGTYQILSIYSTVASSLALAIMVAGLSFWMCSNGSLQCRICI

**AAN17266.1** DNAKELGNGCFEFYHKCDNECMESVRNGTYDYPQYSEESRLNREEIDGVKLESMGTYQILSIYSTVASSLALAIMVAGLSFWMCSNGSLQCRICI

**AAN17265.1** DNAKELGNGCFEFYHKCDNECMESVRNGTYDYPQYSEESRLNREEIDGVKLESMGTYQILSIYSTVASSLALAIMVAGLSFWMCSNGSLQCRICI

**AAN17264.1** DNAKELGNGCFEFYHKCDNECMESVRNGTYDYPQYSEESRLNREEIDGVKLESMGTYQILSIYSTVASSLALAIMVAGLSFWMCSNGSLQCRICI

**AAN17263.1** DNAKELGNGCFEFYHKCDNECMESVRNGTYDYPQYSEESRLNREEIDGVKLESMGTYQILSIYSTVASSLALAIMVAGLSFWMCSNGSLQCRICI

**AAN17262.1** DNAKELGNGCFEFYHKCDNECMESVRNGTYDYPQYSEESRLNREEIDGVKLESMGTYQILSIYSTVASSLALAIMVAGLSFWMCSNGSLQCRICI

**AAN17261.1** DNAKELGNGCFEFYHKCDNECMESVRNGTYDYPQYSEESRLNREEIDGVKLESMGTYQILSIYSTVASSLALAIMVAGLSFWMCSNGSLQCRICI

**AAN17260.1** DNAKELGNGCFEFYHKCDNECMESVRNGTYDYPQYSEESRLNREEIDGVKLESMGTYQILSIYSTVASSLALAIMVAGLSFWMCSNGSLQCRICI

**AAN17259.1** DNAKELGNGCFEFYHKCDNECMESVRNGTYDYPQYSEESRLNREEIDGVKLESMGTYQILSIYSTVASSLALAIMVAGLSFWMCSNGSLQCRICI

**AAN17258.1** DNAKELGNGCFEFYHKCDNECMESVRNGTYDYPQYSEESRLNREEIDGVKLESMGTYQILSIYSTVASSLALAIMVAGLSFWMCSNGSLQCRICI

**AAN17257.1** DNAKELGNGCFEFYHKCDNECMESVRNGTYDYPQYSEESRLNREEIDGVKLESMGTYQILSIYSTVASSLALAIMVAGLSFWMCSNGSLQCRICI

**AAN17256.1** DNAKELGNGCFEFYHKCDNECMESVRNGTYDYPQYSEESRLNREEIDGVKLESMGTYQILSIYSTVASSLALAIMVAGLSFWMCSNGSLQCRICI

**AAN17255.1** DNAKELGNGCFEFYHKCDNECMESVRNGTYDYPQYSEESRLNREEIDGVKLESMGTYQILSIYSTVASSLALAIMVAGLSFWMCSNGSLQCRICI

**AAN17254.1** DNAKELGNGCFEFYHKCDNECMESVRNGTYDYPQYSEESRLNREEIDGVKLESMGTYQILSIYSTVASSLALAIMVAGLSFWMCSNGSLQCRICI

**AAG38534.1** DNAKELGNGCFEFYHKCDNECMESVKNGTYDYPQYSEEARLNREEISGVKLESMGIYQILSIYSTVASSLALAIMIAGLSFWMCSNGSLQCRICI

**AAF02306.1** DNAKELGNGCFEFYHKCDNECMESVKNGTYDYPQYSEEARLNREEISGVKLESMGTYQILSIYSTVASSLALAIMVAGLSLWMCSNGSLQCRICI

**AAL75847.1** DNAKGLGNGCFEFYHKCDNECMESVKNGTYDYPQYSEEARLNREEISGVKLESMGTYQILSIYSTVASSLALAIMVAGLSLWMCSNGSLQCRICI

**AAC32101.1** DNAKELGNGCFEFYHKCDNECMESVKNGTYDYPQYSEEARLNREEISGVKLESMGTYQILSIYSTVASSLALAIMVAGLSLWMCSNGSLQCRICI

**AAC32099.1** DNAKELGNGCFEFYHKCDNECMESVKNGTYDYPQYSEEARLNREEISGVKLESMGTYQILSLYSTVASSLALAIMVAGLSLWMCSNGSLQCRICI

**AAC32098.1** DNAKELGNGCFEFYHKCDNECMESVKNGTYDYPQYSEEARLNREEISGVKLESMGTYQILSIYSTVASSLALAIMVAGLSLWMCSNGSLQCRICI

**AAC32088.1** DNAKELGNGCFEFYHKCDNECMESVKNGTYDYPQYSEEARLNREEISGVKLESMGTYQILSIYSTVASSLALAIMVAGLSLWMCSNGSLQCRICI

**AAC32078.1** DNAKELGNGCFEFYHKCDNECMESVKNGTYDYPQYSEEARLNREEISGVKLESMGTYQILSIYSTVASSLALAIMVAGLSLWMCSNGSLQCRICI

**AAR99628.1** DNAKELGNGCFEFYHKCDNECMESVRNGTYDYPQYSEEARLKREEISGVKLESIGTYQILSIYSTVASSLALAIMVAGLSLWMCSNGSLQCRICI

**AAD13568.1** DNAKELGNGCFEFYHKCDNECMESVKNGTYDYPQYSEEARLNREEISGVKLESMGTYQILSIYSTVASSLALAIMVAGLSLWMCSNGSLQCRICI

**AAM49555.1** DNAKELGNGCFEFYHKCDNECMEGVKNGTYDYPRYSEEARLNREEISGVKLESMGTYQILSIYSTVASSLALAIMVAGLSLWMCSNGSLQCRICI

**AAL31387.1** DNAKELGNGCFEFYHKCDNECMESVKNGTYDYPQYSEEARLNREEISGVKLESMGTYQILSIYSTVASSLALAIMVAGLSLWMCSNGSLQCRICI

**AAL75839.1** DNAKELGNGCFEFYHKCDNECMESVKNGTYDYPQYSEEARLNREEISGVKLESMGTYQILSIYSTVASSLALAIMVAGLSLWMCSNGSLQCRICI

**CAC28131.1** DNAKELGNGCFEFYHKCDNECMESVRNGTYDYPQYSEEARLNREEISGVKLESMGTYQILSIYSTVASSLALAIMVAGLSFWMCSNGSLQCRICI

**AAA43082.1** DNARELGNGCFEFYHKCDNECMESIRNGTYNYPQYSEEARLNREEISGIKLESMGIYQILSIYSTVASSLALAIMIAGLSFWMCSNGSLQCRICI

**AAA43083.1** DNARELGNGCFEFYHKCDNECMESIRNGTYNYPQYSEEARLNREEISGIKLESMGIYQILSIYSTVASSLALAIMIAGLSFWMCSNGSLQCRICI

**AAC58999.1** DNAKELGNGCFEFYHKCDNECMESVRNGTYDYPQYSEESRLNREEIDGVKLESMGTYQILSIYSTVASSLALAIMVAGLSFWMCSNGSLQCRICI

**AAR16155.1** DNAKELGNGCFEFYHKCDNECMESVRNGTYDYSQYSEESRLNREKIDGVKLESVGTYQILSIYSTVASSLALAIMVAGLSFWMCSNGSLQCRICI

**AAD13574.1** DNARELGNGCFEFYHKCDNECMESVKNGTYNYPQYSEEARLNREEISGVKLESMGTYQILSIYSTVASSLALAIMIAGLSFWMCSNGSLQCRICI

**AAY57197.1** DNARELGNGCFEFYHKCDDECMESVRNGTYNYPQYSEEAKLNREEISGVKLESMGTYQILSIYSTVASSLALAIMIAGLSFWMCSNGSLQCRICI

**AAD13568.1** DNAKELGNGCFEFYHKCDNECMESVKNGTYDYPQYSEEARLNREEISGVKLESMGTYQILSIYSTVASSLALAIMVAGLSLWMCSNGSLQCRICI

**AAD13575.1** DNAKELGNGCFEFYHKCDDDCMESVRNGTYDYPQYSEESRLNREEIDGVKLESMGTYQILSIYSTVASSLALAIMVAGLSFWMCSNGSLQCRICI

**AAD13572.1** DNAKELGNGCFEFYHKCDNECMESVRNGTYDYPQYSEESRLNREEIDGVKLESMGTYQILSIYSTVASSLALAIMVAGLSFWMCSNGSLQCRICI

**AAD13567.1** DNAKELGNGCFEFYHKCDNECMESVKNGTYDYPQYSEEARLNREEISGVKLESMGTYQILSIYSTVASSLALAIMVAGLSLWMCSNGSLQCRICI

**AAD13566.1** DNAKELGNGCFEFYHKCDNECMESVKNGTYDYPQYSEEARLNREEISGVKLESMGTYQILSIYSTVASSLALAIMVAGLSLWMCSNGSLQCRICI

**AAD13568.1** DNAKELGNGCFEFYHKCDNECMESVKNGTYDYPQYSEEARLNREEISGVKLESMGTYQILSIYSTVASSLALAIMVAGLSLWMCSNGSLQCRICI

**AAA43082.1** DNARELGNGCFEFYHKCDNECMESIRNGTYNYPQYSEEARLNREEISGIKLESMGIYQILSIYSTVASSLALAIMIAGLSFWMCSNGSLQCRICI

**AAA43083.1** DNARELGNGCFEFYHKCDNECMESIRNGTYNYPQYSEEARLNREEISGIKLESMGIYQILSIYSTVASSLALAIMIAGLSFWMCSNGSLQCRICI

**AAG01225.1** DNAKELGNGCFEFYHKCDNECMESVKNGTYDYPQYSEEARLNREEISGVKLESMGTYQILSIYSTVASSLALAIMVAGLSLWMCSNGSLQCRICI

**AAG01215.1** DNAKELGNGCFEFYHKCDNECMESVKNGTYDYPEYSEEARLNREEISGVKLESMGTYQILSIYSTVASSLALAIMVAGLSLWMCSNGSLQCRICI

**AAG01205.1** DNAKELGNGCFEFYHKCDNECMESVKNGTYDYPQYSEEARLNREEISGVKLESMGTYQILSIYSAVASSLALAIMVAGLSLWMCSNGSLQCRICI

**AAG01195.1** DNAKELGNGCFEFYHKCDNECMESVKNGTYDYPQYSEEARLNREEISGVKLESMGTYQILSIYSTVASSLALAIMVAGLSLWMCSNGSLQCRICI

**AAD51927.1** DNAKELGNGCFEFYHKCDNECMESVKNGTYDYPQYSEEARLNREEISGVKLESMGTYQILSIYSTVASSLALAIMVAGLSLWMCSNGSLQCRICI

**AAF04720.1** DNAKELGNGCFEFYHKCDNECMESVRNGTYDYPQYSEESRLNREEIDGVKLESMGTYQILSIYSTVASSLALAIMVAGLSFWMCSNGSLQCRICI

**AAF04719.1** DNAKELGNGCFEFYHKCDNECMESVRNGTYDYPQYSEESRLNREEIDGVKLESMGTYQILSIYSTVASSLALAIMVAGLSFWMCSNGSLQCRICI

**Olfactory receptor sequences**

**Set 1:**

**NP_001011738.1** **M----KIFSSPS-----------------------NS-STITGFILLGFPCPREGQILLFVLFSIVYLLTLMGNASIICAVYCDQKLHIPMYLLLANFSFLEIWYVTSTVPNMLA**

**NP_064686.1** MNSKASMLGTNFT--II----HPTVFILLG------------------IPGLEQYHTWLSIPFCLMYIAAVLGNGALILVVLSERTLHEPMYVFLSMLAGTDILLSTTTVPKTLA

**AAD27596.2** MPEKMLSKLIAYL--LLIESCRQTAQLVKGRRIWVDSRPHWPNTT--HYRELEDQHVWIAIPFCSMYILALVGNGTILYIIITDRALHEPMYLFLCLLSITDLVLCSTTLPKMLA

**NP_038648.2** MIK-FNG------SVF---------------------MPSVLTLV—GIPGLESVQ-CWIGIPFCVMYIIAMIGNSLILVIIKSEKSLHIPMYIFLAILAVTDIALSTCILPKMLG

**NP_667256.1** MIRRQHMEAQSNTSSIL-----APDFLLIC------------------FPNYQTWQHWLSLPLSLLFLLAMGANATLLITIRMEASLHEPMYYLLSLLSLLDIVLCLTVIPKVLA

**AAK00590.1** MF---QILRDSNSSRFQ-----VSEFILMG------------------FPGIHSWQHWLSLPLALLYVLALIANILIVTVIYQEASLHQPMYHFLGILAIVDVGLATTIMPKILA

**NP_001011738.1 NFLSDTKVISFSGCFLQFYFFFSLGSTECFFLAVMAFDRYLAICRPLHYPALMTGRL---CNILVISCWILGFLWFPVPIIIISQVSFCGSRIIDHFLCDPGPLLALTCKKSPLI**

**NP_064686.1** IFWFHAGEIPFDACIAQMFFIHVAFVAESGILLAMAFDRYVAICTPLRYSAVLTPMAIGK-MTLAIWGRSIGTI-FPIIFLLK-RLSYCRTNVIPHSYCEHIGVARLACADITVN

**AAD27596.2** IFWLRSHVISYHGCLTQMFFVHAVFATESAVLLAMAFDRYVAICRPLHYTSILNAVVIGK-IGLACVTRGLLFV-FPFVILIE-RLPFCGHHIIPHTYCEHMGIAKLACASIKPN

**NP_038648.2** IFWFHMPQISFDACLLQMELIHSFQATESGILLAMALDRYVAICNPLRHATIFS-PQLTTCLGAGALLRAFILV-SPSILLIKCRLKYFRTTIISHSYCEHMAIVKLAAQDIRIN

**NP_667256.1** IFWFDNKSIGFSSCFLQMFVMNSFLTMESCTFMVMAYDRYVAICKPLQYPSIITDQFVVR-AAIFVAARNGILT-MPIPI-LSSQLRYC-ARIIRNCICTNMSVSKLSCDDITFN

**AAK00590.1** ILWFNDNNISLPECFAQMYAIHCFVAMESGIFVCMAIDRYVAICKPLRYSSIVTESFVVK-ATVIMAIRNFVAP-MSVPV-LAAQRNYCFQNKIEHCLCSNLGVTSLACDDRKIN

**NP_001011738.1 ELVFSILSPLPLIIPFVFIMGSYTLVLAAVLKVPSASGKRKAFSTCGSHLAVVALFYGSVLVMYGSPTSE—HEAGMQKIVTLFYSVLTPLLNPVIYSLRNKHMKIALKEILRKIK**

**NP_064686.1** IWYGFSVPMASVLVDVALIGISYTLILQAVFRLPSQDARHKALNTCGSHIGVILLFFIPSFFTFLTHRFGKNIPHHVHILLANLYVLVPPMLNPIIYGAKTKQI----------R

**AAD27596.2** TIYGLTVALSVTGMDVVLIATSYILILQAVLRLPSKDAQFRAFSTCGAHICVILVFYIPAFFSFFTHRFGHHVPPQVHIILANLYLLVPPVLNPLVYGINTKQI----------R

**NP_038648.2** KICGLLVAFAILGFDIVFITFSYVRIFITVFQLPQKEARFKAFNTCIAHICVFLQFYLLAFFSFFTHRFGAHIPPYVHILLSDLYLLVPPFLNPIVYGVKTKQIRDQVLKMLFSK

**NP_667256.1** KLYQFVIGWTLLGSDLILIVLSYSFILKAVLRIKAEGAVAKALSTCGSHFILILFFSTVLLVLVITNLARERIPPDVPILLNILHHLIPPALNPIVYGVRTREIKQGIRNLLRRR

**AAK00590.1** SINQLFLAWTLMGSDLALIMISYALILRSVLRLNSAEAASKALSTCTSHLILIFFFYTVIVVISITHSVGIKIPL-IPVLLNVLHNVIPPALNPMVYALKNKELKQGLYKVLRLD

**NP_001011738.1 NWSTKKAL---------GN**

**NP_064686.1** DSMT--RML----SVVWKS

**AAD27596.2** LRIL--DFF------VKRR

**NP_038648.2** -----KH------------

**NP_667256.1** ----------------L--

**AAK00590.1** ---------------VKEG

**Set 2 (same derivatives as set 1):**

**NP_064687.1** **MF--CHLYNENNMQVAILDSILIPSYFSFLTEMEPGNYTVVTEFILLGLTDDITVSVILFVMFLIVYSVTLMGNLNIIVLIRTSPQLHTPMYLFLSHLAFLDIGYSSSVTPIMLR**

**NP_064686.1** MNSKASMLGTNFT--II----HPTVFILLG------------------IPGLEQYHTWLSIPFCLMYIAAVLGNGALILVVLSERTLHEPMYVFLSMLAGTDILLSTTTVPKTLA

**AAD27596.2** MPEKMLSKLIAYL--LLIESCRQTAQLVKGRRIWVDSRPHWPNTT--HYRELEDQHVWIAIPFCSMYILALVGNGTILYIIITDRALHEPMYLFLCLLSITDLVLCSTTLPKMLA

**NP_038648.2** MIK-FNG------SVF---------------------MPSVLTLV—GIPGLESVQ-CWIGIPFCVMYIIAMIGNSLILVIIKSEKSLHIPMYIFLAILAVTDIALSTCILPKMLG

**NP_667256.1** MIRRQHMEAQSNTSSIL-----APDFLLIC------------------FPNYQTWQHWLSLPLSLLFLLAMGANATLLITIRMEASLHEPMYYLLSLLSLLDIVLCLTVIPKVLA

**AAK00590.1** MF---QILRDSNSSRFQ-----VSEFILMG------------------FPGIHSWQHWLSLPLALLYVLALIANILIVTVIYQEASLHQPMYHFLGILAIVDVGLATTIMPKILA

**NP_064687.1 GFLRKGTFIPVAGCVAQLCIVVAFGTSESFLLASMAYDRYVAICSPLLYSTQMSSTV---CILLVGTSYLGGWVNAWIFTGCSLNLSFCGPNKINHFFCDYSPLLKLSCSHDFSF**

**NP_064686.1** IFWFHAGEIPFDACIAQMFFIHVAFVAESGILLAMAFDRYVAICTPLRYSAVLTPMAIGK-MTLAIWGRSIGTI-FPIIFLLK-RLSYCRTNVIPHSYCEHIGVARLACADITVN

**AAD27596.2** IFWLRSHVISYHGCLTQMFFVHAVFATESAVLLAMAFDRYVAICRPLHYTSILNAVVIGK-IGLACVTRGLLFV-FPFVILIE-RLPFCGHHIIPHTYCEHMGIAKLACASIKPN

**NP_038648.2** IFWFHMPQISFDACLLQMELIHSFQATESGILLAMALDRYVAICNPLRHATIFS-PQLTTCLGAGALLRAFILV-SPSILLIKCRLKYFRTTIISHSYCEHMAIVKLAAQDIRIN

**NP_667256.1** IFWFDNKSIGFSSCFLQMFVMNSFLTMESCTFMVMAYDRYVAICKPLQYPSIITDQFVVR-AAIFVAARNGILT-MPIPI-LSSQLRYC-ARIIRNCICTNMSVSKLSCDDITFN

**AAK00590.1** ILWFNDNNISLPECFAQMYAIHCFVAMESGIFVCMAIDRYVAICKPLRYSSIVTESFVVK-ATVIMAIRNFVAP-MSVPV-LAAQRNYCFQNKIEHCLCSNLGVTSLACDDRKIN

**NP_064687.1 EVIPAISSGSIIVVTVFIIALSYVYILVSILKMRSTEGRQKAFSTCTSHLTAVTLFFGTITFIYVMPQSS--YSTDQNKVVSVFYTVVIPMLNPLIYSFRNKEVKEAMKKLIAKT**

**NP_064686.1** IWYGFSVPMASVLVDVALIGISYTLILQAVFRLPSQDARHKALNTCGSHIGVILLFFIPSFFTFLTHRFGKNIPHHVHILLANLYVLVPPMLNPIIYGAKTKQI----------R

**AAD27596.2** TIYGLTVALSVTGMDVVLIATSYILILQAVLRLPSKDAQFRAFSTCGAHICVILVFYIPAFFSFFTHRFGHHVPPQVHIILANLYLLVPPVLNPLVYGINTKQI----------R

**NP_038648.2** KICGLLVAFAILGFDIVFITFSYVRIFITVFQLPQKEARFKAFNTCIAHICVFLQFYLLAFFSFFTHRFGAHIPPYVHILLSDLYLLVPPFLNPIVYGVKTKQIRDQVLKMLFSK

**NP_667256.1** KLYQFVIGWTLLGSDLILIVLSYSFILKAVLRIKAEGAVAKALSTCGSHFILILFFSTVLLVLVITNLARERIPPDVPILLNILHHLIPPALNPIVYGVRTREIKQGIRNLLRRR

**AAK00590.1** SINQLFLAWTLMGSDLALIMISYALILRSVLRLNSAEAASKALSTCTSHLILIFFFYTVIVVISITHSVGIKIPL-IPVLLNVLHNVIPPALNPMVYALKNKELKQGLYKVLRLD

**NP_064686.1 DSMT--RML----SVVWKS**

**AAD27596.2** LRIL--DFF------VKRR

**NP_038648.2** -----KH------------

**NP_667256.1** ----------------L--

**AAK00590.1** ---------------VKEG

**NP_064687.1** HWWS---------------

**Amyloidogenic sequences**

**J00248 -----QSPSSLSASVGDRVTITC RASQGIS--**-----**NYLA WFQQKPGKAPKSLIY AASSLQS GVPSRFSGSGSGT--DFTLTISSLQPEDFATYYC QQYNSYP--**

AAC97098 -----QSPSSLSASVGDRVTITC RASQDIS-------NYLA WFQQKPGKAPKSLIY SASNLQA GVPSNFSGGGSGT--DFTLTISSLQPEDFATYYC QQYKSYP--

AAC97099 -----QSPSSLSASVGDRVTITC RASQDIK-------NFLA WLQQKPGKAPKSLIY SASSLQS GVPSNFSGGGSGT--HFTLTITSLQPEDFATYYC QQYDSFP--

AAC97100 -----QSPSSLSASVGDRVTITC RASQDIS-------NFLA WFQQKPGNAPKSLIY AASSLQS GVPSKFSGGGSGT--NFTLTISSLQPEDFATYYC QQYNNYP--

AAC97101 -----QSPSSLSASIGDRVSITC RASQGIS-------NFLA WFQQKPGKAPKSLIY AASSLQS GVSSNFSGSGSGT--DFTLTISSLQPEDFATYYC QQYNSYP--

AAD09377 -----QSPSSVSASVGDRLTISC RASQDIS-------TYLA WFQQKPGKAPKSLIY AASNLQS GVPSNFSGSGSGI--EFTLTINSLQPEDFATYYC QQYNIYP--

P04430 -----QSPSSLSASVGDRVTITC RASQSVY-------NYVA WFQQKPGKAPKSLIY DASTLQS GVPSNFTGSGSGT--DFTLTISSLQPEDFATYYC QQYNSYP--

HAC -----QSPPSVSASVGDRVTITC RASQGIS-------NYLA WFQQKPGKAPKSLIY AASSLQS GVPSKFSGSGSGT--DFTLTISSLQPEDFATYYC QQYSSSP--

NIE -----QSPSSLPASVGDRVIITC RASQAIG-------NYLA WFQQKPGKAPKSLIY AASNLQS GVSSKFSGSGLGT--DFTLTISSLQPEDFATYYC QQYNSNP--

**X72813 (L12(2)) -----QSPSTLSASVGDRVTITC RASQSIS--**-----**SWLA WYQQKPGKAPKLLIY KASSLES GVPSRFSGSGSGT--EFTLTISSLQPDDFATYYC QQYNSYS--**

COL -----QSPSTLSASVGDRVTITC RASQTII-------NWLA WYQQKPGEAPKLLIY KASTLQS GVPSRFSGSGSGT--EFTLTISSLQPEDVATYYC QQYNSAP--

HOWE -----QSPSTLSASVGDRVTITC RASQTIS-------TSLA WYQQKPGNAPKLLIY RASSLQS GVPSTFSGSGSGT--EFTLTISSLQPDDFATYYC QHYRTYP--

ISE (LCDD) -----QSPSTLSASVGDRVTITC RASLGIN-------IWLA WYQQKPGKAPNLLIY KATNLQS GVPSRFSASGSGT--EFTLTISGLQPDDFATYYC HQDNSYP--

LW -----QSPSTLSASVGDRVTITC RASQSIT-------NYLA WYQQKAGKAPSLLIY KASTLET GVPSRFSGGGSGT--HFTLNISSLQPDDFATYYC QQYNTYP--

MCM (LCDD) -----QSPSTLSASVGDRVTITC RASQSIG-------NWNA WYQEKPGQAPKLLIY KASTLES GVPSRFSGEGSGT--EFTLTISKLQPDIFATYYC QQIFSLP--

WIL2 (MCN) -----QSPSTLSASVGDRVTITC RPSQAFG-------SWLA WYQQKPGKAPELLIY RVSSLQS GVPSRFSGSGSGT--EFTLTISSLQPDDVATYYC QQANRYS--

**X93620 (O18-08) -----QSPSSLSASVGDRVTITC QASQDIS-------NYLN WYQQKPGKAPKLLIY DASNLET GVPSRFSGSGSGT--DFTFTISSLQPEDIATYYC QQYDNLP--**

AAA79238(1QP1_A) -----QSPSSLSASVGDRVTITC QASQDIS-------DYLI WYQQKLGKAPNLLIY DASTLET GVPSRFSGSGSGT--EYTFTISSLQPEDIATYYC QQYDDLP--

AAC97102 -----QSPSSLSASVGDRVTITC QASQDIN-------NYLN WYQQKPGKTPKLLIY GASNLET GVPSRFSGSGSGT--DFIFTISSLQPEDIATYYC QQYDNLP--

AAC97103 -----QSPSSLSASVGDRVTITC QASQDIS-------NYLN WYQQKPGKAPKLLID GASNLET GVPSRFSGSGSGT--DFTFTISSLQPEDIATYYC QQYDNLP--

AAD09367 -----QSPSSLSASVGDRVTITC QASQDIH-------NYLN WYQQKPGKAPKLLIY DASKLET GVPSKFSGNGSGT--GFTFTISSLQSEDIATYYC QQYNDLP--

AAD29304 -----QSPSSLSASVGDRVTITC QASQDIA-------NYLN WYQQRPGKAPKVLIY DASNLEI GVPSRFSGTGSGT--HFNFTISGLQPEDIAVYSC QQYDNLP--

AAD29303 -----QSPSSLSASVGNRVIITC QASQDIT-------NFLN WYQQKPGKAPKLLIY DASNLQK GVPSTFSGSGSGT--DFTFTISSLQPEDIATYYC QQYANLV--

AM81 -----QSPSTLSASVGDRVIITC RASQSVL-------TYLN WYQQKPGKAPKLIIY DATLLLT GVPSKFSGSGSGS--DFNISISGLQPEDFATYYC QQFDAGP--

ARN -----QSPSSLSASVGDGVTLTC QASQDIS-------DYLN WYQQKVGEAPKLLMY DASYLET GVPLRFSGSGSGT--NYSFTISSLQPEDFATYYC QQYSNLP--

BRE -----QSPSSLSASVGDRVTITC QASQDID-------NYIN WYQQKLGKAPNLLIY DASTLET GVPSRFSGSGSGT--EYTFTISSLQPEDIATYYC QQYDDLP--

CRU -----QSPSSLSASVGDRVTITC QASQDVT-------NYVN WYQQKPGKAPKLLIY DASNLET GFPSRFSGSASGT--DFTFTIINLQPEDIATYYC QQYDNLP--

DEL2 (AFS) -----QSPSSLSASVGDRVTITC QASQDIS-------NYLN WYQQKPGKAPKLLIH AASSLET GVPSRFSGSGSGT--DFSFTISSLQPEDLATYYC QQYDNLP--

EPP -----QSPSSLSASVGDRVTITC QASHDIS-------DHLN WFQQKPGKAPKLLIY DASNLER GVPSRFSGGGSGT--DFTFTISSLQPEDIATYYC QQYDDLP--

ES305 -----QSPSSLSASVGDRVTITC QASEAID-------NYLN WYQQKPGKAPKLLIY NSSNLQT GVPSRFSGSGSGT--EFTFTISSLQPEDFATYYC QHYHNLP--

GRAV -----QSPSSLSASVGDIVTITC QASQAIN-------KFLN WYQQKPGKAPKLLIY AASNLET GVPLRFSGSGSGT--DFTFTISSLQPEDIATYFC QQYDNLP--

GRI -----QSPSSLSASVGDRVTITC QASQDIS-------SYLN WYQQKPGKAPELLIY AGSTLET GVPSRFSGSGSGT--DFTFTISSLQPEDVATYYC QQYLNLI--

HIG -----QSPSSLSASVGDSVTITC QADQDIN-------NSLN WYQQKQGKAPKLLIY DVFHLET GVPSRFSGSGSGT--DFTFTISSLQPEDFATYFC QQYNNLP--

MALF -----QSPSSLSASVGDRVTITC QASQDIS-------DYVN WYQQKPGKAPKLLIY DASNLQT GVPSRFSGSGSYT--HFTVTIDRLQAEDIATYYC QQNNDFP--

MH -----QSPSYLSASVGDRVNITC QASQDIS-------DHLN WYQQKPGRAPNLLIF GASSLET GVTPRFIGRGYGT--HFTFTISSLEPEDVATYYC QQYFNVP--

NIG96 (AFS) -----QSPSSLSASVGDRVTITC QASQDIS-------SYLN WSQQKPGKAPKLLIF EASDLET GVPSRFSGSGSGT--DFTFTISSLLPEDIATYYC QQYDNLP--

SMA2 (MCN) -----QSPSSLSASVGDRVTITC QASQDIN-------KYLT WYQQKPGKASKLLIY DASNLET GVPSRFSGSGSGT--DFTLTIDNLQPEDIATYYC QQYVNLP--

WAT (MCN) -----QSPSSLSASVGDRVTITC RASQDIT-------NYVN WFQQRPGQAPVLLIY GASILET GVPSRFSGSGSGT--DFTFTISSLQPEDIATYYC QQYDTLP--

**X93627 (O12-O2) -----QSPSSLSASVGDRVTITC RASQSIS-------SYLN WYQQKPGKAPKLLIY AASSLQS GVPSRFSGSGSGT--DFTLTISSLQPEDFATYYC QQSYSTP--**

AM107 -----QSPSSLSASVGDRVTITC RASQTSS-------LDLN WYQQKPGKAPKLLIY ADSSLQT GVPSRFSGSGSGT--DFTLTISGLQPEDFATYFC QQYDTGP--

AM113 -----QSPSSLSASVGNRVTITC RASQSSV-------DYVA WYQQKPGKAPKLLIF DASSLQS GVPSRFSGTGSGT--DFTLTISSLQPEDFATYYC QQFDNLP--

AND -----QSPSSLSASVGDRVTITC RASHSIG-------GSLN WYHQKPGKAPSLLIF GASSLRS GVPSRFSGSGSGT--DFTLTITSLQPEDFAAYFC QQSYSSP--

CHEB (AFS) -----QSPSSLSASVGDRVTFTC RASQTIA-------TFLN WYQQKPGKAPKLLIY GASSLQS GVPSRFSGSGSGT--DFTLTISSLQPEDFATYYC QQSYSIP--

COZ -----QSPSSLAASVGDRVTITC RARQSIS-------NYLN WYQHKPGKAPNVLIY AASNLQS GVPSGFSGSGSGT--EFTLTITSLQPEDFATYYC QQSYSAP--

CRO -----QSPSSLSASVGDRVTITC RASQSFN-------NFLN WYQQKPGKAPKLLIY DASTLES GVPSRFSGSGSGT--DFTLTISSLQPEDFATYYC QQTYTGI--

CRO2 (MCN) -----QSPSSLSASVGDRVSVTC RASQSIS-------SYLH WYQQKPGKAPELLIY GASSLQN GVPSRFSGIGSGT--DFTLTISNLQPEDFATYYC QQTYSSP--

DRU (MCN) -----QSPSSLSAFVGDRVTITC RASQSIS-------TYLN WYQQKPGKAPELLIY AVSTLQS GVPSRFSGSGSGT--DFTLTISSLQPEDFASYYC QQSYSMP--

MEV -----QSPSSLSASVGDRVIITC RASQSSV-------DYLN WYQQKPGKAPKLLIF DTSNLQS GVPSRFSGGRSGT--DFTLTISSLQPDDFATYYC QQSYTNP--

MUM -----QSPSSLSASVGDRVTITC RASQSIN-------SYLN WYQQKPGKAPNLLIY DASSLQS GVPSNFSGSGSGT--DFTLTIRSLQREDFATYYC QQSYSIP--

TRE (AFS) -----QSPSSLSASVGDRVTITC RASQSIA-------GYLN WYQQRPGKAPELLIY AASTLRS GVPSRFSGSGSGA--DFTLTISSLQPEDSATYYC QQSYSYP--

TRO2 (AFS) -----QSPSSLSASVGDRVTITC RASQSNI-------SYLN WYQQKPGKAPKLLMY AASSLQS GVPARFVGSGSGT--DFTLTISSLQPEDFATYYC QQTYSAP--

**X93632 (A19-A3) -----QSPLSLPVTPGEPASISC RSSQSLLH--SNGYNYLD WYLQKPGQSPQLLIY LGSNRAS GVPDRFSGSGSGT--DFTLKISRVEAEDVGVYYC MQALQTP--**

K2HUTW -----QSPLSLPVTPGEPASISC RSSQSLLH--SDGFDYLN WYLQKPGQSPZLLIY ALSNRAS GVPDRFSGSGSGT--DFTLKISRVEAEDVGVYYC MZALQAP--

DRE -----QSPLYLPVIPGEPASISC RSSQSLLH--SNGNNYLD WYLQKPGQSPKLVIY LGSNRAS GVPDRFSGSGSGT--DFTLKINKVEAEDVGVYYC IQALQTD--

INC -----QSPLSLPVTPGEPASISC KSSQSLMH--SSGDNYLD WYLQKPGQSPQIVIY LGSNRAS GVPDTFSGSGSGT--DFTLLISSVGAEDVGVYYC MQAEQTP--

NIC -----QSPLSLPVTPGEPASISC RSSQSLLH--TNGYNYLD WYLQKPGQSPQLLLY LGSNRVS GVPDRFSGSGSGT--NFTL---RVEAEDVGTYYC MQGLQIP--

TEW -----QSPLSLPVTPGEPASISC RSSQSLLH--SDGFDYLN WYLQKPGQSPQLLIY LGSNRAS GVPDRFSGSGSGT--DFTLKISRVEAEDVGVYYC MQALQAP--

**X93638 (L2-L16) -----QSPATLSVSPGERATLSC RASQSVS-------SNLA WYQQKPGQAPRLLIY GASTRAT GIPARFSGSGSGT--EFTLTISSLQSEDFAVYYC QQYNNWP--**

AAD09376 -----QSPANLSVSAGEGATLSC RASQSIS**-------**INLA WYQQRPGQAPRLLIF DASTRAP RSPDRFSGSGSGT--EFTLTISSLKSEDSATYYC QQYNNWP--

AL700 -----QSPATLSVSPGGRATLSC WASQSVT**-------**SNLA WYQQHPGQAPRLLIY ATSTRAT GTPARFSGSGSGT--QFTLTISSLQSEDFAVFYC QQYNNWP--

LIGN (LCDD) -----QSPGTLSVSPGDRASLSC RASQDVG**-------**SSLA WYQQKPGQAPRLLIY DASTRAS GLPARFSGSGSGT--EFTLTISSLQSEDFAVYYC QQHHTWP--

LONG -----QSPVTLSVSPGERATLSC RASQSVI**-------**SKLA WYQQKPGQAPRLLVY DASTRAT GIPGRVSGSGSGT--EFTLTSSGLQSEDSAVYYC QQYYRWS--

REV -----QSPATLSVSPGERATLSC RASQSVS**-------**TNLA WYQQKPGQAPRLLIY GASIRAT GISARFSGSGSGT--EFTLNITSLQSGDLALYFC QQYGDWP--

SCI -----QSPGTLSVSPGERATLSC RASQSVS-**------**YNLA WYQQKPGQAPRLLIY STSIRAT GIPARFSGSGSET--EFTLTISILQSEDFAVYYC QHYEDWP--

SO124 -----QSPATLSVSPGERVTLSC RASQSVR-**------**VNLA WYQQKPGQAPNFLIY GSTTRAT GIPARFSGSGSGT--EFNFTISSLQSEDFAVYYC QQYNNWP--

**X93640 (B3)** -----**QSPDSLAVSLGERATINC KSSQSVLY-SSNNKNYLA WYQQKPGQPPKLLIY WASTRES GVPDRFSGSGSGT--DFTLTISSLQAEDVAVYYC QQYYSTP**--

1EK3_A -----QSPDSLAVSPGERATINC KSSQNLLD-SSFDTNTLA WYQQKPGQPPKLLIY WASSRES GVPDRFSGSGSGT--DFTLTISSLQAEDVAVYYC QQYYSTP--

1LVE = LEN -----QSPDSLAVSLGERATINC KSSQSVLY-SSNSKNYLA WYQQKPGQPPKLLIY WASTRES GVPDRFSGSGSGT--DFTLTISSLQAEDVAVYYC QQYYSTP--

AAD09378 -----QSPDSLAVSLGERATINC KSSRSVLS-DSNSRNLLA WFQQKPGQPPKLLIY WASTRVS GVPDRFSGSGSGT--DFTLTISSLQAEDVAVYYC QQYYSTP--

P83593 -----QSPDSLVVSLGERATINC RSSQSVLY-SSNNKNYLA WYQQKPGQAPKLLFS WASTRES GVPDRFSGSGSGT--DFTLTIPGLQAEDVAVYYC QQYYRIP--

BLU -----QSPDSLAVSLGERATINC KSSLSVFF-SPNNKNYLA WYQQKPGQPPKLLIY WASTRES GVPDRFSGSGSGT--NFTLTISRLQAEDVAVYYC QQYYTTL--

BURN -----QSPDSLAVSLGERATINC KSSQSVLY-NSNNKNFLA WYQQKPGQPPKLLIY WASTRGS GVPDRFSGSGSGT--DFTLTITRLQAEDVAVYYC QQYYSTP--

CAB -----QSPDSLSVSLGERATINC KSSQSVLL-SFKNDNYLA WYQQKPGQSPK---Y WASTRES GVPDRFSGSGSGT--NFTLTISSLQAEDVAVYYC QQYYTTP--

FRA -----QSPDSLAVSLGERATINC KSSQSLLY-STRNKICLA WYQQKPGQTPQLLIS WASSRES GVPDRFSGSGSGT--DFSLTISSLQAEDVATYYC QQYSTAP--

KAL -----QSPDSLAVSLGERATINC KSSQSVLY-SSNNKNYLA WYQQKPGQPPKLLIY WASTRQS GVPDRFSGSGSGT--DFTLTISGLQAEDVAVYYC QQYYGTP--

REC -----QSPDSLAVSLGERATINC KSSQNVLD-ASFDTNTLA WYQQKPGQPPKLLIY WASSRES GVPDRFSGSGSGT--DFTLTISSLQAEDVAVYYC QQYYSTP--

SMA -----QSPDSLAVSLGERATINC KSSQSVLY-SSNNRNYLA WYQQKLGQPPKLLIY WASTRES GVPDRFSGSGSGT--DFTLTISSLQAEDVAVYYC HQYYSHP--

M30446 (humlv114) QSVLTQPPS-VSAAPGQKVTISC SGSSSN-----IGNNYVS WYQQLPGTAPKLLIY ENNKRPS GIPDRFSGSKSGT--SATLGITGLQTGDEADYYC GTWDSSLSA

A29700 QLVLTQPPS-VSAAPGQKVTISC SGSSSN-----IGNNYVS WYQQLPGTARKLLIY EDNKRPS GIPDRFSGSKDAT--VATLGITGLQTGDEADYYC GTWDSSLSA

AAC97078 QSVLTQPPS-VSAAPGQKVTISC SGSYSN-----IGKNYVS WYQHIPGTAPRLLIF ENNKRPS GIPDRMSASKSGP--SATLDIAGLQTGDEADYYC ATWENSLTA

S24321 (AIR) QSVLTQPPS-VSAAPGQKVTISC SGSSSN-----IANNYVS WYQQLPGAAPKLLIY ENVKRPS GIPDRFSGSKSGT--SATLGITGLQTGDEAEYYC GTWDSSLSG

ALX --VLTQPPS-VSAAPGQKVTISC SGSSSN-----IGNNSVS WYQQFPGAAPKLLIY DNHKRPS RIPDQFSGSKSGT--SATLGITGLQTGDEADYYC GTWDNSLNT

EPS --VLTQPPS-LSAAPGQRVSISC SGSSSN-----IGKNSVD WYQQLPGTAPKLLIF NNNKR-- ----RFSGSKSGT--SATLGITGLQTGDEAIYYC GTYDNRRSV

EZI -SVLTQPPS-VSAAPGQTVTISC SGSSSN-----IGNNYVS WYQQFPGTAPKLLIY DDNKRPS GIPDRFSGSKSAT--SATLGITGLQTGDEADYYC GAWDSRFSA

ZIM -SLLTQPPS-VSAAPGQKVTISC SGSSSN-----IGNNYVS WYQQLPGTAPRLLIY ENDKRPS GIPDRFSGSKSDA--SVTLGITGLQTGDEADYYC GTWDSSLSA

**Z22188 (HUMLV1L1) -----QPPS-ASGTPGQRVTISC SGSSSN-----IGSNTVN WYQQLPGTAPKLLIY SNNQRPS GVPDRFSGSKSGT--SASLAISGLQSEDEADYYC AAWDDSLNG**

Cox **-----**QPPS-ASGTSGQRVTISC SGSSSN**-----**IGSNQVN WYRHLPGTAPKLVIY SDSQRPS GVPDRISASKSGT--SASLAISGLQSEDESDYYC ASWDDSLDG

Mcph **-----**QPPS-ASGTPGQRVTIAC SGSSSN**-----**IGSNAVT WYQQLPGTAPQLLID TNNQRPS GVPDRFSGSKSGT--SASLAISGLQSEDEADYYC AATDDSLNG

Dia **-----**QPPS-ASGTPGQRVTISC SGSSSN**-----**IGSNVVT WYQQLPGTAPKLLIY TNNQRPS GVPGRFSGSKSGT--SASLAVSGLQSEDEADYYC ATWDDSVNG

Emm **-----**QPPS-ASGTPGQRVTISC SGSSSN**-----**IRSNTVN WYQHVPGAAPKLLIY INNQRPS GVPDRFSGSKSGT--SASLAISGLQSDDEADYYC ATWDDSLNG

Loc (MCN) **-----**QPPS-ASGTPGQRVTISC SGSSSN**-----**IGENSVT WYQHLSGTAPKLLIY EDNSRAS GVSDRFSASKSGT--SASLAISGLQPEDETDYYC AAWDDSLDV

Nig51 **-----**QPPS-ASGVPGQSVIISC SGSSSN**-----**IGRNTVN WYQQVPGAAPKLLVY SNNQWPS GVPDRFSGSKSGT--SASLAISGLHSEDEADYFC ATWDDSLDG

Nig77 **-----**QPPS-ASGTPGQRVTISC SGSTSN**-----**IGSNTVT WYQHLPGTAPKLLIY SNDQRPS GVPHRFSGSKSGA--SASLAISGLQSEDETDYYC ATWDDSLNG

Pfu **-----**QPPS-ASGTPGQRVTLSC SGGSSN**-----**LGVNTVN WYQQLPGADPKVLIY RNDQRPS GVPDRFSGSKSGT--SASLAISGLQSEDEADYHC AAWDDSLNG

A42193 **-----**QPPS-ASGTPGQRVTISC SGSSSN**-----**IGSNVVT WYQQLPGTAPKLLIY TNNQRPS GVPGRFSGSKSGT--SASLAVSGLQSEDEADYYC ATWDDSLNG

AAC97079 **-----**QPPS-ASGTPGQRVTISC SGSNSN**-----**IGINIVN WYQQVPGRAPKLLIY SNNQRPS GVADRFSGSKSGT--SASLAISGLQSEDEADYYC AAWDDSLHG

AAC97080 **-----**QPPS-ASGTPGQRVTISC SGSSSN**-----**IGSNTVT WYQHLPGAAPKLLIY SNDQRPS GVPDRFSGSKSGT--SASLAISGLQSVDEAHYYC SAWDDRLNG

AAC97081 **-----**QPPS-ASGTPGQRVTISC SGSRTN**-----**IASNTVN WYQHLPGMAPKLLIY TNDQRPS GVPDRISGSKSGT--SASLGISGLQSEDEADYYC AAWDDRLNG

AAC97082 **-----**QPPS-ASGTPGQRVSISC SGSTSN-----IGSNTVN WYLHLPGTAPKLLIY SNNQRPS GVPDRFSGSKSGT--SASLAISGLQSEDEADYYC AAWDDSLDG

AAD29289 **-----**QPPS-ASGTPGQRVTISC SGSNSN-----IGSNSVN WYQHLPGTAPKLLIY SQSQRPS GVPDRFSGSRSGT--SASLAISGLQSEDEADYYC AAWDDSLNG

AAD29290 **-----**QPPS-ASGTPGQRVIISC SGSSSN-----IGRNTVN WYQHLPGTAPKLVIY SNDQRPS GVPDRFSGSKSVT--SASLAISGLQSGDEAHSYC AAWDDSLRG

**Z22191 -----QPPS-VSAAPGQKVTISC SGSSSN-----IGNNYVS WYQQLPGTAPKLLIY DNNKRPS GIPDRFSGSKSGT--SATLGITGLQTGDEADYYC GTWDSSLSA**

AAD09370 **-----**QPPS-VSAAPGQKVTISC SGNRYN--**---**IGNNYVS WYQQFPGTAPKLLIY DDNERPS GIPDRFSGSKSGT--SATLGITGLQTGDEADYFC GTWDSGLTA

AAD29291 **-----**QPPS-VSAAPGHKVTISC SGSSSN--**---**IGKNYVS WYQQIPGTSPKLLIY DNNKRPS GIPARFSGSKSGT--SATLVITGLQSGDEADYYC GTWDSSLSD

AAD29292 **-----**QPPS-VSAAPGQNVSISC SGSSSN--**---**IGNNHVS WYQQFPGTAPKLLTY DNNERPS GIPDRFSGSKSGT--SATLVITGLQTGDEADYFC GTWDYTLS-

AAD29293 **-----**QPPS-VSAAPGQKVTISC SGSSSD--**---**IGNNYVS WYQQLPGAAPKLLIY DTSKRPS GVPDRFSGSNSGT--SATLGITGLQTGDEADYYC GTWDGSLSA

P06888 (L1HUEP) **-----**QPPS-LSAAPGQRVSISC SGSSSN**----**-IGKNYVD WYQQLPGTAPKLLIF NNNKRPS GIPDRFSGSKSGT--SATLGITGLQTGDEAIYYC GTWDNRRSV

**Z22197 -----QPAS-VSGSPGQSITISC TGTSSD----VGGYNYVS WYQQHPGKAPKLMIY EVSNRPS GVSNRFSGSKSGN--TASLTISGLQAEDEADYYC SSYTSSSTL**

AAC97083 **-----**QPAS-VSGSPGQSITISC TGTSSD-**---**VAIYNYVS WYQQYPTKAPKLTIY EVNNRPS GVSNRFSGSKSGN--TASLTISGLQAEDEADYYC SSYTITGTQ

AAC97085 **-----**QPAS-VSGSPGQSITISC TGTSSD-**---**VGGYNYVT WYQQHPGKAPNVLIY EVTYRAS GVSNRFSGSKSGN--TASLTISGLQAEDDADYYC SSYTINSTR

AAD29294 **-----**QPAS-VSGSPGQSITISC TGTSND-**---**VGGYTYVS WYQQHPGKAPKVIIY DVSNRPS GVSNRFSGSKSGN--TASLTISGLQAEDEADFYC SSYTSSSTV

AAD33349 **-----**QPAS-VSGSPGQSITISC TGTSSD-**---**IGNYNYVS WYRQHPGKAPKLIIY DVTNRPS GVSNRFSGSKSGN--TASLTISGLQAEDEADYYC NSYTTSDTL

L2HUNG = P04209 **-----**QPAS-VSGSPGQSITISC TGTTSD-**---**VGGYDFVS WYQQHPGKAPKLLIY DVNSRPS GISNRFSGSKSGN--TASLTISGLQAEDEADYYC SSFTTTNSR

JOHN **-----**QPAS-VSASPGQSITISC TGTSTD-**---**VGVYNFVS WYQQHPGKAPKVMIY DVSNRPS GVSNRFSGSKSGN--TASLTISGLQAEDEADYYC SSYTSSST-

MC **-----**QPAS-VSGSPGQSITISC TGTSSD-**---**VGGYNYVS WYQQHPGKAPQLMIY EVSNRPS GVSNRFSGSKSGN--TSSLTISGLQAEDEADYYC SSYTIGTR-

NIG84 **-----**QPAS-VSGSPGQSITISC TGTTSD-**---**VGGYDFVS WYQQHPGKAPKLLIY DVNSRPS GISNRFSGSKSGN--TASLTISGLQAEDEADYYC SSFTSTNSR

NOV **-----**QPAT-VSGSPGQSITISC TGTSSD-**---**VGDYNYVS WYQQHPGKAPKLMIY DVSDRPS GVSYRFSGSKSGN--TASLTISGLQAEDEADYYC SSYTSDST-

**Z22208 (VIII.1) -----QPPS-VSVSPGQTASITC SGDKLG-------DKYAC WYQQKPGQSPVLVIY QDSKRPS GIPERFSGSNSGN--TATLTISGTQAMDEADYYC QAWDSSTA**

AAC97087 **-----**QPPS-VSVSPGQTASITC SGDRLG**-------**DKFAC WYQQKPGQSPVLVIY EDTKRPS GIPERFSGSNSGN--TATLTISGTQAMDEGDYYC QSWDSSTA

AAD09368 **-----**QPPS-VSVSPGQTASIPC SGDKLG**-------**DKYVC WYQQKPGQSPVVVIY EDNKRPS GIPERFSGSNSGN--TATLTISGTQAMDEAYYSC QSWDTTTA

AAD09369 **-----**QPPS-LSVSPGQTASITC SGDKLE**-------**DNYVS WYQQKPGQSPVVVIN QDYKRPS GIPERFAGSNSGN--TATLTISGTQAVDEADYYC QVWASSSD

AAD09374 **-----**QPPS-VSVSPGQTATITC SGDKLG**-------**DRYVC WYQHKPGQSPALVVY QDSQRPS GIPERFSGSNSGN--TATLTISGTQGMDEADYYC QAWDSSAA

AAD29296 **-----**QPPS-VSVSPGQTASITC SGDKLG**-------**SEYVC WYQQKAGQSPVLVIY EDTKRPS GIPDRFSGSNSGN--AATLTISDTQAMDAADYYC QTWDSSTA

AAD29297 **-----**QPPS-VSVSPGQTGSITC SGEKLG**-------**DKYAC WYQQKPGQSPVVVIY QDNMRPS GIPERFSGSNFGN--TATLTISGTQAMDEADYYC QAWDSGTA

CLE (MCN) **-----**QPPS-LSVSPGQTARITC SGEKLG**-------**DAYVC WYQQRPGQSPVVVIY QDNRRPS GIPERFSGSSSGN--TATLTISGTETLDEADYYC QVWDSNAS

DOD **-----**QPPS-VSVSPGQTARITC SGDKLG**-------**DRFAS WYQQKPHQSPMLIIY QDNKRPS GIPERFSGSNSGN--TATLTISGTLAVDEADYHC EAWDGNTA

DOY **-----**QPPS-VSVSPGQTASITC SGEKLG**-------**EEYAA WYQQKPGQSPVLVIY QDTKRPS GIPERFSGSNSGN--TATLTISGTQALDEADYYC QAWDSSSV

MOL **-----**QPPS-VSVSPGQTATISC SGDKLG**-------**EXXYD WYQQSPGQSPLLVIY EGDKRPS GXXXRFSGSNSGN--TATLTISGTESMDEADYYC QAWNSSSV

NIG68 (AFS) **-----**QAPS-LSVSPGQTAYITC SGDNLG**-------**NEFVS WYQQRPGQSPALVIY DTSKRPS GIPERFSGSKSGN--TATLTISGTESMDEADYYC QAWDQIRD

NIG95 **-----**QPPS-VSVSPGQTATISC SGENLG**-------**DNFAC WYQQKPGQSPVLVIY QDNKRPS GIPERFSGSNSGN--TVTLTITGNTTMDEADYYC QVWDVGAV

WHIT **-----**QPPS-VSVSPGQTASITC SGEKLG**-------**DKYTW WYQQKTGQSPVLVIY QDTKRPS GIPERFAGSNSGN--TATLTISGTQAMDEADYYC QTWDNTRV

**Z73673 (IGLV6S1) -----QPHS-VSESPGKTVTISC TRSSGS-----IASNYVQ WYQQRPGSSPTTVIY EDNQRPS GVPDRFSGSIDSSSNSASLTISGLKTEDEADYYC QSYDSSN**

1CD0_A **-----**QPHS-VSESPGKTVTISC TRSSGN-----IDSNYVQ WYQQRPGSAPITVIY EDNQRPS GVPDRFAGSIDRSSNSASLTISGLKTEDEADYYC QSYDARN

2CD0_A **-----**QPHS-VSESPGKTVTISC TRSSGS-----IASNYVH WYQQRPGSSPTTVIF EDDHRPS GVPDRFSGSVDTSSNSASLTISGLKTEDEADYYC QSYDHNN

AAC97090 **-----**QPHS-VSESPGKTVTISC TRNSGS-----IASNYVQ WYQQRPGSSPNIVIY EDNVRPS GVPDRFSGSIDSSSNSASLTISELKTEDEADYYC QSYDNNN

AAC97091 **-----**QPHS-VSESPGKTVTISC TRSSGS-----IATSYVQ WYQQRPGSSPTTIIF EDNLRPS GVPDRFSGSIDSSSNSASLTISGLRTEDEADYYC QSYDSGK

AAC97092 **-----**QPHS-VSESPGKTVTISC TRSGGS-----IAGDYVQ WYQQRPGSAPTSVIY EDDQRPS GVPDRFSGSIDSSSNSASLTISGLKTEDEADYYC QSFDNNN

AAC97093 **-----**QPHS-VSESPGKTVTISC TGSSGS-----IASNYVQ WYQQRPGSAPTTVIY EDDQRPS GVPDRFSGSIDTSSNSASLTISRLKTEDEADYYC QSYDSNN

AAC97094 **-----**QPHS-VSESPGKTVTISC TRSSGS-----IASNYVQ WNQQRPGSAPSTVIY EDSQRPS GVPDRFSGSIDTSSNSASLTISGLKTEDEADYYC QSYDGSN

AAC97095 **-----**QPHS-VSESPGKTVTISC TRSSGS-----IASNYVQ WYQQRSGNSPSAVIY EDNQRPS GVPDRFSGSIDSSSNSASLTISGLMTEDKADYYC QSYSGSN

AAC97096 **-----**QPHS-VSESPGKSVTISC TGSSGS-----VAVNDVQ WYQQRPGSAPTTVIY DDDQRSS GVANRFSVSIDSSSNSASLTISGLKTEDEADYYC QSFDSNN

AAD28579 **-----**QPHS-VSESPGKTVTISC TRSSGS-----IVSNYVQ WYQQRPGSAPTTVIS EDDQRPS GVPDRFSGSIDSSSNSASLTISGLRTEDEADYYC QSYDSSS

AAD29298 **-----**QPHS-VSESPGKTVTISC TGSSGS-----IANNYVQ WYQQRPGSAPTTMIY EDNQRPS GVPDRFSGSIDSSSNSASLTISGLKTEDEADYYC QSFDNSK

AAD29299 **-----**QPHS-VSESPGKTNTISC TRSTGS-----IASNYVQ WYQQRPGSAPFTLIY EDNQRPS GVPDRFSGSIDSSSNSASLSISGLKTEDEADYYC QSFADFT

AAD29300 **-----**QPHS-VSESPGKTVTISC TRSSGS-----IANNYVQ WYQQRPGSSPTNVIH EDNQRPS GVPDRFSGSIDSSSNSASLTISGLKAEDEADYYC QSYDSTK

AAD29301 **-----**QPHS-VSESPGKTVTISC TRSSGN-----IASNYVQ WYQQRPASSPTTVIY EDNQRPS GVPDRFSGSIDSSSNSASLTISGLKTEDEADYYC QSYDTNI

AAK58585 **-----**QPHS-VSGSPGKTITISC TGSSGR-----IASNSVQ WYQQRPGSAPNIVMY ENNQRPS GVPDRFSGSIDSSSNSASLTISGLMTEDEADYYC QSFDDST

AAK58586 **-----**QPHS-VSESPGKTITISC TRSSGS-----IASNYVQ WYQQRPGSAPTTVIY EDNQRPS GVPDRFSGSIDSSSNSASLTISGLKTEDEADYYC QSYDSNN

AAK58587 **-----**QPHS-VSESPGKTVTISC TGSSGS-----IATNYVQ WYQLRPGSAPTTVIY EDNQRPS GVPDRFSGSIDSSSNSASLTISGLKTEDEADYYC QSYDSSI

P01721 (L6HUAR) **-----**QPHS-VSESPGKTVTFSC TGSGSI-----ASDSFVQ WYQQRPGSAPTTVIY DDNQRPS GVPDRFSGSIDDSANSASLTISGLKTEDEADYYC QSYNSNH

P06318 (L6HULT) **-----**QPLS-VSGSPEKTVTISC TGSSGS-----IGSNYVQ WYQQRPGSAPTNVIY ENNQRPS EVPDRFSGSIDSSSNSASLTISGLKTEDEADYYC QSYDNNN

CROS **-----**QPHS-VQESPGKTVTISC TRSSGS-----IASNYVQ WYQQRPGRAPTTMIY EDNQRPS GVPDRFSASIDSSSNSASLTISGLKTEDEADYYC QSYDNSN

GARN **-----**QPHS-VSESPGKTVTISC TVSGGN-----IGKNYMQ WYQQRPNSAPTTVIF ENNQRPS GVPLRFSGSIDASSNSASLTIVDLKTEDEAEYFC QTFADTN

GIO **-----**QPHS-VSESPGKTVTISC TGSSGS-----IASNFVQ WYQQRPGSAPATVIY EDNQRPS GIPDRFSGSIDSSSNSASLTISGLKTEDEADYYC QSYDSTH

JONE **-----**QPHS-VSESPGKTVTISC TRSSGS-----LASTYVQ WFQQRPGSAPTTVIY EDNQRPS GVPDRFSGSIDSSSNSASLTINGLKTEDEADYYC QSYDSET

K11 **-----**QPHS-VSDSPGKTVIISC TRSSGN-----IATNYVQ WFQQRPGSAPTTVIY DDNQRPS GVPDRFSGSIDRSSNSASLTISGLKPEDEADYYC QSYDATS

MAL2 **-----**QPHS-VSASPGKTVTISC TRSSGS-----IDTNYVQ WYQQRPGSAPTNIIY EDNQRPS GVPDRFSGSIDSSSNSASLTISGLKTEDEADYYC HSYDSST

QUI **-----**QPHS-VSESPGKTVTISC TRSSGN-----IASNYVQ WYQQRPGSSPTIVIF EDDQRPS GVPDRFSGSIDSSSNSAELTISDLKTEDESDYYC QSYDSNY

RUD **-----**QPHS-VSESPGKTVTISC TRSSGS-----IASNYVQ WYQQRPGSAPTTVIF ENAQRPS GVPDRFSGSIDSSSNSASLTISGLKTADEADYYC QSYDNSN

SUT **-----**QPHS-VSESPGKTVIISC TRSSDS-----TAGYYVQ WYQQRPGRAPTTVIF EDTQRPS GVPDRFSGSIDRSSNSASLTISGLQTEDEADYYC QSYDRDH

THO **-----**QPHS-VSESPGKTVTISC TRSSGS-----IASYYVQ WYQLRPGSAPTTVIY EDNQRPS GVPDRFSGSIDRSSNSASLTVSELKTEDEADYYC QSYDSNN

WILD **-----**QPHS-VSESPGKTVTISC TRGNGS-----IANNYVH WYQQRPGSSPTTVIF EDDHRPS GVPDRFSGSVDTSSNSASLTISGLKTEDEADYYC QSYDHNN

WIND **-----**QPHS-VSESPGKTVTISC TRSSGS-----IASYFVQ WYQQRPGSSPTTNIY EDTERPS GVPDRFSGSIDSSSNSASLTISGLRTEDEADYYC QSFDSSN

JTO (MCN) **-----**QPHS-VSESPGKTVTISC TRSSGN-----IDSNYVQ WYQQRPGSAPITVIY EDNQRPS GVPDRFAGSIDRSSNSASLTISGLKTEDEADYYC QSYDARN

**High-affinity antibody sequences**

**Light-chain sequences**

**Z22191** **-----QPPS-VSAAPGQKVTISC SGSSSN-----IGNNYVS WYQQLPGTAPKLLIY DNNKRPS GIPDRFSGSKSGT--SATLGITGLQTGDEADYYC GTWDSSLSA**

CAC06655 -----QPPS-VSAAPGQKVTISC SGSSSN-----IGNNYVS WYQQLPGTAPKLLIY DNNKRPS GIPDRFSGSKSGT--SATLGITGLQTGDEADYYC GTWDSSLS-

CAC06656 -----QPPSVSAAPGQKVTISC SGSSSN-----IGSNFVS WYQQLPGTAPKLLIY DNNKRPS GIPDRFSGSKSGT--SATLGITGLQTGDEADYYC GTWDSSLS-

CAC06657 -----QPPSVSAAPGQMVTISC SGSSSN-----IGNNYVS WYQQLRGTAPKLLIY DNNQRPS GIPDRFSGSKSGT--SATLGITGLQTGDEADYYC GTWDSSLS-

CAC06659 -----QPSSVSAAPGQKVTISC SGSTSN-----IGNNYVS WYQQLPGTAPKLLIY DNNKRPS GIPDRFSGSKSGT--SATLGITGLQTGDEADYYC GTWDSSLS-

CAC06661 -----QPPSVSAAPGQKVTISC SGSSSN-----IGNNYVS WYQQLPGTAPKLLIY DNNKRPS GISDRFSGSKSGT--SATLGITGLQTGDEADYYC GTWDSSLS-

CAC06665 -----QPPSVSAAPGQKVTISC SGSSSN-----IGNNYVS WYQQLPGTAPKLLIY GNNERPS GIPDRFSGSKSGT--SATLGITGLQTGDEADYSC GTWDSRLS-

CAC06666 -----QPPSVSAAPGQKVTISC SGSSSN-----IGNNYVS WYQQLPGTAPKLLIY DNNKRPS GIPDRFSGSKSGT--SATLGITGLQTGDEADYYC GTWDSSLS-

CAC06668 -----QPPSVSAAPGQKVTISC SGSSSN-----IGNNYVS WYQQLPGTAPKLLIY GNNERPS GIPDRFSGSKSGT--SATLGITGLQTGDEADYSC GTWDSRLS-

CAC06669 -----QPPSVSAAPGQKVSISC SGSSSN-----TGNNYVS WYQLLPGTAPKLLIY DNNERPS GIPDRFSGSKSGT--SATLGITGLQTGDEADYYC GTWDSSLS-

CAC06673 -----QPPSVSAAPGQKVTISC SGSSSN-----IGNNYVS WYQQLPGTAPKLLIY DNNKRPS GIPDRFSGSKSGT--SATLGITGLQTGDEADYYC GTWDSSLS-

CAC06674 -----QPPSVSAAPGQKVTISC SGSNSN-----IGNNFVS WYQQFPGTAPKLLIY DNDKRPS GIPDRFSGSRSGT--SATLGITGLQTGDEADFYC GTWDNSLS-

CAC06675 -----QEPSVSAAPGQKVTISC SGSSSN-----IADNYVS WYQQLPGTAPKLLIY DNNKRPS GIPDRFSGSKSGT--SATLGITGLQSGDEADYYC GTWDSSLS-

CAC06676 -----QPPSVSAAPGQKVTISC SGSSSN-----IGNNYVS WYQQLPGTAPKLLIY DNNKRPS GIPDRFSGSKSGT--SATLGITGLQTGDEADYYC GTWDSSLS-

CAC06677 -----QPPSVSAAPGQKVTISC SGSSSN-----IGNNYVS WYQQLPGTAPKLLIY DSNKRPS GIPDRFSGSKSGT--SATLGITGLQTGDEADYYC GTWDSSLS-

CAC06679 -----QPPSVSAAPGQKVTISC SGGSSD-----IGNNFVS WYQQLPGTAPKLLIY DNNKRPS GIPDRFSGSKSGT--SATLDITGLQPGDEADYYC GTWDSGLN-

CAC06680 -----QPPSVSAAPGQKVTISC SGSNSN-----IGNNFVS WYQQFPGTAPKLLIY DNDKRPS GIPDRFSGSRSGT--SATLGITGLQTGDEADFYC GTWDNSLS-

**Z22193 -----QPPSVSGAPGQRVTISC TGSSSN----IGAGYDVH WYQQLPGTAPKLLIY GNSNRPS GVPDRFSGSKSGT--SASLAITGLQAEDEADYYC QSYDSSLS-**

CAC06660 -----QPPSVSGAPGQRVTISC TGSSSN----IGSGYGVH WYQQLPGTAPKLLIY GNFNRPS GVPDRFSGSKSGT--SASLAITGLQAEDEADYYC HSYDNSLS-

CAC06663 -----QPPSVSAAPGQRVTISC TGTTSN----IGAGYDVH WYQQFPGSAPQLLFS GDSSRPA GVPDRFSASRSGT--SASLAITGLQPEDEADYFC QSFDRSKS-

CAC06664 -----QPPSVSGAPGQRVTISC TGSSSN----IGAGYDVH WYQQLPGTAPKLLIY GNSNRPS GVPDRFSGSKSGT--SASLAITGLQAEDEADYYC QSYDSSLS-

CAC06667 -----QPPSVSGAPGQRVTISC TGSSSN----IGAGYDVQ WYQQLPGTAPKVLIY GNSNRPS GVPDRFSGSKSGT--SASLAITGLQAEDEADYYC HSYDNSLS-

CAC066671 -----QPPSVSGAPGQRVTISC TGSSSN----IGAGYDVH WYQQLPGTVPKLLIY GNSNRPS GVPDRFSGSKSGT--SASLAITGLQAEDEADYYC QSYDSSLS-

CAC066682 -----QPPSVSGAPGQRATVSC TGSSSN----IGADYDVH WYQQLPGTAPKLLIY DNSNRPS GVPDRFSGSKSGT--SASLAITGLQAEDEADYYC QSYDSSLS-

CAC066683 -----QPPSVSGTPGQRVTISC TGSSSN----IGAGYDVN WYQQLPGTAPKFLIY GNTYRPS GVPDRFSGSKSGT--SASLAITGLQAEDEADYYC QSYDNSLS-

CAC06684 -----QPSSVSGAPGQRVTISC TGSSSN----IGAGYDVH WYQQLPGTAPKLLIY DNTNRPS GVPDRFSGSKSGT--SASLAITGLQAEDEADYYC QSYDSRHW-

**X93627 -----QSPSSLSASVGDRVTITC RASQSIS-------SYLN WYQQKPGKAPKLLIY AASSLQS GVPSRFSGSGSGT--DFTLTISSLQPEDFATYYC QQSYST---**

AAA61842 -----QSPSSLPASVGDRVTINC RASQSIT-------KYLA WYQQKPGEAPKLLIY DSSTLQG GVPSRVSGSGSGT--DFTLTITSLQPEDFATYHC QQAYTT---

AAA02617 -----QSPSSLSASVGDRVTITC RASQSIS-------RYLN WYQQKPGKAPKLLIY AASSLQS GVPSRFSGSGSGT--DFTLAISSLQPEDIATYYC QQSYNT---

AAA58997 -----QSPSSLSASVGDRVTITC RASQTVG-------TYLN WYQQKPGEAPKLLIY TASTLQS GVPSRFRGSGSGT--DFTLTISSLQPEDFATYYC QQSYST---

AAA02610 -----QSPSSLSASVGDRVTITC RASQSIN-------SYLN WYQQKPGKAPKLLIY AASTLLS GVPSRFSGSGSGT--DFTLTISSLQPEDFATYYC QQSYTT---

AAA61843 -----QSPSSLSASVGDRVTITC RASQTIG-------DYLN WYQDKSGKPPKVLIY AASNLHT GVPSRFSGSGSGT--HFTLTISNLQPEDFATYYC QQSYAT---

AAA58998 -----QSPSSLSASVGDRVTISC RASQNIG-------KYLN WYRQKPGKAPELLIY GTSTLQS GVPSRFSGSGSGT--DFTLTISSLQPEDFATYYC QQSYST---

AAA02605 -----QSPSSLSASVGDRVTITC RASQSIG-------RYLN WYQQKPGKAPKVLIH TASSLLS GVPSRFSGSGSET--DFTLTVSSLQPEDFATYYC QQSYDT---

AAA61841 -----QSPSSLSASVGDRVTITC RASQNIG-------RYLN WYQQKPGKAPKVLVH TASTLLS GVPSRFSGTGSGT--DFTLTVSSLQPEDFATYYC QQGYDT---

WR4.2 -----QSPSSLSASVGDRVTITC RASQSIS-------QYLN WYQQKPGKAPKLLIS ATSTLLS GVPSRFSGSGSGT--DFSLTISSLQPEDFAVYYC QQSYIT---

AAA02600 -----QSPSSLSASVGDRVTITC RASQSIS-------QYLH WYQQKPGKAPKLLIS ATSNLLS GVPSRFSGTGSGT--DFSLTISSLQPEDFAIYYC QQSYIT---

AAA02597 -----QSPSSLSASVGDRVTITC RASQSMV-------HYLH WYQQKAGKAPKLLIS ATSNLVS GVPSRFSGTGSET--DFSLTISSLQREDFATYYC QQSYTT---

AAA61838 -----QSPSSLSASLGDRITITC RASQSVG-------RYLN WYQQMPGKAPKLLIS ATSSLLS GVPSRFSGTGSET--DFSLTISSLQPEDFATYYC QQSFTT---

AAA61840 -----QSPSSLSASVGDRITITC RASQSVG-------RYLN WYQQMPGKAPKLLIS ATSSLLS GVPSRFSGTGSET--DFSLTISSLQPEDFATYYC QQSFTT---

AAA02590 -----QSPSSLSASVGDRVTITC RASQSVG-------RYLH WYQKKPGEAPKLLIS ATSDLLS GVPSRFSGTGSET--DFSLTISSLQPEDFASYYC QQSFTT---

AAA02594 -----QSPSSLSASVGDTVTITC RASQSIG-------RYLH WYHQKAGKAPKLLIS ATSNLVS GVPSRFSGTGSET--DFSLTISSLQPEDFATYYC QQSYTT---

AAA02602 -----QSPSSLSASVGDTVTITC RASQSVG-------RYLH WYQQKAGKAPKLLIS ATSNLVS GVPSRFSGTGSET--DFSLTISSLQPEDFGTYYC QQSYTT---

AAA61839 -----QSPSSLSASVGDRVTITC RASQSIG-------RYLH WYQQKAGKAPKLLIS ATSNLVG GVPSRFSGTGSET--DFSLTISSLQPEDFATYYC QQSYTT---

AAA02604 -----QSPSSLSASVGDRVTITC RASQSIG-------RYLH WYQQKAGKAPKLLIS ATSNLVG GVPSRFSGTGSET--DFSLTISSLQPEDFATYYC QQSYTT---

AAA02604 -----QSPSSLSASVGDRVTITC RASQSIG-------RYLH WYQQKAGRAPKLLIS ATSNLVG GVPSRFSGTGSET--DFSLTISSLQPEDFATYYC QQSYTT---

AAA02601 -----QSPSSLSASVGDRVTITC RASQSIG-------RYLH WYQQKAGKAPKLLIS ATSNLVS GVPSRFSGTGSET--DFSLTISSLQPEDFATYYC QQSYTT---

AAA02588 -----QSPSSLSASVGDRVTITC RASQSIG-------RYLH WYQQKAGNAPKLLIS ATSNLVG GVPSRFSGTGSET--DFSLTISSLQPEDFATYYC QQSYTT---

AAA02586 -----QSPLSLSASVGDSVTITC RASQSIG-------RYLH WYQQKAGKAPELLIS ATSNLVS GVPSRFSGTGSET--DFSLIISSLQPEDFATYYC QQSFTT---

AAA92028 -----QSPSSLSASEGDTVTITC RASENIS-------RYSN WYQQQPGKAPKLLIS AASTLQS GVPSRFSGSGSGT--HFTLTISNLQPEDFATYYC QQTYSS---

**Heavy chain sequences**

**Z14071 -----QSGAEVKKPGASVKVSCKASGYTFT GYYMH WVRQAPGQGLEWMG WINPNSGGTNYAQKFQG RVTMTRDTSISTAYMELSRLRSDDTAVYYCA**

AAA02616 -----QSGAEVKKPGASVKVSCKASGYTFT GHYMH WVRQAPGQGLEWIG WISPNRGATRFAQKFQG RVTMTSDTSINTVYMELSGLRFDDTAVYFCA

AAA68427 -----ESGAEVKKPGASVKVSCKASGYTFT GHYMH WVRQAPGQGLEWIG WISPNRGATRFAQKFQG RVTMTSDTSINTVYMELSGLRFDDTAVYYCA

AAK68009 -----QSGAELKKPGASVKVSCKASGYTFT AYYIH WVRQAPGQGLEWMG WINPNSGGTNYAQKFQG RVTMTRDTSSSTAYMDLSRLTSDDTAVYYCA

JN0295 -----ESGAEVKKPGASVKVSCKASGYTFT GHYMH WVRQAPGQGLEWIG WISPNRGATRFAQKFQG RVTMTSDTSINTVYMELSGLRFDDTAVYYCA

AAM90685 -----ESGAELKKPGASVKVSCKASGYTFT AYYIH WVRQAPGQGLEWMG WINPNSGGTNYAQKFQG RVTMTRDTSSSTAYMDLSRLTSDDTAVYYCA

**M18521 -----ESGGGLIQPGGSLRLSCAASGFTVS SNYMS WVRQAPGKGLEWVS VIY-SGGSTYYADSVKG RFTISRDNSKNTLYLQMNSLRAEDTAVYYCA**

AAA02608 -----ESGGGLIQPGGSLRLSCVVSGLNVK LNYMT WVRQAPGKGLEWVS VIF-TDGNPYYADSVRG RFTLSRDNSENTVYLQMNSLRAEDTAVYYCA

AAA02609 -----ESGGGLIHPGGSLRLSCVASGLNVK LNYMT WVRQAPGKGLEWVS VTF-TDGTTFYADSVKG RFTISRDNSDNTIYLQMNSLTADDTALYYCA

**Z12327 -----QSGAEVKKPGASVKVSCKASGYTFT SYAMH WVRQAPGQRLEWMG WINAGNGNTKYSQKFQG RVTITRDTSASTAYMELSSLRSEDTAVYYCA**

CAC06616 -----QSGAEVRKPGASVKVSCKASGYMFS SYNIH WVRQAPGQTLEWMG WIHAGTSNTKYSQKLQG RVTFTRDTSANTAYMELSGLIPEDTAIYYCA

CAC06617 -----QSGAEMKKPGATVKVSCKASGYSFS VYNIH WVRQAPGQGLEWMG WIHAGTGNRKYSQMFQD RVTISRDTSARTSYLELSSLTSEDTAVYYCA

CAC06618 -----QSGAEVKKPGASVKVSCKASGYAFA SYAIH WVRQAPGQRLEWMG WIHAGTGNRKYSQTFQD RVTLTRDTTTSTAYMELSSLKSEDTAVYYCA

CAC06619 -----QSGAEVKKPGASVRISCEASGYAFS SYDIH WVRQAPGQSLEWMG WIHGGTGNTKYSQKFQG VTFTRDTSASAAYMELTSLTSEDTAMYFCA

CAC06620 -----QSGAEVKKPGASVKVSCKTSGYAFS TYIVH WVRQAPGQRLEWMG WIHGGTGNTXYSEKFQG RVTITRDTSASAAYMEVSNLRSEDTAVYFCA

CAC06621 -----QSGAEMKKPGASVKVSCKASGYSFS IYNIH WVRQAPGQGLEWMG WIHAGTGNRKYSQVFQD RVTITRDTSASTSYMELSSLTSEDTAVYYCA

CAC06622 -----QSGAEVKKPGASVKVSCKASGYSFS IYNIH WVRQAPGQGLEWMG WIHAGTGNRKYSQVFED RVSITRDTSASTSYMELSSLTSEDTAVYYCA

CAC06627 -----QSGAEMKKPGASVKVSCKASGYSFS IYNIH WVRQAPGQGLEWMG WIHAGTGNRKYSQVFQD RVTITRDTSASTSYMELSSLTSEDTAVYYCA

CAC06628 -----QSGAEMKKPGTSVKVSCKASGYSFS IYNIH WVRQAPGQGLEWMG WIHAGTGNRKYSQVFQD RVTITRDTSASTSYMELSSLTSEDTAVYYCA

CAC06629 -----QSGAEVKKPGASVKVSCKASGYSFS IYNIH WVRQAPGQGLEWMG WIHAGTGNRKYSQVFQD RVTITRDTSASTSYMELSSLTSEDTAVYYCA

CAC06631 -----ESGAEVKKPGASVKVSCKASGYSFS SYGIH WVRQAPGQRLEWMG WINGGTGFTKYSQNFQG RVSLTRDTSASTSYLELNNLTSEDTGVYFCA

CAC06632 -----ESGAEVKKPGASVKVSCKASGYSFS SYGIH WVRQAPGQRLEWMG WINGGTGFTKYSQNFQG RVSLTRDTSASTSYLELNNLTSEDTGVYFCA

CAC06633 -----ESGAEVKKPGASVKVSCKASGYSFS SYGIH WVRQAPGQRLEWMG WINGGTGFTKYSQNFQG RVTLTRDTSTSTAYLELNSLRSEDTGLYYCA

CAC06634 -----ESGAEVKKPGASVKVSCKASGYSFS SYGIH WVRQAPGQRLEWMG WINGGTGFTKYSQNFQG RVSLTRDTSASTSYLELNNLTSEDTGVYFCA

CAC06635 -----ESGAEVKKPGASVKVSCKASGYSFS SYGIH WVRQAPGQRLEWMG WINGGTGFTKYSQNFQG RVTLTRDTSASTAYLDLNSLRSEDTGLYYCA

CAC06636 -----QSGAEVKKPGASVKVSCKASGYSFS SYGIH WVRQAPGQRLEWMG WINGGTGFTKYSQNFQG RVTLTRDTSASTAYLDLNSLRSEDTGLYYCA

CAC06638 -----ESGAEVKKPGASVKVSCKASGYSFS SYGIH WVRQAPGQRLEWMG WINGGTGFTKYSQNFQG RVTLTRDTSASTAYLELNSLRSEDTGVYYCA

CAC06640 -----QSAAEVKKPGASVKVSCKASGYSFR SYGIH WVRQAPGQRLEWMG WINGGTGFTKYSQIFQG RVTLTRDTAASTAYLELNSLRSEDTGLYYCA

CAC06642 -----ESGAEVKKPGASVKVSCKASGYSFS SYGIH WVRQAPGQRLEWMG WINGGTGFTKYSQNFQG RVSLTRDTSASTSYLELNNLTSEDTGVYFCA

CAC06643 -----QSGAEVRRPGASVKITCKASGYSFS TYGIH WVRQIPGQRPEWLG WIHAGTGATKYSRELQG RITLTRDTSATTAYMELSRLRSEDTAVYYCA

CAC06645 -----QSGAEVKKPGASVKVSCKASGYSFT PYSIH WVRQAAGQSLEWMG LIYAGTGATKYSDSWQG RVTFTRDTSASTAYMELSSLRSEDTAVYYCA

CAC06647 -----QSGAEVKKPGASVKVSCKASGYIFT GYTIH WVRQAPGQRPEWMG WVNTGSGKTKYSEKFQD RVTITRDTSASIAYMELSSLTSEDAAVYYCA

CAC06648 -----QSGAEVRRPGASVKITCKASGYSFS TYGIQ WVRQAPGQRPEWLG WIHAGTGGTKYSRKFQG RITITRDTSANTVYLDLNSLTSEDTAVYYCA

CAC06652 -----ESGAEVKKPGASVKVSCKASGYTFT GYTIH WVRQAPGQRPEWMG WVNTGNGKTKYSEKFQD RVTITRDTSASIAYMELSSLTSEDAAVYYCA

CAC06653 -----QSAAEVKKPGASVKVSCKASGYSFS SYGIH WVRQAPGQRLEWMG WINGGTGFTKYSQNFQG RVTLTRDTSASTAYLELNSLRSEDTGLYYCA

CAC06654 -----ESAAEVRRPGASVKITCKASGYSFS TYGIQ WMRQAPGQRPEWLG WIHAGTGGTKYSRKFQG RITITRDTSANTVYLDLNSLTSEDTAVYYCA
